# Migrant birds and mammals live faster than residents

**DOI:** 10.1038/s41467-020-19256-0

**Published:** 2020-11-17

**Authors:** Andrea Soriano-Redondo, Jorge S. Gutiérrez, Dave Hodgson, Stuart Bearhop

**Affiliations:** 1 Centre for Ecology and Conservation, College of Life and Environmental Sciences, University of Exeter, Cornwall Campus, TR10 9EZ Penryn, United Kingdom; 2 CIBIO/InBio, Centro de Investigação em Biodiversidade e Recursos Genéticos, Laboratório Associado, Universidade do Porto, Campus Agrário de Vairão, 4485-661 Vairão, Portugal; 3 CIBIO/InBio, Centro de Investigação em Biodiversidade e Recursos Genéticos, Laboratório Associado, Instituto Superior de Agronomia, Universidade de Lisboa, Tapada da Ajuda, 1349-017 Lisbon, Portugal; 4 Conservation Biology Research Group, Department of Anatomy, Cell Biology and Zoology, Faculty of Sciences, University of Extremadura, 06006 Badajoz, Spain

**Keywords:** Ecology, Animal migration, Behavioural ecology, Biogeography, Evolutionary ecology

## Abstract

Billions of vertebrates migrate to and from their breeding grounds annually, exhibiting astonishing feats of endurance. Many such movements are energetically costly yet there is little consensus on whether or how such costs might influence schedules of survival and reproduction in migratory animals. Here we provide a global analysis of associations between migratory behaviour and vertebrate life histories. After controlling for latitudinal and evolutionary patterns, we find that migratory birds and mammals have faster paces of life than their non-migratory relatives. Among swimming and walking species, migrants tend to have larger body size, while among flying species, migrants are smaller. We discuss whether pace of life is a determinant, consequence, or adaptive outcome, of migration. Our findings have important implications for the understanding of the migratory phenomenon and will help predict the responses of bird and mammal species to environmental change.

## Abstract (web summary)

Migration is costly. In the first global analysis of migratory vertebrates, authors report that migratory birds and mammals have faster paces of life than their non-migratory relatives, and that among swimming and walking species, migrants tend to be larger, while among flying species, migrants are smaller.

## Introduction

Animal migration is a widespread phenomenon, having evolved independently multiple times in all major vertebrate, and a number of invertebrate, classes^1^. Although it has attracted scientific interest for centuries, large components of this complex behavioural syndrome remain poorly understood^2–6^; even the definition of migration is still subject to intensive debate^7–9^. A widely accepted ecological definition is bi-directional movement between at least two areas, with a period of residency in each location. Migration is a flexible behaviour that can respond rapidly to selection and varies markedly with respect to propensity and distances travelled^10–13^. This is surprising given the sophisticated behaviours, reorganisations of internal physiology and substantial energetic outlays associated with migration in many species^14^. Therefore, it might be expected that migratory taxa should have a different pace of life to those of similar non-migratory taxa because of the trade-off among resources allocated to migration, reproduction and self-maintenance^15^. A major challenge, in this respect, is to gain a better understanding of the associations between migratory behaviour, geographic range and life history traits, among species.

A first set of hypotheses emphasise the role of ecology and biogeography in driving the evolution of migration. Based on standard life history theory^16,17^, these assume that the external context of the organism drives the evolutionary optimisation of traits that shape migratory behaviour via natural selection^8^. A range of extrinsic factors have been suggested to be important in this respect, including seasonality^18–20^, competition, predation^21^ and escape from disease^22^. However, despite intensive comparative research, there is little consensus on the traits and life histories that might be associated with migration—either as pre-adaptations or evolutionary consequences. The ubiquity of migration suggests that it must bring benefits and thus natural selection should favour the evolution of behavioural and physiological mechanisms that minimise the types of cost outlined above. Yet the patterns across studies are inconsistent, with some studies reporting reduced lifespan in migratory birds, owing to elevated (direct) mortality during migration^23–25^, and others suggesting high rates of survival throughout the annual cycle, including during migration^26^. Perhaps not surprisingly, comparative studies of the links between migration and annual reproductive outcomes have produced similarly contradictory results^27,28^. A study of North American and European land bird species found that migrants have smaller clutches than residents^27^, whereas a recent and more comprehensive study of bird species across the entire globe found the opposite^28^. The causes of such contrasting results might be partly explained by differences in taxonomic coverage (e.g., tropical taxa have slower life histories than temperate irrespective of migratory tendency). As such we still do not know whether migratory lifestyles in birds consistently places them toward the slow or fast end of the life-history continuum^26^. We know even less about how life histories might play out in migratory mammals and to date there are no comparative analyses of migratory phenomena for this taxon. To better understand how migration and life histories are linked, we thus need to consider both as dimensions of a ‘pace of life’ syndrome^15,16,29^.

A second set of hypotheses proposes that that variation in migratory propensity is predominantly a product of the allometric relationship between body size and mass-specific metabolic rates^30,31^, which are themselves linked to locomotory mode^32,33^. The body sizes of most vertebrate clades span several orders of magnitude, and this is particularly the case for mammals. Models of animal biomechanics and locomotion energetics predict that migration via walking or swimming should favour larger body sizes^32,33^. However, as with many other investigations in this area, the empirical results are ambiguous, with some studies suggesting that migratory distance is positively correlated with mass in swimmers and walkers^34^, and others not^35^. The predictions from energetic models for flying species are less consistent: some predict that migratory capacity improves with increasing body size^18^; whereas models of flapping flight migration predict that migrants should be medium-sized to small^32,33^. Other models predict that migration distances of larger flying species should not depend strongly on body mass^34^ or might even decrease with increasing body mass in flapping birds^36^.

These contrasting viewpoints on how migration might covary with biological attributes (e.g., lifespan, body size, reproductive output) and environmental influences have prevented a generalised understanding of associations between migration and life history. The conflicts likely arise from a combination of the following: limited data sets and taxonomic coverage; confounding variables (e.g., body size, biogeography, etc); or failure to consider phylogenetic patterns in migration propensity, body mass and life history.

Here, we address this using a global model of migratory tendency in mammals and birds that integrates life-history variation, biogeography, phylogeny and locomotion mode. We have compiled a comprehensive data set containing information on migratory strategy, locomotion mode, life history traits from over 700 bird and 540 mammal species. Specifically, we address the following fundamental questions: (i) what are the main axes of variation of life-history strategies in migratory and non-migratory bird and mammal species?; (ii) how are life histories and migratory propensity linked when locomotion mode, biogeography and phylogenetic patterns are accounted for?; and (iii) how do life-history strategies vary across fully migratory, partially migratory and non-migratory flying species? Predictions are difficult to generate given the contradictory evidence base, but we propose that when migration has costs in terms of survival, migrants will be selected to ensure numerical compensation by reaching sexual maturity earlier or having higher annual reproductive outputs. We also predict that life-history correlates of migration will depend on locomotory modes, such that walking, swimming and flying migrant species will have different life histories compared with their non-migrant relatives as a consequence of biomechanical constraints. Finally, although all migrant flyers should share broad life history traits, we hypothesise that partial migrants should have life histories intermediate to residents and full migrants because of the more constrained lifestyle of the latter and the relative contribution of resident and migratory individuals into the life history estimates of partially migratory species.

## Results

### Life-history axes

**Fig. 1:**
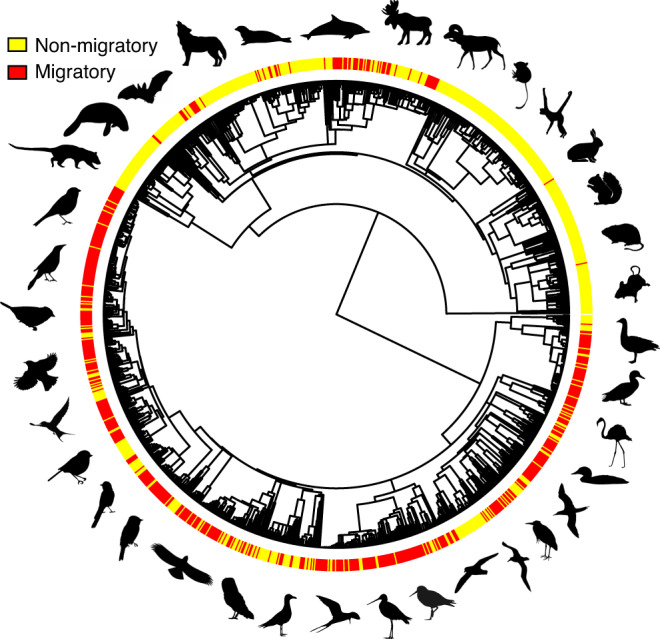
Phylogenetic tree of mammals and birds. Phylogenetic distribution of migratory species (red) and non-migratory species (yellow). Animal silhouettes were obtained from PhyloPic.

**Fig. 2:**
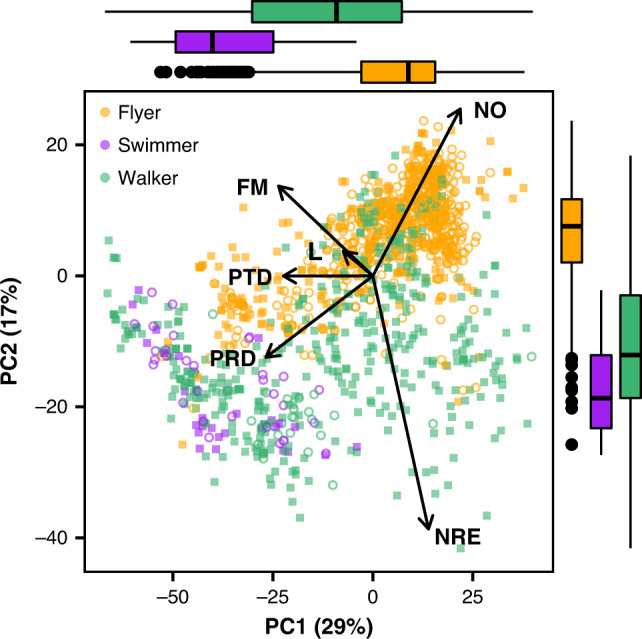
Phylogenetically corrected PCA of size-corrected life-history traits. Colours correspond to locomotory mode (flyers = orange; swimmers = purple; walkers = green; *n* = 1296 species). Solid squares represent the position of non-migratory species along the PC1 (fast–slow continuum) and PC2 (reproductive strategy) and open circles represent the position of migratory species. Arrow length indicates the eigenvectors of each size-corrected life-history trait onto PCA axes. Boxplots on the top and right represent median, upper and lower quartiles of PC1 and PC2 depending on the locomotion mode. NO = number of offspring in each reproductive event; FM = age of female sexual maturity; L = longevity, PTD = duration of postnatal development; PRD = duration of prenatal development; NRE = number of annual reproductive events.

To account for allometric associations between body size and life history strategies, we log-transformed and performed a phylogenetic size-correction of six life history traits from 1296 species of birds and mammals (Fig. 1): longevity, age at female sexual maturity, duration of prenatal development, duration of postnatal development, number of annual reproductive events and number of offspring in each reproductive event (Supporting Information S2). This was followed by a phylogenetically corrected principal components analysis (PCA) of the residuals of the six size-corrected traits (Fig. 2). The first two axes captured 46% of the variability in our selected life-history traits and were retained for subsequent analyses following Kaiser criterion (axes where eigenvalues are >1) and were consistent with previous evidence^15^. PC1 and PC2 structure was maintained when performing specific analysis for mammals and birds, while PC3 differed between classes. The first principal component (PC1) explained 28.7% of the variation, with negative loadings for adult body mass, duration of prenatal development, duration of postnatal development, age of female sexual maturity and longevity (scoring from −0.53 to −0.15). Number of offspring in each reproductive event and the number of annual reproductive events had positive loadings, of 0.44 and 0.28, respectively. Thus, this axis can be interpreted as the slow–fast pace of life continuum: species with negative scores tend to be long-lived and produce few offspring while species with positive scores tend to be shorter lived with many offspring (Fig. 2). The second principal component (PC2) axis explained 17.5% of the variability with number of offspring in each reproductive event and the number of annual reproductive events showing opposing loadings, 0.51 and −0.77, respectively. This axis is therefore a summary of reproductive strategy and indicates a negative correlation between the number of annual reproductive events and number of offspring in each reproductive event (Fig. 2).

We checked the consistency of these principal components using separate PCAs for each of the three locomotory strategies (flying, swimming and walking) and two classes (birds and mammals). Flying species include birds and bats, whereas swimming and walking species include only mammals. Results were similar: for flyers and walkers and birds and mammals, PC1 represented the fast–slow continuum and PC2 the reproductive strategy with axes explaining 46% of the variability among flying species and birds, 50% among walkers and 49% among mammals. For swimmers, PC1 (34%) captured the fast–slow continuum but the negative association between number of annual reproductive events and number of offspring in each reproductive event was not captured by any axis (Supporting Information S2).

### Migratory strategy for mammals and birds

**Fig. 3:**
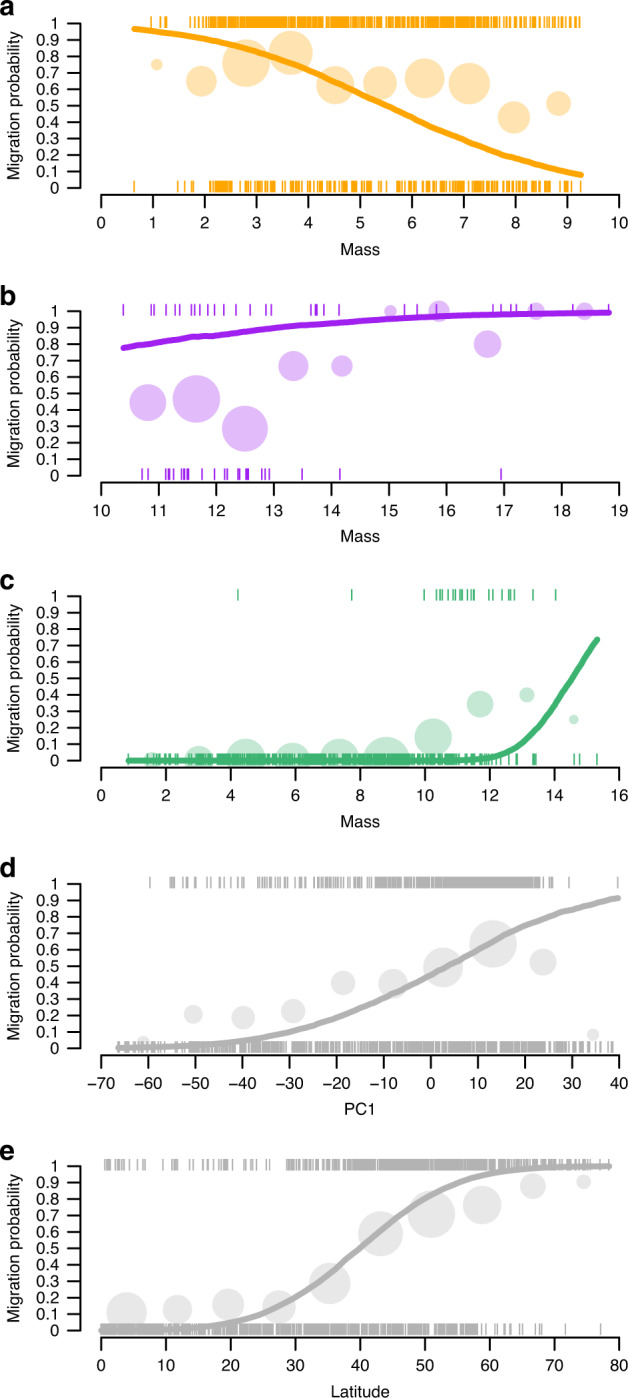
Relationships between migratory strategies and life history traits and distribution. Migratory propensity as a function of mass for **a** flyers, **b** swimmers and **c** walkers; and as a function of **d** PC1 (fast–slow continuum) and **e** absolute latitude (*n* = 1296 species). The solid line corresponds to the median predicted values from the posterior distribution from the Bayesian phylogenetic probit mixed-effects model. Circles represent the proportion of migratory species in each *x* axis bin. Vertical lines represent the position of each species along the *x* axis as a function of their migratory strategy.

To understand how life histories link to migratory propensity, we regressed migratory behaviour (resident vs migratory) against locomotion mode, pace of life (PC1), reproductive strategy (PC2), body mass and latitude. Given that the route of causality is not clear, we also regressed pace of life against migratory behaviour, locomotion mode, body mass and latitude. Our results showed that pace of life and migratory probability were positively associated, i.e., species with a faster pace of life were more likely to be migrants in mammals and birds, and migrant species tended to have faster pace of life (Table 1, Fig. 3 and Supporting Information S5).

**Table 1:** Posterior distributions of the parameter estimates of the Bayesian phylogenetic probit mixed-effects models for all species.

	Full model			Final model		
	Estimate *(β)*	Lower CI	Upper CI	Estimate *(β)*	Lower CI	Upper CI
*Fixed terms*						
Intercept	−0.90	−6.14	4.37	−0.90	−5.91	4.09
Loc swimming	−10.91	−24.29	3.57	−3.18	−10.46	4.57
Loc walking	−16.38	−22.66	−10.09	−13.84	−19.09	−8.42
Latitude	0.09	0.07	0.10	0.08	0.07	0.10
PC1	0.04	0.01	0.08	0.04	0.01	0.06
PC2	0.01	−0.03	0.06	0.01	−0.03	0.05
Mass	−0.38	−0.62	−0.11	−0.38	−0.62	−0.13
Swimming:PC1	−0.10	−0.21	0.02	–	–	–
Walking:PC1	0.02	−0.05	0.10	–	–	–
Swimming:PC2	0	−0.21	0.20	–	–	–
Walking:PC2	−0.09	−0.20	0.01	–	–	–
Swimming:mass	0.88	0.16	1.56	0.57	−0.03	1.07
Walking:mass	1.32	0.75	1.90	1.18	0.75	1.69
*Random terms*						
Phylogenetic variance	16.83	8.94	25.27	15.82	8.643	23.24

Moreover, the relationships between migratory propensity and mass differed among locomotory categories (the 95% credible intervals of the interaction between mass and locomotion mode did not span zero, Table 1, Fig. 3). Among flying species, there was a negative relationship between migratory propensity and mass, i.e., smaller species had a higher propensity to migrate (Fig. 3). In contrast, among swimming and walking species, the propensity to migrate was positively associated with mass (Fig. 3). Not surprisingly, absolute latitude had a positive effect on migration, i.e., species occurring in northern and southern latitudes had a higher probability of being migrants than species in the tropics (Table 1, Fig. 3). This result was maintained when only walking and flying species occurring in the Northern Hemisphere (positive latitudes) were included in the model (Supporting Information S4). Reproductive strategy (PC2) did not show a significant association with migration propensity (Table 1). This was also the case for the interactions between locomotion and PC1 and PC2, thus these were removed from the model with no qualitative impact on the overall result (Table 1). The results of the second set of models with pace of life as a response variable supported much of the above, confirming that pace of life is faster among migrants, and faster among walkers than among flyers for a given body size. (Supporting Information S5).

We checked the robustness of these results by performing separate phylogenetic regressions for each of the three locomotion groups and two classes. The locomotion-specific analyses showed qualitatively similar associations. Among flyers, migration correlated positively with pace of life and latitude and negatively with mass. Among walkers, mass, pace of life and latitude had a positive association with migration propensity. However, among swimmers the 95% credible intervals of mass, pace of life and latitude spanned zero (Supporting Information S4 and S5). The bird-only and mammal-only models also supported the findings, although the relationship between pace of life and migratory strategy was only marginally significant for mammals; this weaker association in mammals was likely driven by swimmers (see above, Supporting Information S4). To rule out the possibility that the relationship between migratory strategy and pace of life was mediated by a confounding effect with latitude we also performed a model for temperate flyers and walkers, thus restricting the potential effect of latitude. Again, we found the same positive association between migration and pace of life.

We also checked if these predictions were maintained across various groups of birds and mammals by re-fitting models for those groups where sample sizes were large enough (>20 species and >25% in the least represented strategy): Passeriformes, non-Passeriform flyers (non-Passeriform birds and Chiropters), non-Passeriform birds, Accipitriformes, Pelecaniformes, Artiodactyls and Chiropters. These significant relationships persisted in Passeriformes, non-Passeriform flyers and non-Passeriform birds Supporting Information S4). Nonsignificant outcomes tended to be associated with small sample sizes, hence might have been due to low power.

### Life histories of full- and partial-migrant fliers

**Fig. 4:**
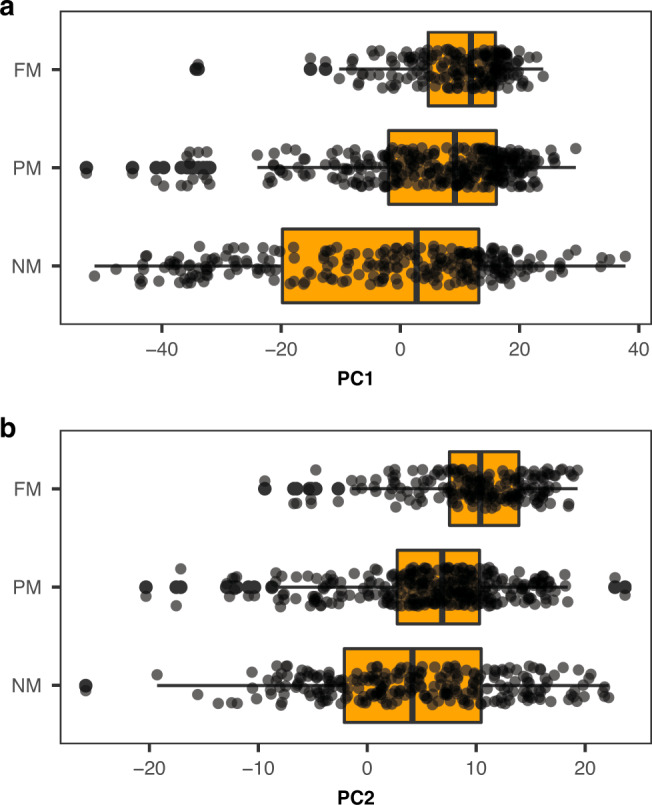
Relationships between migratory strategies and life history traits. Migratory strategy and **a** PC1 scores (fast–slow continuum) and **b** PC2 scores (reproductive strategy) for flying species (*n* = 777 species). Boxplots represent the median and the interquartile range (IQR) and the whiskers the 1.5*IQR. FM stands for fully migratory species, PM for partially migratory species and NM for non-migratory species.

To test the hypothesis that the link between migration and pace of life should differ between different types of migrants, we split flying species into fully migratory, partially migratory and non-migratory. We found as predicted that patterns for partial migrants were intermediate (Fig. 4, Table 2), full migrants had lowest body masses and fastest paces of life, followed by partial migrants and then non-migrants (Table 2).

**Table 2:** Posterior distributions of the parameter estimates of the multinomial Bayesian phylogenetic probit mixed-effects models for flyers.

	Estimate *(β)*	Lower CI	Upper CI
*Fixed terms*			
Intercept	−0.65	−6.05	4.17
Latitude	0.09	0.07	0.10
PC1	0.03	0	0.05
PC2	−0.03	−0.06	0.01
Mass	−0.51	−0.74	−0.31
*Random terms*			
Phylogenetic variance	16.72	10.51	23.83

## Discussion

We show that a migratory lifestyle is associated with a faster pace of life (higher annual fecundity, earlier maturity and shorter lifespans) across a comprehensive group of mammal and bird species spanning almost the entire globe (Figs. 3 and 4). Our results support predictions from theoretical models and empirical work that have suggested that the costs of migration—in terms of time, energy or mortality risk—should trade-off with life-history traits such as annual investment in reproduction and lifespan^1,37^.

To date, discussions of these trade-offs have mostly taken place in the literature on partial migration, within the context of understanding intraspecific variation in migratory behaviour among individuals^19^. Although this approach has yielded many valuable insights, it has limited our ability to set findings within a broader macroevolutionary framework. Our interspecific comparisons, framed in the context of alternative but functionally similar adaptations for seasonality, shed light on the evolution of the migratory phenomenon and other strategies associated with life in seasonal environments. By prioritising reproduction over survival, species with faster life histories have the potential to increase numbers more rapidly, thus mitigating the risks of increased stochastic mortality during migration^1,35^. Further, the shorter lifespan of migratory species may be an inescapable result of a hard-working lifestyle and/or increased stochastic mortality^1,29,37^. However, the idea that increased energetic output reduces lifespan remains controversial^38^. For example, high output in one part of the annual can be compensated by lowering output in others, which points to a need for comparative studies to account for the energetic costs throughout annual cycle^26^.

In addition, our analyses provide rare evidence of the manner in which body size appears to constrain migratory behaviour. We find that the association between migration and body mass depends on locomotion mode, such that migrant flyers (i.e., birds and bats) tend to have smaller body sizes than residents, whereas in migrant walkers and swimmers (i.e., all mammals except bats) it tends to be larger. The negative trend for body mass in flyers was still apparent when partial migrants were included as an intermediate category, with non-migrants being larger than partial migrants and partial migrants being larger than full migrants. These contrasting associations between migration and mass for flyers, walkers and swimmers are likely related to biomechanics. In walkers, the cost of transport per unit travelled is much higher, thus migration tends to be less common and shorter^39^. This is because, despite the fact that the speed of travel increases with body size, the concomitant allometric reductions in metabolic rates are much smaller^32,39^. As such, only large-sized walkers can store energy at high enough rates to allow them to complete long-distance migrations in time^32,39^. In swimmers, the cost of migration is smaller and long-distance migrations are more frequent, but like walkers, speed of travel increases with size and the metabolic rate changes are small^31,40^. Among flyers, fuel deposition rate is negatively correlated with body mass^40–42^. Combined with the negative relationship between speed and body size^32,39^, this will limit the ability to store fuel stores in large species. As such, large migratory birds face greater energetic problems than smaller birds when forced to use flapping flight^40^.

Multiple selection pressures are likely to play a role in driving the migratory pace of life. Survival of migrants could be affected by unseasonable cold weather in the breeding areas, which can trigger mass mortality events among migrants while residents are adapted to withstand them^43^. Moreover, there is evidence that mortality during migration can increase as a consequence of bad weather, predation or the reactivation of latent infections^25,43–45^. These factors could reduce the lifespans of migrants and ultimately exert selection for a faster pace of life. Alternatively, selection could be acting on annual reproductive outcomes. Some studies argue that migration occurs mainly as a response to seasonality on the breeding grounds^19,46^ and that migrants benefit from a seasonal availability of resources^46,47^. This peak of resources during the breeding period could boost the reproductive output in migratory birds and mammals.

Although our study establishes an adaptive link between life-history and migratory strategies, the direction of the causality in the relationship is far from clear. While life-history traits such as lifespan are typically shaped over very long time periods, there is good evidence that migratory patterns can evolve rapidly: with evidence for migratory populations becoming resident and resident ones becoming migratory within a few decades^48^. However, among birds significant morphometric differences have been observed between resident and migratory individuals in partially migratory species^49,50^. Our results highlight that when studying the evolutionary basis for migration and the underlying life history traits, it is important to consider the ecological and biomechanical selection pressures that have shaped them over evolutionary time.

Our main inferences are supported by global and locomotion-specific analyses, as well as by several group-specific analyses such as those for Passeriformes, non-Passerine flyers and non-Passerine birds, i.e., larger groups, although in the latter two these effects were relatively weak. We did not find a significant relationship between migration strategy and pace of life for swimmers, Artiodactyls, Accipitriformes, Pelecaniformes and Chiropters. There are alternative explanations for these nonsignificant outcomes: (i) that large sample sizes across different taxa are needed to generate the statistical power required to capture subtle but relevant evolutionary processes; and (ii) that the links between migratory behaviour and pace of life vary among taxa and are not necessarily straightforward. For example, the group of swimming species is mostly comprised of marine mammals, which tend to be low-fecundity species with low variability in mortality rates^51,52^. Lower variance in vital rates—fecundity, survival or growth—might thus reduce the strength of selection that might establish associations between migration and life history. It is perhaps not surprising then that finer scale comparisons may deviate from the overall trends. Indeed, a study of Passeriformes from North American boreal forests found that the few resident species that remained had higher mortality than long-migratory migratory ones^53^; highlighting the additional challenges that species occurring in higher latitudes can face. There are other factors that can covary with migratory behaviour that could also influence one of the axes of pace of life, for example precociality of young, antipredator defence, diet and nest type (in birds). For example, some authors have shown that birds breeding in open nests have higher survival than those breeding in cavities^54^. Thus, it is possible such traits could potentially influence the relationship between migratory strategy and pace of life and this warrants further investigation.

Our study also leads to a number of questions and imperatives. First, the taxonomic coverage was limited to species of birds and mammals that have been extensively studied and have available data for all the life history traits. Other classes of vertebrates lack similarly comprehensive data. Second, there is a lack of clarity around migration propensities in other vertebrate groups. This in turn highlights the urgent need for better coverage in some of these less well studied groups. We could not find any examples of migratory amphibians; and within reptiles, marine turtles were the only group with good supporting data sets, although all of this taxon is migratory which creates problems of non-independence due to their close phylogenetic relationship. Third, the analyses reported herein focus on endotherms and thus the patterns we identify might not be readily applicable to ectotherms. For example, birds and mammals are able to escape seasonally harsh environments and take advantage of seasonal resource pulses at high latitudes via migration^55^. Whereas there are some spectacular insect migrations, they do not appear to venture as far north as the endotherms described in our study, possibly because the cold limits their activity^56^. Linking the fields of physiological and broad-scale ecology should yield a better mechanistic understanding of how migration might co-evolve with life-history traits.

Given the potential links between environmental conditions on the breeding grounds and life history strategies in migrants, it is prudent to consider the potential effects of climatic and environmental change. The impact of climate change and habitat destruction on migratory patterns have been broadly studied^57^ and the former has been liked to change in migratory patterns and morphological traits in many bird species^58^; whereas habitat degradation has disrupted the migratory corridors of many walking mammals^57^. As such, if the life history linkages we observe are indeed linked to stochastic or harsh conditions during migration and breeding, climate change may weaken or alter the relationships. Moreover, the broad link between migration and pace of life that we have identified here means that, migrants (particularly those that are unable to adapt rapidly) may act as living sentinels for global environmental change in ways that are much more subtle than previously proposed^59^.

To conclude, migratory species of birds and mammals have faster paces of life than their non-migratory relatives. However, further studies should explore how this link might vary across taxonomic groups and the conditions that may reverse the trend. Moreover, the direction of causality is not clear: for a given body size, do migrants develop more quickly and have shorter lifespans or do species that develop more quickly and have shorter lifespans tend to migrate? Whichever is correct, our evidence shows that in migratory species, there is an adaptive allocation of resources away from survival and towards development and reproduction, which would be favoured by a combination of pulsed resources through time and the direct mortality costs of migration. Such patterns may help explain, at least in part, the widespread observations of migratory species in decline, particularly with respect to large body size, although the mode of action is unclear with respect to pace of life. There is increasing evidence of changes in migratory behaviour in a broad range of species and may be these that provide a route to further understanding of the way in which the association between migration and pace of life is manifest.

## Methods

### Data

We used the ‘amniotes database’^60^ to obtain data of seven life-history traits from birds and mammals: adult body mass, longevity (i.e., the median of the reported longevity data for a particular species), age of female sexual maturity, duration of prenatal development, duration of postnatal development, number of annual reproductive events and number of offspring in each reproductive event. We selected these traits to reflect the life history components of interest, whereas at the same time maximising the number of species with a complete set. We expected a clear trade-off between long development times (age of female sexual maturity, duration of prenatal development, duration of postnatal development) and number of offspring per year.

Here, we use an ecological definition of migration as a directional, seasonal, movement and return between one place and another^7–9^. For birds, we used the Eyres et al. data set^61^ to classify bird migratory strategies into migratory (fully migratory and partial migratory) and non-migratory. For mammals, we used several sources (Gnanadesikan et al.^62^, Handbook of the Mammals of the World Volumes 1–8 and species-specific literature for Chiroptera; see Supporting Information for further details) to gather information on migratory strategies.

The geographic distribution of each species was extracted from the IUCN Red List of Threatened Species. For birds, latitude was calculated as the centroid of the species range for non-migratory species or the centroid of the breeding range for migratory species. For mammals, only the overall species range was available, thus, latitude was calculated as the centroid of the overall range. We classified all birds (non-flying species are not present in our database) and the Chiroptera as flyers. Cetaceans and other sea mammals, such as seals and sea lions (order Phocidae) and sea cows (order Sirenia), were classified as swimmers. Terrestrial mammals that move by walking or running were classified as walkers.

### Phylogeny

We constructed a single species-level phylogenetic consensus tree for birds using 1000 phylogenetic trees from the BirdTree project^63^ with the *phytools* package^64^ in R (ver. 3.5.2). For mammals, we used the phylogeny of Fritz et al.^65^ a comprehensive tree available for mammals. As we needed a phylogenetic tree containing both birds and mammals for the main analysis, we made a combined tree following Healy et al.^66^; both trees were rooted at 315 million years, corresponding to the dating of *Archerpeton anthracos*, placed at the origin of amniotes.

### Phylogenetic size-correction

To avoid the confounding effects of mass on the six life history traits (longevity, age of female sexual maturity, duration of prenatal development, duration of postnatal development, number of annual reproductive events and number of offspring in each reproductive event) we remove the effect of size by regressing each life history trait against adult body mass using Bayesian phylogenetic regression to control for the evolutionary process^67,68^ using the package *MCMCglmm*^69,70^ in R (R code in Supporting Information). Life-history traits (including mass) were log-transformed prior to the least squares regression to obtain approximately normal residual distributions.

### Phylogenetic PCA

A Bartlett’s test of sphericity indicated collinearity among the size-corrected residuals from the six life-history traits (*χ*^2^ = 2733.95; *p* < 0.001) and thus that it was appropriate to apply PCA to reduce the dimensionality of the data. We performed a phylogenetically corrected PCA^67^ for the 1296 species of mammals and birds using the *phytools* package^64^. We performed locomotion-specific and class-specific PCAs to assess whether the global PCA was relevant for all locomotion groups (flyers, swimmers and walkers) and class (birds and mammals).

### Bayesian comparative analyses

To understand how life-history strategies is linked to migratory probability, we fitted two models: in the first, migratory strategy was fitted as a binary response variable (migratory or non-migratory) and locomotion mode (flying, swimming or walking), latitude (absolute value), mass, the two first PCA axes, and the interactions between locomotion mode and the PCA axis and mass as predictors. The second model had PC1 (pace of life) as a gaussian response variable and migratory strategy, locomotion mode, latitude and mass as predictors. We fitted Bayesian phylogenetic probit and gaussian mixed-effects models using the package *MCMCglmm*^69,70^ in R. This approach included a phylogenetic covariance structure to account for shared ancestry. We used weakly informative priors (normal distribution with a mean of zero and a variance of 1000). The residual variance cannot be estimated for probit models so we fixed it to 1. For each model, we ran two MCMC chains for 5 million iterations, with 100,000 as burn-in and a thinning of 2500 iterations, resulting in effective sample sizes of >1960. All models converged (Gelman and Rubin’s convergence diagnostic <1.1).

To assess whether the results were relevant for each locomotion mode, we run alternative locomotion and class-specific models that included the PCA scores from the locomotion-specific PCAs. Furthermore, to control for the potential confounding effect of latitude and migratory strategy, we implemented a model only including walking and flying species from temperate areas, species occurring in latitudes 23.5ºN to 66.5 ºN and 23.5ºS to 66.5ºS; and to assess the potential effect of transforming the latitudes to absolute values, we implemented a model for walking and flying species occurring in the Northern Hemisphere (only positive latitudes). In addition, phylogenetic groups with >20 species and a balanced number of migratory and non-migratory species (>25% in the least represented strategy) we performed group-specific analyses (Supporting Information, Appendix S4). We further checked the robustness of these by removing the largest class (Passeriformes), from bird and flyer models, creating two further categories: Non-Passeriform flyers and non-Passeriform birds.

Finally, we ran a model for flying species (birds and bats) only to explore the link between life-history strategies and whether animals are fully migratory, partially migratory or non-migratory. We fitted Bayesian phylogenetic ordinal mixed-effects models, where the response variable adopted three ordered options, fully migratory (2) <partially migratory (1) <non-migratory (0). These models included latitude, mass and the two PCA axes as predictors.

### Reporting summary

Further information on research design is available in the Nature Research Reporting Summary linked to this article.

## Supplementary information

Supplementary Information

Peer Review File

Reporting Summary

## Data Availability

Raw data used in this study are included in this published article (and its Supporting Information files). Source data are provided with this paper.

## Source data

Source Data

## Code availability

The R code used to conduct analyses is available in the Supporting Information.
