# Supplementary material for: Migrant birds and mammals live faster than residents
Source: Nat Commun. 2020 Nov 17;11:5719. doi: 10.1038/s41467-020-19256-0 (PMC7673136; doi:10.1038/s41467-020-19256-0)
Supplement: Supplementary file 1 — Supplementary Information [file 41467_2020_19256_MOESM1_ESM.pdf]

Supporting Information to 'Migrant birds and mammals live faster than residents'

Andrea Soriano-Redondo, Jorge S. Gutiérrez, Dave Hodgson, Stuart Bearhop

May 2020

Overview

This Supporting Information accompanies our submitted paper, 'Migrant birds and mammals live faster than residents'. Below we explain how we selected the dataset and classified migratory behaviour and provide the code to perform the following analyses:

- Phylogenetic size-correction
  - All species (*n = 1,296*)
  - Flyers (*n = 777*)
  - Swimmers (*n = 60*)
  - Walkers (*n = 458*)
  - Birds (*n = 756*)
  - Mammals (*n = 540*)
- Phylogenetic principal components analysis
  - All species (*n = 1,296*)
  - Flyers (*n = 777*)
  - Swimmers (*n = 60*)
  - Walkers (*n = 458*)
  - Birds (*n = 756*)
  - Mammals (*n = 540*)
- Binomial bayesian comparative analyses (Response variable: Migratory strategy)
  - All species (*n = 1,296*)
  - Flyers (*n = 777*)
  - Swimmers (*n = 60*)
  - Walkers (*n = 458*)
  - Birds (*n = 756*)
  - Mammals (*n = 540*)
  - Flyers & walkers from temperate areas (*n = 864*)
  - Flyers & walkers from the Northern Hemisphere (*n = 948*)
  - Passeriformes (*n = 283*)
  - Non-Passeriform flyers (*n = 494*)
  - Non-Passeriform birds (*n = 473*)
  - Artiodactyla (*n = 67*)
  - Accipitriformes (*n = 64*)
  - Pelecaniformes (*n = 29*)
  - Chiroptera (*n = 21*)
- Gaussian bayesian comparative analyses (Response variable: PC1)
  - All species (*n = 1,296*)
  - Flyers (*n = 777*)
  - Swimmers (*n = 60*)
  - Walkers (*n = 458*)
- Multinomial bayesian comparative analyses
  - Flyers (*migatory / partially migratory / non-migratory, n = 777*)

Appendix S1: Dataset

We selected mammal and bird species from the 'amniotes database' (Myhrvold et al. 2015) that had data for all seven life-history traits of interest: longevity (years), adult body mass (g), age of female sexual maturity (days), duration of prenatal development (days), duration of postnatal development (days), number of annual reproductive events and number of offspring in each reproductive event. Duration of prenatal development corresponds to gestation for mammals and incubation for birds, while duration of postnatal development corresponds to weaning for mammals and fledging age for birds.

To determine migratory strategies we tried to minimize the sources of information to avoid increasing heterogeneity due to different definitions of migration. For each locomotion group we used:

- **Flyers**: To determine the migratory strategy of birds we use the dataset from Eyre et al. (2017). For the binomial analysis we considered a bird to be migratory if it was classified as full directional migrant or partial directional migrant, the rest were considered non-migrants. For the multinomial analysis we considered birds to be migrants if they were classified as full directional migrants, to be partial migrants if they were classified as partial directional migrants and the rest of birds were classified as non-migrants. To determine the migratory strategy of bats we use several resources:

  Bisson, I. A., Safi, K., & Holland, R. A. (2009). Evidence for repeated independent evolution of migration in the largest family of bats. PLoS One, 4(10), e7504.

  Fleming, T. H., Eby, P., Kunz, T. H., & Fenton, M. B. (2003). Ecology of bat migration. Bat ecology, 156, 164-65.

  Kunz, T. H., & Fenton, M. B. (Eds.). (2005). Bat ecology. University of Chicago Press. Popa-Lisseanu, A. G., & Voigt, C. C. (2009). Bats on the move. Journal of Mammalogy, 90(6), 1283-1289.

  Taylor, P. J. (2000). Bats of Southern Africa: Guide to biology, identification, and conservation. University of Kwazulu Natal Press.

  Tidemann, C. R., & Nelson, J. E. (2004). Long-distance movements of the grey-headed flying fox (Pteropus poliocephalus). Journal of Zoology, 263(2), 141-146.

- **Swimmers**: To determine their migratory strategies we used:

  Wilson, D. E., & Mittermeier, R. A. (2014). Handbook of the mammals of the world. Vol. 4. Sea Mammals. Lynx Edicions, Barcelona.

- **Walkers**: To determine their migratory strategies we used:

  Wilson, D. E., & Mittermeier, R. A. (2009). Handbook of the mammals of the world. Vol. 1. Carnivores. Lynx Edicions, Barcelona.

  Wilson, D. E., & Mittermeier, R. A. (2011). Handbook of the mammals of the world. Vol. 2. Hoofed Mammals. Lynx Edicions, Barcelona.

  Mittermeier, R. A, Rylands, A. B., & Wilson, D. E. (2013). Handbook of the mammals of the world. Vol. 3. Primates. Lynx Edicions, Barcelona.

  Wilson, D. E., & Mittermeier, R. A. (2015). Handbook of the mammals of the world. Vol. 5. Monotremes and Marsupials. Lynx Edicions, Barcelona.

  Wilson, D. E., Lacher, T. E., & Mittermeier, R. A. (2016). Handbook of the mammals of the world. Vol. 6. Lagomorphs and Rodents I. Lynx Edicions, Barcelona.

  Wilson, D. E., Lacher, T. E., & Mittermeier, R. A. (2017). Handbook of the mammals of the world. Vol. 7. Rodents II. Lynx Edicions, Barcelona.

  Wilson, D. E., & Mittermeier, R. A. (2018). Handbook of the mammals of the world. Vol. 8. Insectivores, Sloths and Colugos. Lynx Edicions, Barcelona.

Class	Order	Non-migratory	Migratory
Bird	Accipitriformes	25	39
Bird	Anseriformes	11	48
Bird	Apodiformes	5	14
Bird	Bucerotiformes	0	1
Bird	Caprimulgiformes	0	3
Bird	Charadriiformes	19	89
Bird	Ciconiiformes	0	6
Bird	Columbiformes	4	7
Bird	Coraciiformes	2	3
Bird	Cuculiformes	0	2
Bird	Falconiformes	2	10
Bird	Galliformes	10	1
Bird	Gaviiformes	0	5
Bird	Gruiformes	3	13
Bird	Otidiformes	1	1
Bird	Passeriformes	70	213
Bird	Pelecaniformes	9	20
Bird	Phaethontiformes	1	0
Bird	Phoenicopteriformes	3	1
Bird	Piciformes	8	6
Bird	Podicipediformes	0	7
Bird	Procellariiformes	33	8
Bird	Psittaciformes	8	0
Bird	Strigiformes	24	7
Bird	Suliformes	10	4
Mammal	Artiodactyla	45	22
Mammal	Carnivora	95	9
Mammal	Cetacea	7	24
Mammal	Chiroptera	10	11
Mammal	Cingulata	1	0
Mammal	Dasyuromorphia	14	0
Mammal	Didelphimorphia	5	0
Mammal	Diprotodontia	25	0
Mammal	Erinaceomorpha	2	0
Mammal	Lagomorpha	9	1
Mammal	Macroscelidea	1	0
Mammal	Monotremata	2	0
Mammal	Peramelemorphia	5	0
Mammal	Perissodactyla	3	1
Mammal	Pilosa	3	0
Mammal	Primates	111	0
Mammal	Proboscidea	1	0
Mammal	Rodentia	109	1
Mammal	Scandentia	1	0
Mammal	Sirenia	1	2
Mammal	Soricomorpha	18	0
Mammal	Tubulidentata	1	0

Table S1: Number of non-migratory and migratory species.

Appendix S2: Phylogenetic size-correction

S2.1 Load required packages and data

Data supporting the results is archived in a data repository:

```
library(phytools)
library(nlme)
library(psych)

df <- read.csv("Dataset_LH_Migration.csv")
tree <- read.tree("mammal_bird_tree315.trees")
df$Lat <- abs(df$Lat) # absolute latitude
rownames(df) <- df$animal

matches <- match(df$animal, tree$tip.label)
tree <- drop.tip(tree, tree$tip.label[-matches]) #Subset tree for flying species

df[, c(7:13)]<-log(df[, c(7:13)]) #Log-transformation of life-history traits to
                                   #fulfill normality assumptions in posterior analyses

dfF <- subset(df, df$Loc=="F") #Subset dataset for flying species
dfS <- subset(df, df$Loc=="S") #Subset dataset for swimming species
dfW <- subset(df, df$Loc=="W") #Subset dataset for walking species
dfB <- subset(df, df$Class=="Bird") #Subset dataset for birds
dfM <- subset(df, df$Class=="Mammal") #Subset dataset for mammals

matchesF <- match(dfF$animal, tree$tip.label)
treeF <- drop.tip(tree, tree$tip.label[-matchesF]) #Subset tree for flying species

matchesS <- match(dfS$animal, tree$tip.label)
treeS <- drop.tip(tree, tree$tip.label[-matchesS]) #Subset tree for swimming species

matchesW <- match(dfW$animal, tree$tip.label)
treeW <- drop.tip(tree, tree$tip.label[-matchesW]) #Subset tree for walking species

matchesB <- match(dfB$animal, tree$tip.label)
treeB <- drop.tip(tree, tree$tip.label[-matchesB]) #Subset tree for birds

matchesM <- match(dfM$animal, tree$tip.label)
treeM <- drop.tip(tree, tree$tip.label[-matchesM]) #Subset tree for mammals
```

S2.2 MCMC models

- **All species** (n = *1,296*)

```
library(MCMCglmm)

#Size-correction for longevity
LongSC <- df[,c(2,7,10)]
LongSC_MCMC <- MCMCglmm(Long ~ mass,
                        data=LongSC, family="gaussian",
                        random=~animal, ginverse=list(animal=inverseA(tree)$Ainv),
                        prior=list(R = list(V = 1, nu=0.001),
```

```
                                  G = list(G1=list(V = 1, nu=0.001, alpha.mu=0,
                                                   alpha.V=100))),
                       pl=TRUE, nitt=5000000,thin=2500,burnin=100000, verbose =TRUE)

#Size-correction for duration of postnatal development
develSC <- df[,c(2,8,10)]
develSC_MCMC <- MCMCglmm(devel ~ mass,
                        data=develSC, family="gaussian",
                        random=~animal, ginverse=list(animal=inverseA(tree)$Ainv),
                        prior=list(R = list(V = 1, nu=0.001),
                                   G = list(G1=list(V = 1, nu=0.001, alpha.mu=0,
                                                    alpha.V=100))),
                        pl=TRUE, nitt=5000000,thin=2500,burnin=100000, verbose =TRUE)

#Size-correction for age of female sexual maturity
femSC <- df[,c(2,9,10)]
femSC_MCMC <- MCMCglmm(fem ~ mass,
                      data=femSC, family="gaussian",
                      random=~animal, ginverse=list(animal=inverseA(tree)$Ainv),
                      prior=list(R = list(V = 1, nu=0.001),
                                 G = list(G1=list(V = 1, nu=0.001, alpha.mu=0,
                                                  alpha.V=100))),
                      pl=TRUE, nitt=5000000,thin=2500,burnin=100000, verbose =TRUE)

#Size-correction for duration of prenatal development
embriSC <- df[,c(2,11,10)]
embriSC_MCMC <- MCMCglmm(embri ~ mass,
                        data=embriSC, family="gaussian",
                        random=~animal, ginverse=list(animal=inverseA(tree)$Ainv),
                        prior=list(R = list(V = 1, nu=0.001),
                                   G = list(G1=list(V = 1, nu=0.001, alpha.mu=0,
                                                    alpha.V=100))),
                        pl=TRUE, nitt=5000000,thin=2500,burnin=100000, verbose =TRUE)

#Size-correction for number of annual reproductive events
NumclSC <- df[,c(2,12,10)]
NumclSC_MCMC <- MCMCglmm(Numcl ~ mass,
                        data=NumclSC, family="gaussian",
                        random=~animal, ginverse=list(animal=inverseA(tree)$Ainv),
                        prior=list(R = list(V = 1, nu=0.001),
                                   G = list(G1=list(V = 1, nu=0.001, alpha.mu=0,
                                                    alpha.V=100))),
                        pl=TRUE, nitt=5000000,thin=2500,burnin=100000, verbose =TRUE)

#Size-correction for number of offspring in each reproductive event
SizeclSC <- df[,c(2,13,10)]
SizeclSC_MCMC <- MCMCglmm(Sizecl ~ mass,
                         data=SizeclSC, family="gaussian",
                         random=~animal, ginverse=list(animal=inverseA(tree)$Ainv),
                         prior=list(R = list(V = 1, nu=0.001),
                                    G = list(G1=list(V = 1, nu=0.001, alpha.mu=0,
                                                     alpha.V=100))),
                         pl=TRUE, nitt=5000000,thin=2500,burnin=100000, verbose =TRUE)
```

```r
df$LongR <- df$Long - predict(LongSC_MCMC, type="response")
df$develR <- df$devel - predict(develSC_MCMC, type="response")
df$femR <- df$fem - predict(femSC_MCMC, type="response")
df$embriR <- df$embri - predict(embriSC_MCMC, type="response")
df$NumclR <- df$Numcl - predict(NumclSC_MCMC, type="response")
df$SizeclR <- df$Sizecl - predict(SizeclSC_MCMC, type="response")
```

Model	Intercept	Slope	p.value	Heritability
Longevity	1.4402786	0.1773452	0.0005102	0.9548107
Postnatal development	2.8699106	0.1909735	0.0005102	0.9849714
Female sexual maturity	5.0647731	0.1680072	0.0005102	0.9954632
Prenatal development	2.9104706	0.0817138	0.0005102	0.9992125
Number reproductive events	0.6759568	-0.0786721	0.0005102	0.9802936
Number offspring	1.2564228	-0.0359540	0.0005102	0.9977350

Table S2: Estimates and heritability of the life-history MCMC regressions against mass for all species.

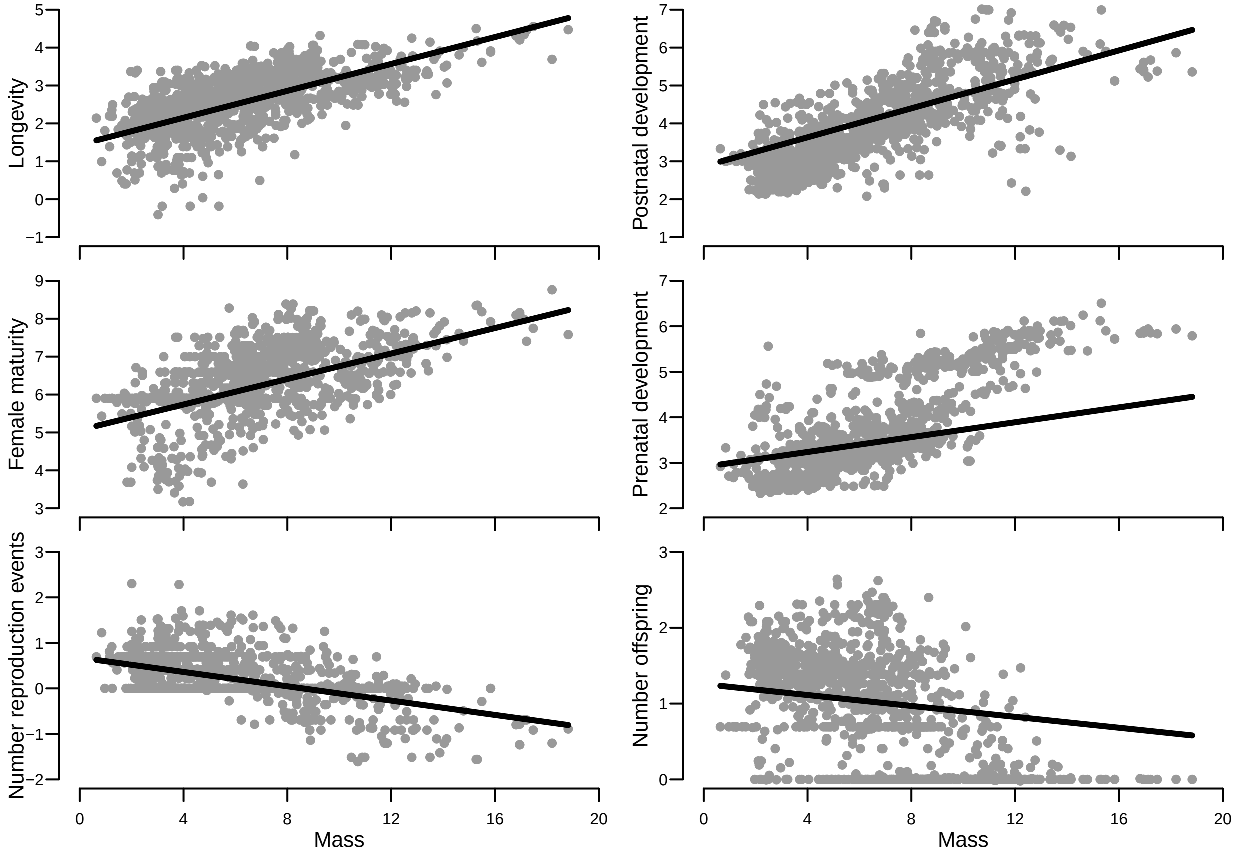

Fig S1: Life-history regressions against mass for all species. Black line corresponds to the predicted values from the MCMC models.

+ **Flyers** (n = *777*)

```r
library(MCMCglmm)

#Size-correction for longevity
FLongSC <- dfF[,c(2,7,10)]
FLongSC_MCMC <- MCMCglmm(Long ~ mass,
                         data=FLongSC, family="gaussian",
                         random=~animal, ginverse=list(animal=inverseA(tree)$Ainv),
                         prior=list(R = list(V = 1, nu=0.001),
                                    G = list(G1=list(V = 1, nu=0.001, alpha.mu=0,
                                                     alpha.V=100))),
                         pl=TRUE, nitt=5000000,thin=2500,burnin=100000, verbose =TRUE)

#Size-correction for duration of postnatal development
FdevelSC <- dfF[,c(2,8,10)]
FdevelSC_MCMC <- MCMCglmm(devel ~ mass,
                          data=FdevelSC, family="gaussian",
                          random=~animal, ginverse=list(animal=inverseA(tree)$Ainv),
                          prior=list(R = list(V = 1, nu=0.001),
                                     G = list(G1=list(V = 1, nu=0.001, alpha.mu=0,
                                                      alpha.V=100))),
                          pl=TRUE, nitt=5000000,thin=2500,burnin=100000, verbose =TRUE)

#Size-correction for age of female sexual maturity
FfemSC <- dfF[,c(2,9,10)]
FfemSC_MCMC <- MCMCglmm(fem ~ mass,
                        data=FfemSC, family="gaussian",
                        random=~animal, ginverse=list(animal=inverseA(tree)$Ainv),
                        prior=list(R = list(V = 1, nu=0.001),
                                   G = list(G1=list(V = 1, nu=0.001, alpha.mu=0,
                                                    alpha.V=100))),
                        pl=TRUE, nitt=5000000,thin=2500,burnin=100000, verbose =TRUE)

#Size-correction for duration of prenatal development
FembriSC <- dfF[,c(2,11,10)]
FembriSC_MCMC <- MCMCglmm(embri ~ mass,
                          data=FembriSC, family="gaussian",
                          random=~animal, ginverse=list(animal=inverseA(tree)$Ainv),
                          prior=list(R = list(V = 1, nu=0.001),
                                     G = list(G1=list(V = 1, nu=0.001, alpha.mu=0,
                                                      alpha.V=100))),
                          pl=TRUE, nitt=5000000,thin=2500,burnin=100000, verbose =TRUE)

#Size-correction for number of annual reproductive events
FNumclSC <- dfF[,c(2,12,10)]
FNumclSC_MCMC <- MCMCglmm(Numcl ~ mass,
                          data=FNumclSC, family="gaussian",
                          random=~animal, ginverse=list(animal=inverseA(tree)$Ainv),
                          prior=list(R = list(V = 1, nu=0.001),
                                     G = list(G1=list(V = 1, nu=0.001, alpha.mu=0,
                                                      alpha.V=100))),
                          pl=TRUE, nitt=5000000,thin=2500,burnin=100000, verbose =TRUE)

#Size-correction for number of offspring in each reproductive event
```

```r
FSizeclSC <- dfF[,c(2,13,10)]
FSizeclSC_MCMC <- MCMCglmm(Sizecl ~ mass,
                           data=FSizeclSC, family="gaussian",
                           random=~animal, ginverse=list(animal=inverseA(tree)$Ainv),
                           prior=list(R = list(V = 1, nu=0.001),
                                      G = list(G1=list(V = 1, nu=0.001, alpha.mu=0,
                                                       alpha.V=100))),
                           pl=TRUE, nitt=5000000,thin=2500,burnin=100000, verbose =TRUE)
```

```r
dfF$LongR <- dfF$Long - predict(FLongSC_MCMC, type="response")
dfF$develR <- dfF$devel - predict(FdevelSC_MCMC, type="response")
dfF$femR <- dfF$fem - predict(FfemSC_MCMC, type="response")
dfF$embriR <- dfF$embri - predict(FembriSC_MCMC, type="response")
dfF$NumclR <- dfF$Numcl - predict(FNumclSC_MCMC, type="response")
dfF$SizeclR <- dfF$Sizecl - predict(FSizeclSC_MCMC, type="response")
```

Model	Intercept	Slope	p.value	Heritability
Longevity	1.6009807	0.2209633	0.0005102	0.9344561
Postnatal development	2.7504321	0.2138863	0.0005102	0.9804555
Female sexual maturity	5.3264730	0.1748149	0.0005102	0.9932101
Prenatal development	3.5039551	0.0895044	0.0005102	0.9962410
Number reproductive events	0.2871894	-0.0409672	0.0005102	0.9646480
Number offspring	0.9413781	-0.0336107	0.0102041	0.9987482

Table S3: Estimates and heritability of the life-history MCMC regressions against mass for flyers.

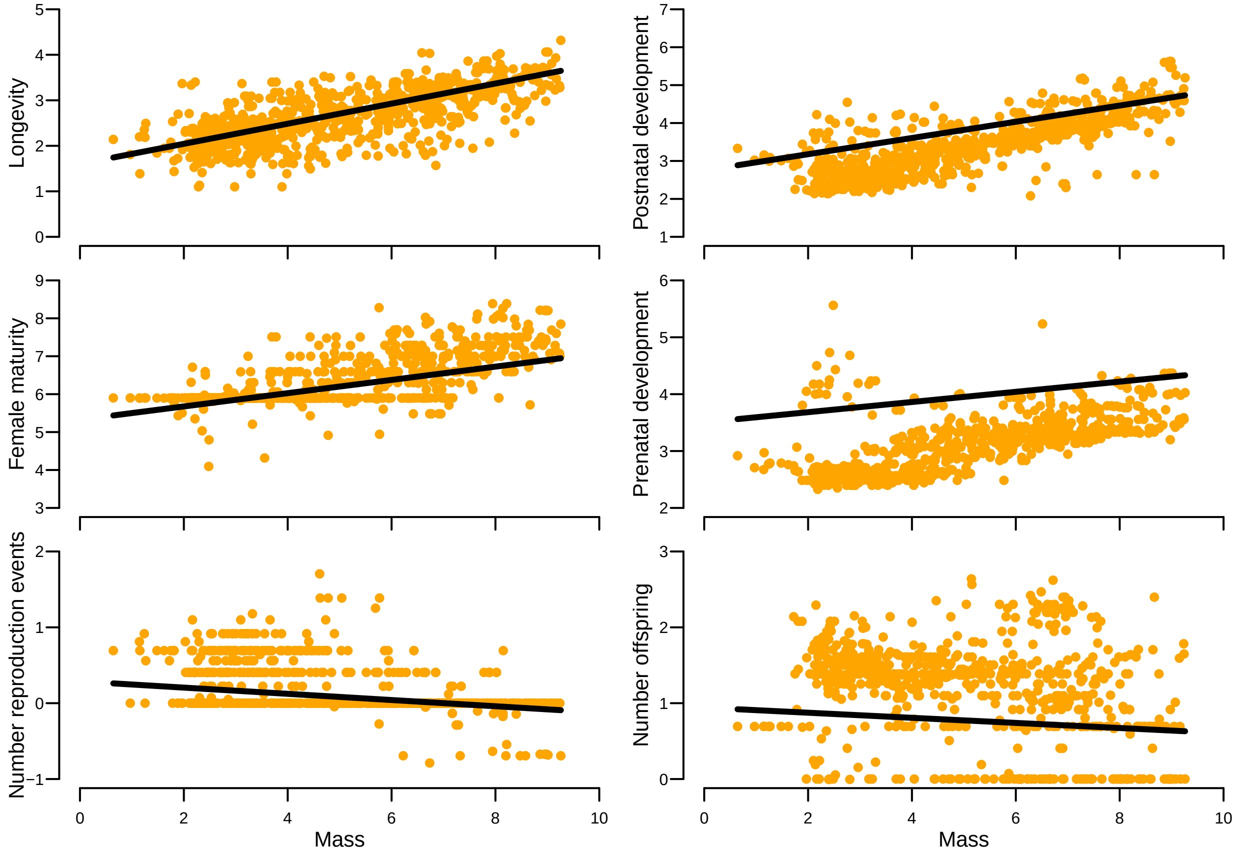

Fig S2: Life-history regressions against mass for flyers. Black line corresponds to the predicted values from the MCMC models.

- **Swimmers** (n = *60*)

```
library(MCMCglmm)

#Size-correction for longevity
SLongSC <- dfS[,c(2,7,10)]
SLongSC_MCMC <- MCMCglmm(Long ~ mass,
                         data=SLongSC, family="gaussian",
                         random=~animal, ginverse=list(animal=inverseA(tree)$Ainv),
                         prior=list(R = list(V = 1, nu=0.001),
                                    G = list(G1=list(V = 1, nu=0.001, alpha.mu=0,
                                                     alpha.V=100))),
                         pl=TRUE, nitt=5000000,thin=2500,burnin=100000, verbose =TRUE)

#Size-correction for duration of postnatal development
SdevelSC <- dfS[,c(2,8,10)]
SdevelSC_MCMC <- MCMCglmm(devel ~ mass,
                          data=SdevelSC, family="gaussian",
                          random=~animal, ginverse=list(animal=inverseA(tree)$Ainv),
                          prior=list(R = list(V = 1, nu=0.001),
                                     G = list(G1=list(V = 1, nu=0.001, alpha.mu=0,
                                                      alpha.V=100))),
                          pl=TRUE, nitt=5000000,thin=2500,burnin=100000, verbose =TRUE)
```

```r
#Size-correction for age of female sexual maturity
SfemSC <- dfS[,c(2,9,10)]
SfemSC_MCMC <- MCMCglmm(fem ~ mass,
                        data=SfemSC, family="gaussian",
                        random=~animal, ginverse=list(animal=inverseA(tree)$Ainv),
                        prior=list(R = list(V = 1, nu=0.001),
                                   G = list(G1=list(V = 1, nu=0.001, alpha.mu=0,
                                                    alpha.V=100))),
                        pl=TRUE, nitt=5000000,thin=2500,burnin=100000, verbose =TRUE)

#Size-correction for duration of prenatal development
SembriSC <- dfS[,c(2,11,10)]
SembriSC_MCMC <- MCMCglmm(embri ~ mass,
                          data=SembriSC, family="gaussian",
                          random=~animal, ginverse=list(animal=inverseA(tree)$Ainv),
                          prior=list(R = list(V = 1, nu=0.001),
                                     G = list(G1=list(V = 1, nu=0.001, alpha.mu=0,
                                                      alpha.V=100))),
                          pl=TRUE, nitt=5000000,thin=2500,burnin=100000, verbose =TRUE)

#Size-correction for number of annual reproductive events
SNumclSC <- dfS[,c(2,12,10)]
SNumclSC_MCMC <- MCMCglmm(Numcl ~ mass,
                          data=SNumclSC, family="gaussian",
                          random=~animal, ginverse=list(animal=inverseA(tree)$Ainv),
                          prior=list(R = list(V = 1, nu=0.001),
                                     G = list(G1=list(V = 1, nu=0.001, alpha.mu=0,
                                                      alpha.V=100))),
                          pl=TRUE, nitt=5000000,thin=2500,burnin=100000, verbose =TRUE)

#Size-correction for number of offspring in each reproductive event
SSizeclSC <- dfS[,c(2,13,10)]
SSizeclSC_MCMC <- MCMCglmm(Sizecl ~ mass,
                           data=SSizeclSC, family="gaussian",
                           random=~animal, ginverse=list(animal=inverseA(tree)$Ainv),
                           prior=list(R = list(V = 1, nu=0.001),
                                      G = list(G1=list(V = 1, nu=0.001, alpha.mu=0,
                                                       alpha.V=100))),
                           pl=TRUE, nitt=5000000,thin=2500,burnin=100000, verbose =TRUE)
```

```r
dfS$LongR <- dfS$Long - predict(SLongSC_MCMC, type="response")
dfS$develR <- dfS$devel - predict(SdevelSC_MCMC, type="response")
dfS$femR <- dfS$fem - predict(SfemSC_MCMC, type="response")
dfS$embriR <- dfS$embri - predict(SembriSC_MCMC, type="response")
dfS$NumclR <- dfS$Numcl - predict(SNumclSC_MCMC, type="response")
dfS$SizeclR <- dfS$Sizecl - predict(SSizeclSC_MCMC, type="response")
```

Model	Intercept	Slope	p.value	Heritability
Longevity	1.7469554	0.1429250	0.0020408	0.9514140
Postnatal development	5.1756135	0.0381851	0.5704082	0.9933779
Female sexual maturity	6.3816867	0.0912887	0.0071429	0.9100369
Prenatal development	5.2184905	0.0443397	0.0010204	0.8518330
Number reproductive events	0.4816122	-0.0865028	0.0091837	0.9914263
Number offspring	0.0627174	-0.0037069	0.4459184	0.2395821

Table S4: Estimates and heritability of the life-history MCMC regressions against mass for swimmers.

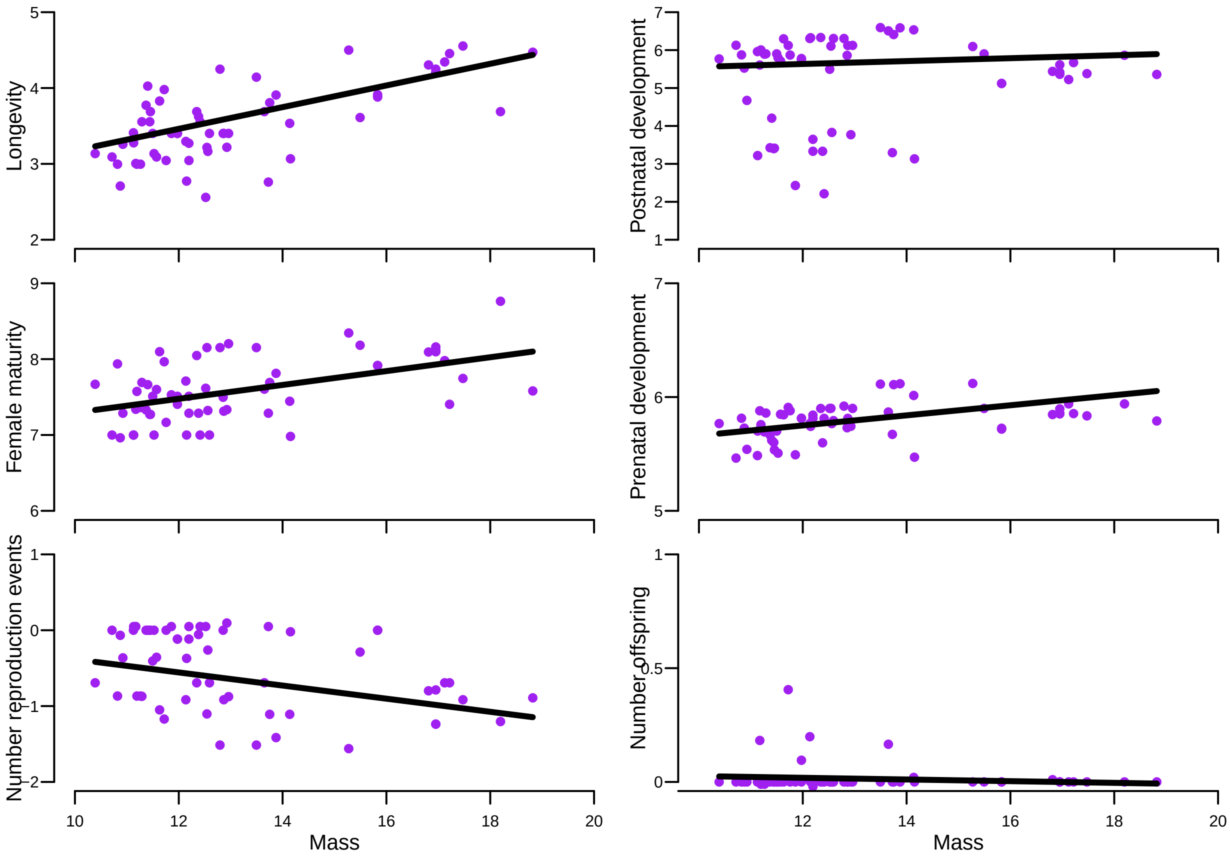

Fig S3: Life-history regressions against mass for swimmers. Black line corresponds to the predicted values from the MCMC models.

- **Walkers** ($n = 458$)

```
library(MCMCglmm)

#Size-correction for longevity
WLongSC <- dfW[,c(2,7,10)]
WLongSC_MCMC <- MCMCglmm(Long ~ mass,
                         data=WLongSC, family="gaussian",
                         random=~animal, ginverse=list(animal=inverseA(tree)$Ainv),
                         prior=list(R = list(V = 1, nu=0.001),
                                    G = list(G1=list(V = 1, nu=0.001, alpha.mu=0,
                                                     alpha.V=100))),
                         pl=TRUE, nitt=5000000,thin=2500,burnin=100000, verbose =TRUE)
```

```r
#Size-correction for duration of postnatal development
WdevelSC <- dfW[,c(2,8,10)]
WdevelSC_MCMC <- MCMCglmm(devel ~ mass,
                          data=WdevelSC, family="gaussian",
                          random=~animal, ginverse=list(animal=inverseA(tree)$Ainv),
                          prior=list(R = list(V = 1, nu=0.001),
                                     G = list(G1=list(V = 1, nu=0.001, alpha.mu=0,
                                                      alpha.V=100))),
                          pl=TRUE, nitt=5000000,thin=2500,burnin=100000, verbose =TRUE)

#Size-correction for age of female sexual maturity
WfemSC <- dfW[,c(2,9,10)]
WfemSC_MCMC <- MCMCglmm(fem ~ mass,
                        data=WfemSC, family="gaussian",
                        random=~animal, ginverse=list(animal=inverseA(tree)$Ainv),
                        prior=list(R = list(V = 1, nu=0.001),
                                   G = list(G1=list(V = 1, nu=0.001, alpha.mu=0,
                                                    alpha.V=100))),
                        pl=TRUE, nitt=5000000,thin=2500,burnin=100000, verbose =TRUE)

#Size-correction for duration of prenatal development
WembriSC <- dfW[,c(2,11,10)]
WembriSC_MCMC <- MCMCglmm(embri ~ mass,
                          data=WembriSC, family="gaussian",
                          random=~animal, ginverse=list(animal=inverseA(tree)$Ainv),
                          prior=list(R = list(V = 1, nu=0.001),
                                     G = list(G1=list(V = 1, nu=0.001, alpha.mu=0,
                                                      alpha.V=100))),
                          pl=TRUE, nitt=5000000,thin=2500,burnin=100000, verbose =TRUE)

#Size-correction for number of annual reproductive events
WNumclSC <- dfW[,c(2,12,10)]
WNumclSC_MCMC <- MCMCglmm(Numcl ~ mass,
                          data=WNumclSC, family="gaussian",
                          random=~animal, ginverse=list(animal=inverseA(tree)$Ainv),
                          prior=list(R = list(V = 1, nu=0.001),
                                     G = list(G1=list(V = 1, nu=0.001, alpha.mu=0,
                                                      alpha.V=100))),
                          pl=TRUE, nitt=5000000,thin=2500,burnin=100000, verbose =TRUE)

#Size-correction for number of offspring in each reproductive event
WSizeclSC <- dfW[,c(2,13,10)]
WSizeclSC_MCMC <- MCMCglmm(Sizecl ~ mass,
                           data=WSizeclSC, family="gaussian",
                           random=~animal, ginverse=list(animal=inverseA(tree)$Ainv),
                           prior=list(R = list(V = 1, nu=0.001),
                                      G = list(G1=list(V = 1, nu=0.001, alpha.mu=0,
                                                       alpha.V=100))),
                           pl=TRUE, nitt=5000000,thin=2500,burnin=100000, verbose =TRUE)
```

```r
dfW$LongR <- dfW$Long - predict(WLongSC_MCMC, type="response")
dfW$develR <- dfW$devel - predict(WdevelSC_MCMC, type="response")
dfW$femR <- dfW$fem - predict(WfemSC_MCMC, type="response")
```

```
dfW$embriR <- dfW$embri - predict(WembriSC_MCMC, type="response")
dfW$NumclR <- dfW$Numcl - predict(WNumclSC_MCMC, type="response")
dfW$SizeclR <- dfW$Sizecl - predict(WSizeclSC_MCMC, type="response")
```

Model	Intercept	Slope	p.value	Heritability
Longevity	1.3878969	0.1609915	0.0005102	0.9750963
Postnatal development	3.0845021	0.2049633	0.0005102	0.9721041
Female sexual maturity	4.7596330	0.1698563	0.0005102	0.9963108
Prenatal development	3.0637797	0.0748886	0.0005102	0.9990592
Number reproductive events	0.9272442	-0.1024459	0.0005102	0.9848308
Number offspring	1.0799348	-0.0479663	0.0005102	0.9859969

Table S5: Estimates and heritability of the life-history MCMC regressions against mass for walkers.

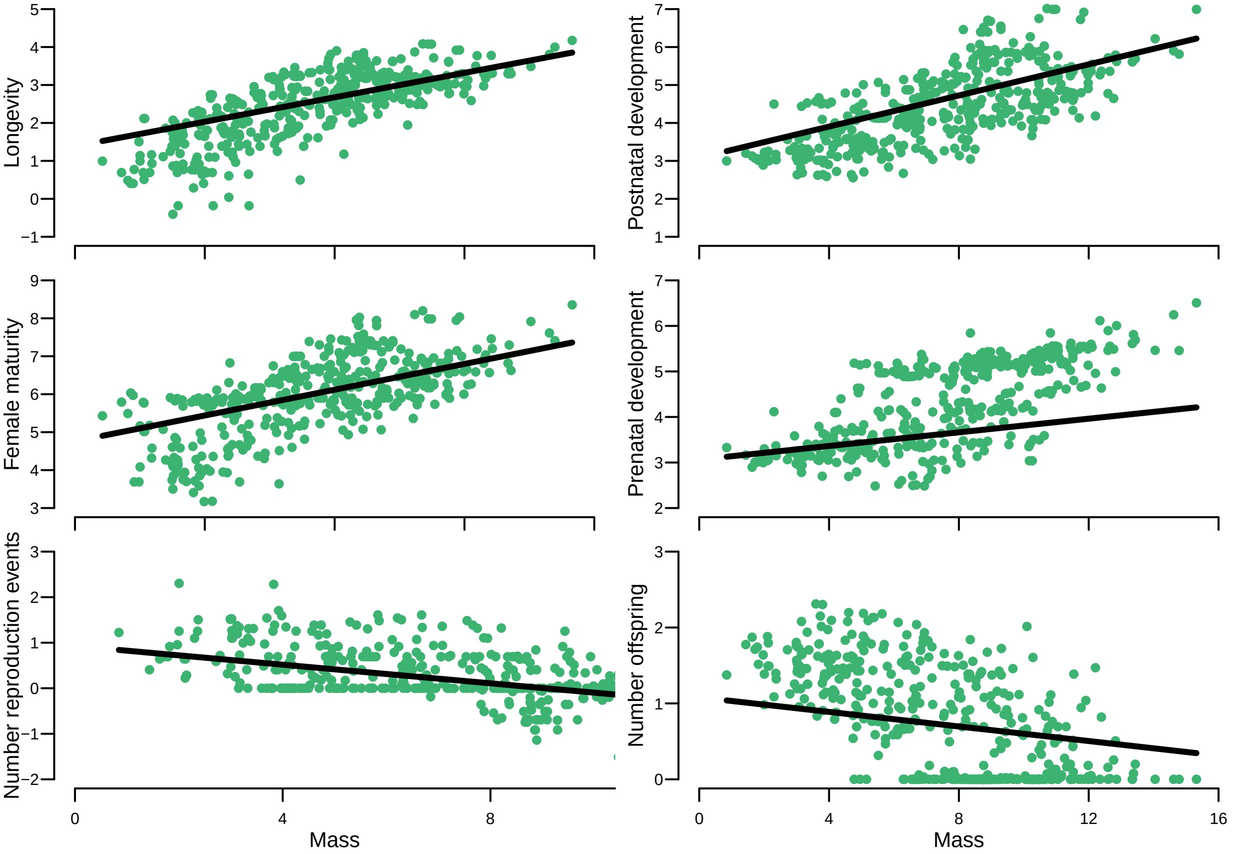

Fig S4: Life-history regressions against mass for walkers. Black line corresponds to the predicted values from the MCMC models.

- **Birds**

```
library(MCMCglmm)

#Size-correction for longevity
BLongSC <- dfB[,c(2,7,10)]
```

```r
BLongSC_MCMC <- MCMCglmm(Long ~ mass,
                         data=BLongSC, family="gaussian",
                         random=~animal, ginverse=list(animal=inverseA(tree)$Ainv),
                         prior=list(R = list(V = 1, nu=0.001),
                                    G = list(G1=list(V = 1, nu=0.001, alpha.mu=0,
                                                     alpha.V=100))),
                         pl=TRUE, nitt=5000000,thin=2500,burnin=100000, verbose =TRUE)

#Size-correction for duration of postnatal development
BdevelSC <- dfB[,c(2,8,10)]
BdevelSC_MCMC <- MCMCglmm(devel ~ mass,
                          data=BdevelSC, family="gaussian",
                          random=~animal, ginverse=list(animal=inverseA(tree)$Ainv),
                          prior=list(R = list(V = 1, nu=0.001),
                                     G = list(G1=list(V = 1, nu=0.001, alpha.mu=0,
                                                      alpha.V=100))),
                          pl=TRUE, nitt=5000000,thin=2500,burnin=100000, verbose =TRUE)

#Size-correction for age of female sexual maturity
BfemSC <- dfB[,c(2,9,10)]
BfemSC_MCMC <- MCMCglmm(fem ~ mass,
                        data=BfemSC, family="gaussian",
                        random=~animal, ginverse=list(animal=inverseA(tree)$Ainv),
                        prior=list(R = list(V = 1, nu=0.001),
                                   G = list(G1=list(V = 1, nu=0.001, alpha.mu=0,
                                                    alpha.V=100))),
                        pl=TRUE, nitt=5000000,thin=2500,burnin=100000, verbose =TRUE)

#Size-correction for duration of prenatal development
BembriSC <- dfB[,c(2,11,10)]
BembriSC_MCMC <- MCMCglmm(embri ~ mass,
                          data=BembriSC, family="gaussian",
                          random=~animal, ginverse=list(animal=inverseA(tree)$Ainv),
                          prior=list(R = list(V = 1, nu=0.001),
                                     G = list(G1=list(V = 1, nu=0.001, alpha.mu=0,
                                                      alpha.V=100))),
                          pl=TRUE, nitt=5000000,thin=2500,burnin=100000, verbose =TRUE)

#Size-correction for number of annual reproductive events
BNumclSC <- dfB[,c(2,12,10)]
BNumclSC_MCMC <- MCMCglmm(Numcl ~ mass,
                          data=BNumclSC, family="gaussian",
                          random=~animal, ginverse=list(animal=inverseA(tree)$Ainv),
                          prior=list(R = list(V = 1, nu=0.001),
                                     G = list(G1=list(V = 1, nu=0.001, alpha.mu=0,
                                                      alpha.V=100))),
                          pl=TRUE, nitt=5000000,thin=2500,burnin=100000, verbose =TRUE)

#Size-correction for number of offspring in each reproductive event
BSizeclSC <- dfB[,c(2,13,10)]
BSizeclSC_MCMC <- MCMCglmm(Sizecl ~ mass,
                           data=BSizeclSC, family="gaussian",
                           random=~animal, ginverse=list(animal=inverseA(tree)$Ainv),
```

```r
                          prior=list(R = list(V = 1, nu=0.001),
                                     G = list(G1=list(V = 1, nu=0.001, alpha.mu=0,
                                                      alpha.V=100))),
                          pl=TRUE, nitt=5000000,thin=2500,burnin=100000, verbose =TRUE)
```

```r
dfB$LongR <- dfB$Long - predict(BLongSC_MCMC, type="response")
dfB$develR <- dfB$devel - predict(BdevelSC_MCMC, type="response")
dfB$femR <- dfB$fem - predict(BfemSC_MCMC, type="response")
dfB$embriR <- dfB$embri - predict(BembriSC_MCMC, type="response")
dfB$NumclR <- dfB$Numcl - predict(BNumclSC_MCMC, type="response")
dfB$SizeclR <- dfB$Sizecl - predict(BSizeclSC_MCMC, type="response")
```

Model	Intercept	Slope	p.value	Heritability
Longevity	1.3187788	0.2248475	0.0005102	0.9368375
Postnatal development	2.3370589	0.2129033	0.0005102	0.9816289
Female sexual maturity	5.3332035	0.1774904	0.0005102	0.9935191
Prenatal development	2.6919865	0.0877299	0.0005102	0.9954374
Number reproductive events	0.3973528	-0.0418971	0.0005102	0.9674073
Number offspring	1.5990940	-0.0342671	0.0091837	0.9987859

Table S6: Estimates and heritability of the life-history MCMC regressions against mass for birds.

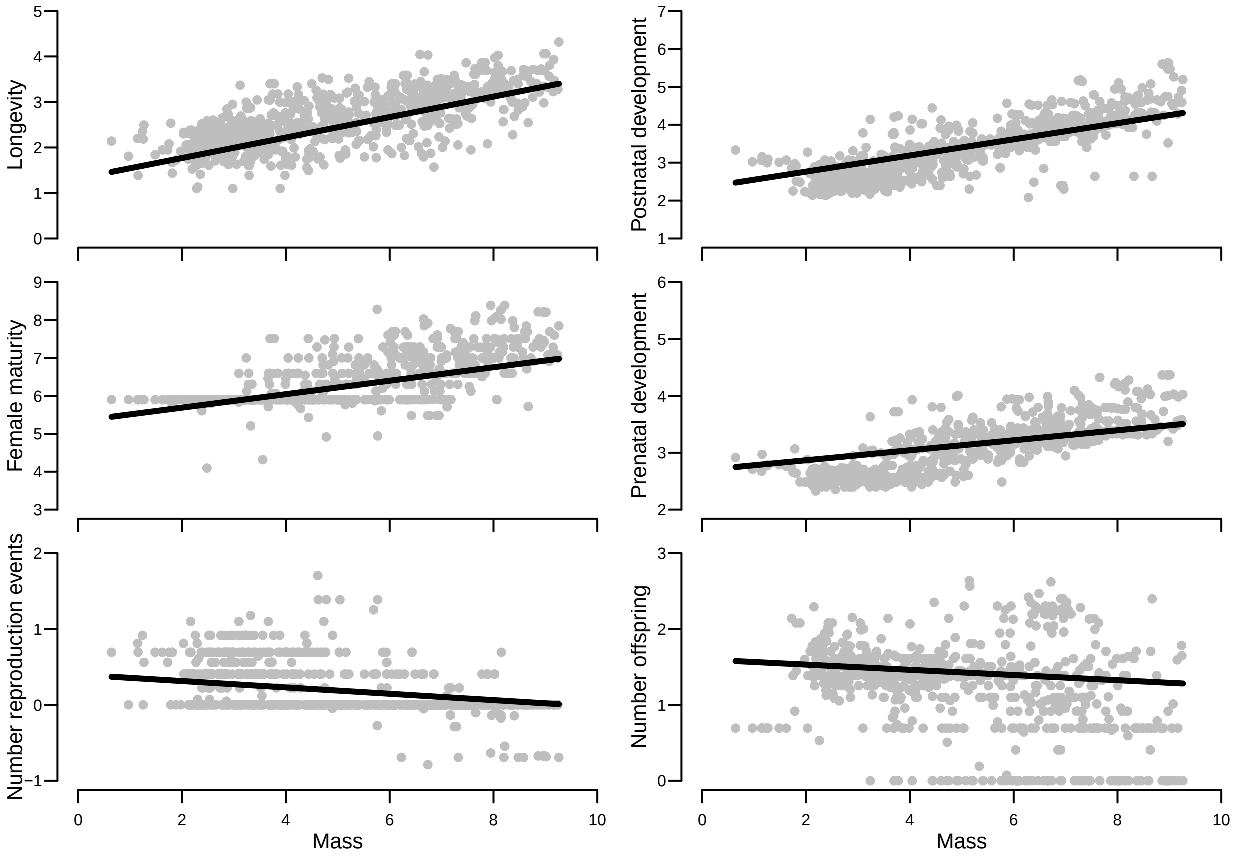

Fig S5: Life-history regressions against mass for birds. Black line corresponds to the predicted values from the MCMC models.

- **Mammals**

```
library(MCMCglmm)

#Size-correction for longevity
MLongSC <- dfM[,c(2,7,10)]
MLongSC_MCMC <- MCMCglmm(Long ~ mass,
                         data=MLongSC, family="gaussian",
                         random=~animal, ginverse=list(animal=inverseA(tree)$Ainv),
                         prior=list(R = list(V = 1, nu=0.001),
                                    G = list(G1=list(V = 1, nu=0.001, alpha.mu=0,
                                                     alpha.V=100))),
                         pl=TRUE, nitt=5000000,thin=2500,burnin=100000, verbose =TRUE)

#Size-correction for duration of postnatal development
MdevelSC <- dfM[,c(2,8,10)]
MdevelSC_MCMC <- MCMCglmm(devel ~ mass,
                          data=MdevelSC, family="gaussian",
                          random=~animal, ginverse=list(animal=inverseA(tree)$Ainv),
                          prior=list(R = list(V = 1, nu=0.001),
                                     G = list(G1=list(V = 1, nu=0.001, alpha.mu=0,
                                                      alpha.V=100))),
                          pl=TRUE, nitt=5000000,thin=2500,burnin=100000, verbose =TRUE)

#Size-correction for age of female sexual maturity
MfemSC <- dfM[,c(2,9,10)]
MfemSC_MCMC <- MCMCglmm(fem ~ mass,
                        data=MfemSC, family="gaussian",
                        random=~animal, ginverse=list(animal=inverseA(tree)$Ainv),
                        prior=list(R = list(V = 1, nu=0.001),
                                   G = list(G1=list(V = 1, nu=0.001, alpha.mu=0,
                                                    alpha.V=100))),
                        pl=TRUE, nitt=5000000,thin=2500,burnin=100000, verbose =TRUE)

#Size-correction for duration of prenatal development
MembriSC <- dfM[,c(2,11,10)]
MembriSC_MCMC <- MCMCglmm(embri ~ mass,
                          data=MembriSC, family="gaussian",
                          random=~animal, ginverse=list(animal=inverseA(tree)$Ainv),
                          prior=list(R = list(V = 1, nu=0.001),
                                     G = list(G1=list(V = 1, nu=0.001, alpha.mu=0,
                                                      alpha.V=100))),
                          pl=TRUE, nitt=5000000,thin=2500,burnin=100000, verbose =TRUE)

#Size-correction for number of annual reproductive events
MNumclSC <- dfM[,c(2,12,10)]
MNumclSC_MCMC <- MCMCglmm(Numcl ~ mass,
                          data=MNumclSC, family="gaussian",
                          random=~animal, ginverse=list(animal=inverseA(tree)$Ainv),
                          prior=list(R = list(V = 1, nu=0.001),
                                     G = list(G1=list(V = 1, nu=0.001, alpha.mu=0,
                                                      alpha.V=100))),
                          pl=TRUE, nitt=5000000,thin=2500,burnin=100000, verbose =TRUE)
```

```r
#Size-correction for number of offspring in each reproductive event
MSizeclSC <- dfM[,c(2,13,10)]
MSizeclSC_MCMC <- MCMCglmm(Sizecl ~ mass,
                           data=MSizeclSC, family="gaussian",
                           random=~animal, ginverse=list(animal=inverseA(tree)$Ainv),
                           prior=list(R = list(V = 1, nu=0.001),
                                      G = list(G1=list(V = 1, nu=0.001, alpha.mu=0,
                                                       alpha.V=100))),
                           pl=TRUE, nitt=5000000,thin=2500,burnin=100000, verbose =TRUE)
```

```r
dfM$LongR <- dfM$Long - predict(MLongSC_MCMC, type="response")
dfM$develR <- dfM$devel - predict(MdevelSC_MCMC, type="response")
dfM$femR <- dfM$fem - predict(MfemSC_MCMC, type="response")
dfM$embriR <- dfM$embri - predict(MembriSC_MCMC, type="response")
dfM$NumclR <- dfM$Numcl - predict(MNumclSC_MCMC, type="response")
dfM$SizeclR <- dfM$Sizecl - predict(MSizeclSC_MCMC, type="response")
```

Model	Intercept	Slope	p.value	Heritability
Longevity	1.5008831	0.1531915	0.0005102	0.9751090
Postnatal development	3.2964023	0.1821508	0.0005102	0.9793649
Female sexual maturity	4.8511106	0.1653131	0.0005102	0.9936043
Prenatal development	3.1176410	0.0785993	0.0005102	0.9990168
Number reproductive events	0.8858039	-0.0982391	0.0005102	0.9855343
Number offspring	0.9958150	-0.0418351	0.0005102	0.9882991

Table S7: Estimates and heritability of the life-history MCMC regressions against mass for mammals.

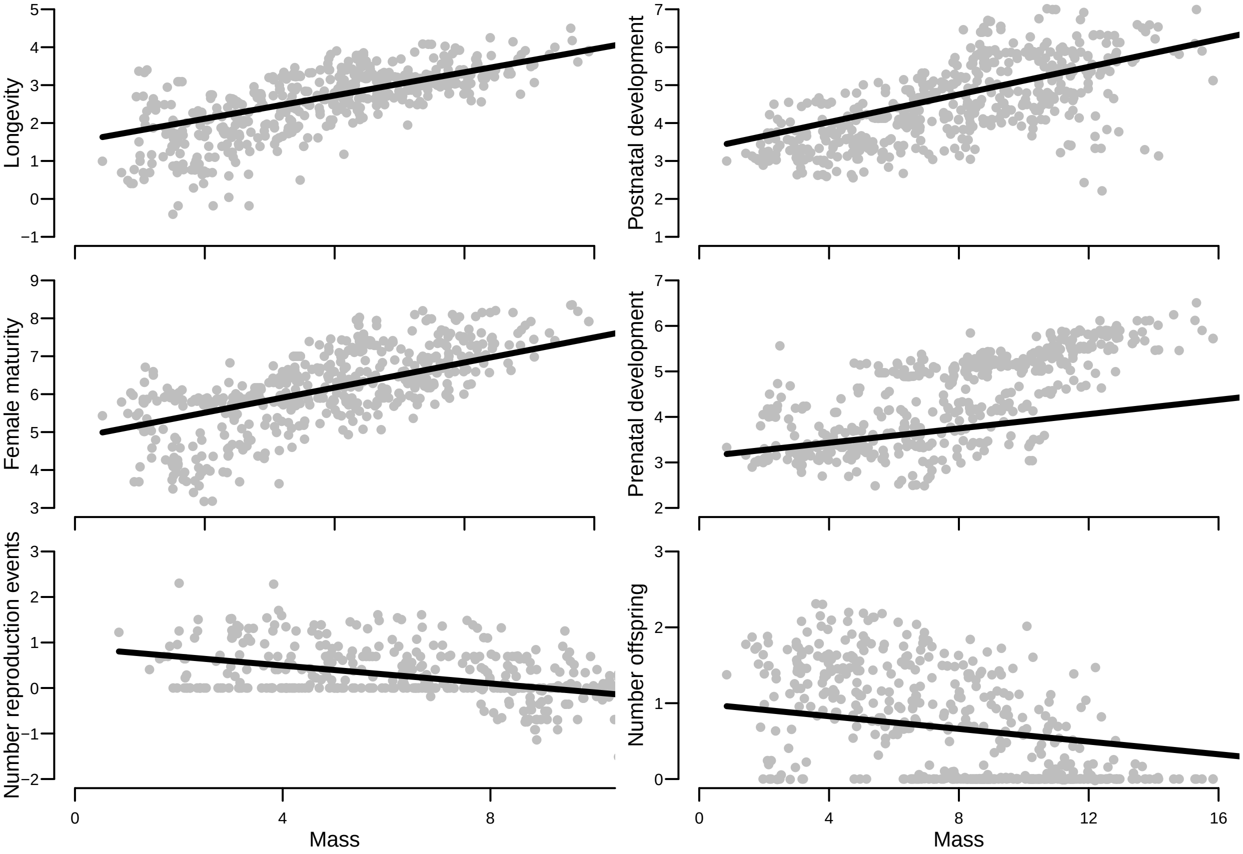

Fig S6: Life-history regressions against mass for mammals. Black line corresponds to the predicted values from the MCMC models.

Appendix S3: Phylogenetic principal components analysis

S3.1 All species PCA

```
full_mat <- as.matrix(df[,c(17:22)]) # life-history traits selected
rownames(full_mat) <- df$animal

# Bartlett test
# To estimate collinearity between the variables and check appropriatnes of PCA.
# Null hypothesis rejected indicates the possibility of using PCA

full_matCor <- cor(full_mat)
cortest.bartlett(full_matCor, n = 1296)
```

```
## $chisq
## [1] 2733.954
##
## $p.value
## [1] 0
##
```

```
## $df
## [1] 15
```

```
PCA_full <- phyl.pca(tree, full_mat, method="lambda", mode="corr") #PCA
```

	PC1	PC2	PC3	PC4	PC5	PC6
Standard deviation	1.3115391	1.0238512	0.9955221	0.9273165	0.8482906	0.8130294
Proportion of Variance	0.2866892	0.1747119	0.1651774	0.1433193	0.1199328	0.1101695
Cumulative Proportion	0.2866892	0.4614010	0.6265784	0.7698977	0.8898305	1.0000000

Table S8: Importance of components of the PCA for all species.

	PC1	PC2	PC3	PC4	PC5	PC6
PC1	1.720135	0.000000	0.0000000	0.0000000	0.0000000	0.0000000
PC2	0.000000	1.048271	0.0000000	0.0000000	0.0000000	0.0000000
PC3	0.000000	0.000000	0.9910642	0.0000000	0.0000000	0.0000000
PC4	0.000000	0.000000	0.0000000	0.8599159	0.0000000	0.0000000
PC5	0.000000	0.000000	0.0000000	0.0000000	0.7195969	0.0000000
PC6	0.000000	0.000000	0.0000000	0.0000000	0.0000000	0.6610168

Table S9: Eigenvalues of the PCA for all species.

	PC1	PC2	PC3	PC4	PC5	PC6
LongR	-0.1485632	0.0754150	0.9721974	0.0598342	0.1125968	0.1039982
develR	-0.4473784	-0.0003027	-0.0522867	0.7865432	-0.2282553	-0.3554826
femR	-0.4718318	0.2743629	0.0200286	-0.4843398	-0.6820998	-0.0430519
embriR	-0.5348219	-0.2487557	-0.1681594	0.0787876	0.1604575	0.7693208
NumclR	0.2787705	-0.7729503	0.1529605	0.0500286	-0.5401280	0.0848339
SizeclR	0.4375029	0.5096135	-0.0047662	0.3667073	-0.3904824	0.5117732

Table S10: Eigenvectors of the PCA for all species.

S3.2 Flyers PCA

```
F_mat <- as.matrix(dfF[,c(17:22)]) # life-history traits selected
rownames(F_mat) <- dfF$animal
PCA_F <- phyl.pca(treeF, F_mat, method="lambda",mode="corr") #PCA
```

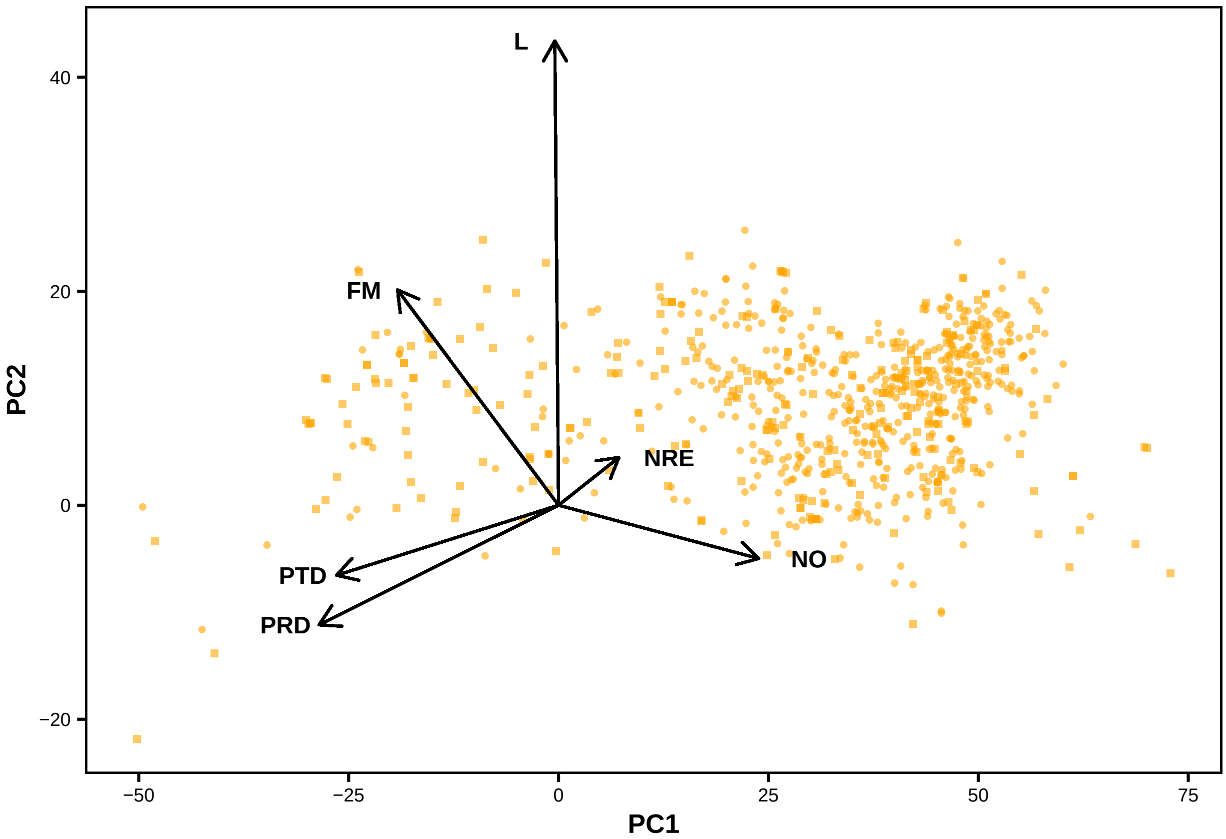

Fig S7: Phylogenetically corrected PCA of life-history traits for flyers. Arrow length indicates the loading of each life-history trait onto PCA axes. Squares represent the position of non-migratory species along the PCA axes and circles of migratory species.

	PC1	PC2	PC3	PC4	PC5	PC6
Standard deviation	1.3071578	1.0234027	1.0032838	0.9405812	0.8788589	0.7617881
Proportion of Variance	0.2847769	0.1745588	0.1677631	0.1474488	0.1287322	0.0967202
Cumulative Proportion	0.2847769	0.4593358	0.6270988	0.7745477	0.9032798	1.0000000

Table S11: Importance of components of the PCA for flyers.

	PC1	PC2	PC3	PC4	PC5	PC6
LongR	-0.0088961	0.8676886	-0.0591969	0.4445739	0.1813584	-0.1140014
develR	-0.5288908	-0.1310049	-0.0689269	0.5067589	-0.2872538	0.5992011
femR	-0.3835701	0.4025571	0.2412400	-0.5352453	-0.5861105	-0.0511080
embriR	-0.5703298	-0.2235788	-0.1985988	0.2021568	0.0176489	-0.7376422
NumclR	0.1436230	0.0893356	-0.9319806	-0.1729673	-0.2634280	0.0590916
SizeclR	0.4766190	-0.0996682	0.1597461	0.4339088	-0.6865589	-0.2788223

Table S12: Eigenvectors of the PCA for flyers.

S3.3 Swimmers PCA

```
S_mat <- as.matrix(dfS[,c(17:22)]) # life-history traits selected
rownames(S_mat) <- dfS$animal
PCA_S <- phyl.pca(treeS, S_mat, method="lambda",mode="corr") #PCA
```

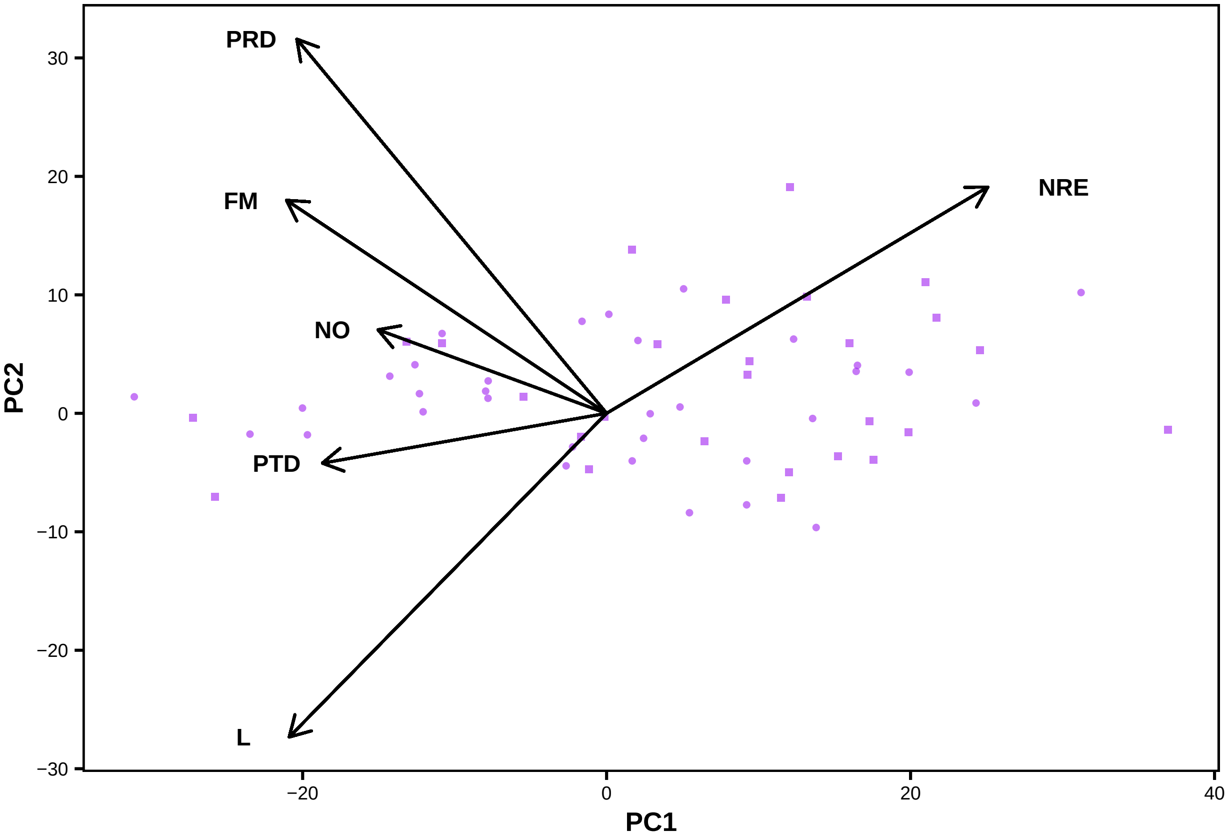

Fig S8: Phylogenetically corrected PCA of life-history traits for swimmers. Arrow length indicates the loading of each life-history trait onto PCA axes. Squares represent the position of non-migratory species along the PCA axes and circles of migratory species.

	PC1	PC2	PC3	PC4	PC5	PC6
Standard deviation	1.4219461	1.0107195	0.9629238	0.9492456	0.7780432	0.7231007
Proportion of Variance	0.3369885	0.1702590	0.1545370	0.1501779	0.1008919	0.0871458
Cumulative Proportion	0.3369885	0.5072475	0.6617845	0.8119624	0.9128542	1.0000000

Table S13: Importance of components of the PCA for swimmers.

	PC1	PC2	PC3	PC4	PC5	PC6
LongR	-0.4176805	-0.5465362	-0.2931781	-0.2903967	0.0001156	0.5971244
develR	-0.3740076	-0.0841683	0.5834650	0.5445777	0.4133357	0.2125825
femR	-0.4211332	0.3595960	-0.0012989	-0.5649478	0.5622432	-0.2409408
embriR	-0.4076479	0.6317362	0.0928963	0.0066120	-0.5559600	0.3420053
NumclR	0.5016536	0.3819969	-0.1732569	0.0233850	0.4231201	0.6267588
SizeclR	-0.3007908	0.1410399	-0.7314136	0.5471274	0.1578418	-0.1743686

Table S14: Eigenvectors of the PCA for swimmers.

S3.4 Walkers PCA

```
W_mat <- as.matrix(dfW[,c(17:22)]) # life-history traits selected
rownames(W_mat) <- dfW$animal
PCA_W <- phyl.pca(treeW, W_mat, method="lambda",mode="corr") #PCA
```

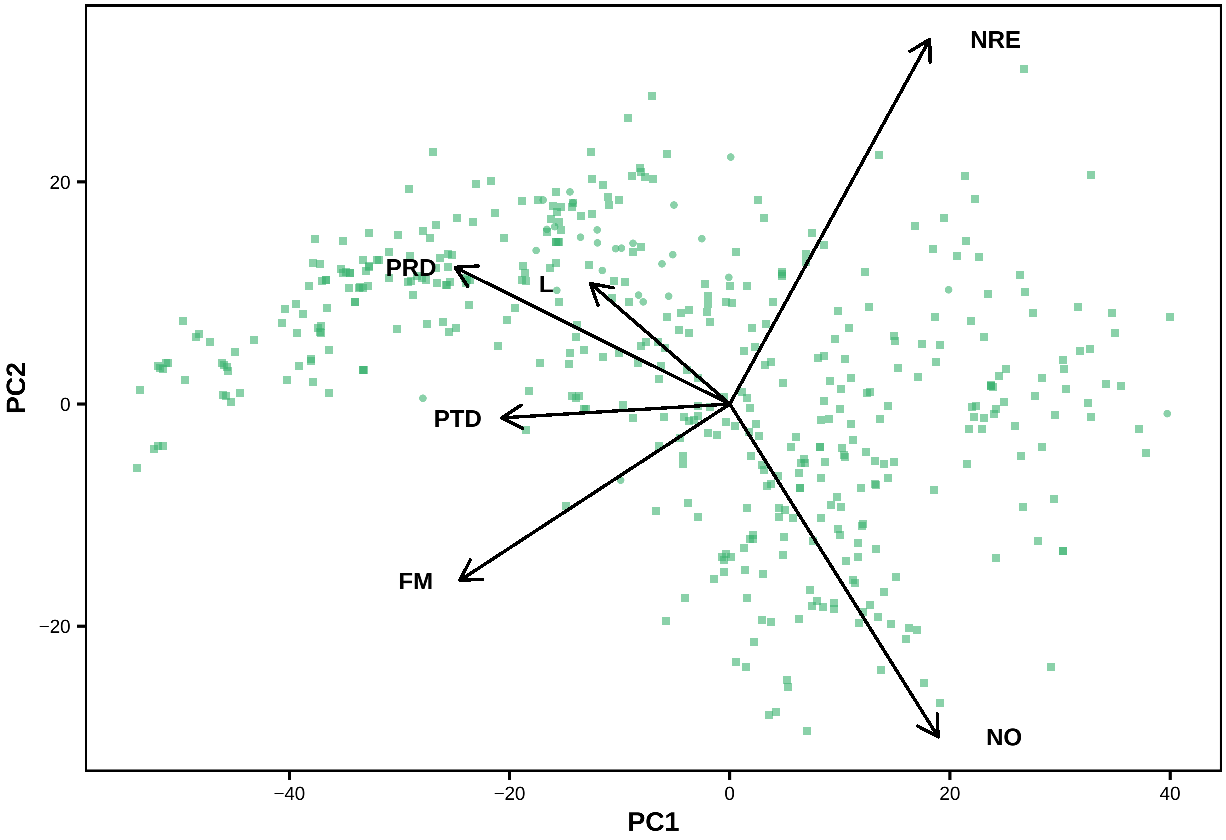

Fig S9: Phylogenetically corrected PCA of life-history traits for walkers. Arrow length indicates the loading of each life-history trait onto PCA axes. Squares represent the position of non-migratory species along the PCA axes and circles of migratory species.

	PC1	PC2	PC3	PC4	PC5	PC6
Standard deviation	1.3617097	1.0645006	0.9694059	0.9117188	0.8091637	0.7660683
Proportion of Variance	0.3090422	0.1888602	0.1566246	0.1385385	0.1091243	0.0978101
Cumulative Proportion	0.3090422	0.4979024	0.6545271	0.7930656	0.9021899	1.0000000

Table S15: Importance of components of the PCA for walkers.

	PC1	PC2	PC3	PC4	PC5	PC6
LongR	-0.2531948	0.2171038	0.9205767	0.1669818	0.0628914	-0.0972552
develR	-0.4140603	-0.0249974	0.0659051	-0.8844878	-0.2031045	-0.0039620
femR	-0.4903060	-0.3177375	-0.1431148	0.3450324	-0.5002134	-0.5185559
embriR	-0.4989682	0.2461733	-0.2816993	0.0394129	0.7293162	-0.2786026
NumclR	0.3631519	0.6567641	-0.0776731	-0.1399897	-0.2250327	-0.6004262
SizeclR	0.3784151	-0.5994527	0.2057317	-0.2227119	0.3493393	-0.5324419

Table S16: Eigenvectors of the PCA for walkers.

S3.5 Birds PCA

```
B_mat <- as.matrix(dfB[,c(17:22)]) # life-history traits selected
rownames(B_mat) <- dfB$animal
PCA_B <- phyl.pca(treeB, B_mat, method="lambda",mode="corr") #PCA
```

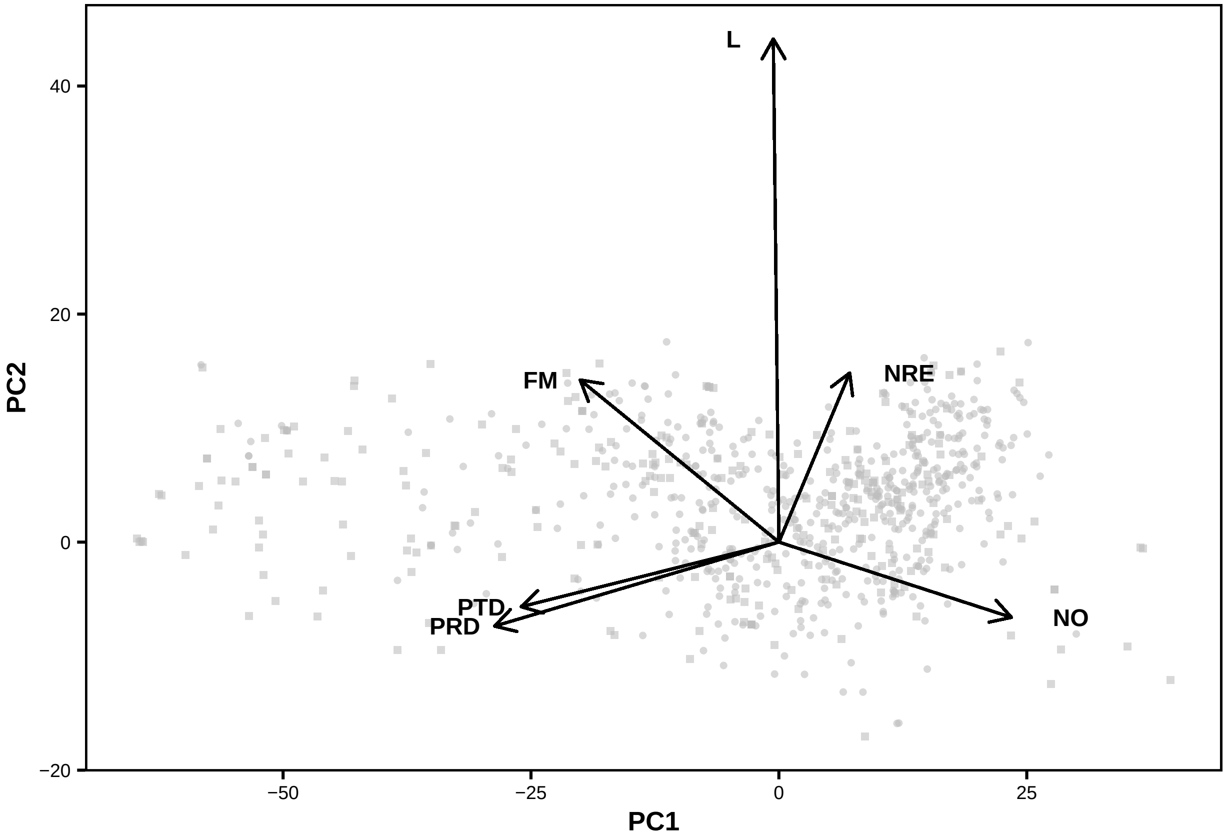

Fig S10: Phylogenetically corrected PCA of life-history traits for birds. Arrow length indicates the loading of each life-history trait onto PCA axes. Squares represent the position of non-migratory species along the PCA axes and circles of migratory species.

	PC1	PC2	PC3	PC4	PC5	PC6
Standard deviation	1.3199407	1.0182641	1.0002527	0.9408696	0.8731106	0.7568563
Proportion of Variance	0.2903739	0.1728103	0.1667509	0.1475393	0.1270537	0.0954719
Cumulative Proportion	0.2903739	0.4631842	0.6299351	0.7774744	0.9045281	1.0000000

Table S17: Importance of components of the PCA for birds.

	PC1	PC2	PC3	PC4	PC5	PC6
LongR	-0.0112342	0.8826280	-0.1792517	0.3959055	-0.1219518	-0.1307558
develR	-0.5198086	-0.1133946	0.1055365	0.5464678	0.1945191	0.6077319
femR	-0.4006813	0.2844426	-0.2885483	-0.5676289	0.5884499	0.0825286
embriR	-0.5732902	-0.1473401	0.2200228	0.1875861	0.0746934	-0.7486331
NumclR	0.1428721	0.2967607	0.8977321	-0.1511317	0.2367877	0.0817836
SizeclR	0.4690984	-0.1320075	-0.1383394	0.4054626	0.7344176	-0.1990321

Table S18: Eigenvectors of the PCA for birds.

S3.5 Mammals PCA

```
M_mat <- as.matrix(dfM[,c(17:22)]) # life-history traits selected
rownames(M_mat) <- dfM$animal
PCA_M <- phyl.pca(treeM, M_mat, method="lambda",mode="corr") #PCA
```

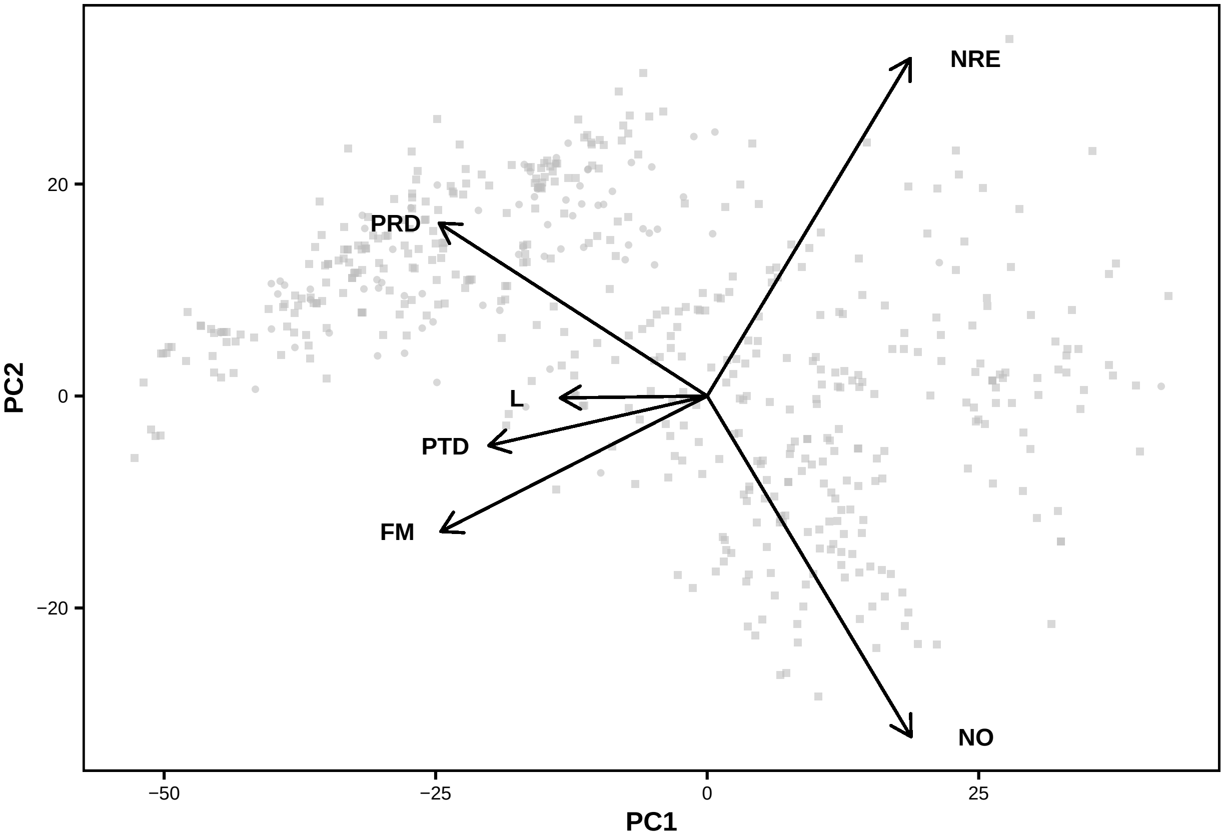

Fig S11: Phylogenetically corrected PCA of life-history traits for mammals. Arrow length indicates the loading of each life-history trait onto PCA axes. Squares represent the position of non-migratory species

along the PCA axes and circles of migratory species.

	PC1	PC2	PC3	PC4	PC5	PC6
Standard deviation	1.3574501	1.0581841	0.9707553	0.9190464	0.8094026	0.7716417
Proportion of Variance	0.3071118	0.1866256	0.1570610	0.1407744	0.1091888	0.0992385
Cumulative Proportion	0.3071118	0.4937374	0.6507984	0.7915727	0.9007615	1.0000000

Table S19: Importance of components of the PCA for mammals.

	PC1	PC2	PC3	PC4	PC5	PC6
LongR	-0.2703663	-0.0035742	0.9421282	-0.1446033	0.0633022	-0.1198602
develR	-0.4021158	-0.0936483	0.0375150	0.8849257	-0.2106244	0.0258719
femR	-0.4903787	-0.2557147	-0.2100365	-0.3700879	-0.5919447	-0.4033111
embriR	-0.4936382	0.3262984	-0.2391318	-0.0136044	0.6462406	-0.4181562
NumclR	0.3743126	0.6370739	0.0897522	0.1661205	-0.3507078	-0.5434882
SizeclR	0.3754266	-0.6430345	0.0404010	0.1767772	0.2461886	-0.5933550

Table S20: Eigenvectors of the PCA for mammals.

Appendix S4: Binomial bayesian comparative analyses

S4.1 All species MCMC

```
library(MCMCglmm)

scores_full <- as.data.frame(PCA_full$S) # extracting scores from the PCA
scores_full$animal <- rownames(scores_full)
rownames(scores_full) <- c()
scores_full <- scores_full[,c(7,1,2)] # subseting species name, PC1 and PC2
full_MCD <- merge(df, scores_full) # creating dataframe for MCMC analysis
full_MCDs <- full_MCD[,c(1,6,14,15,16,23,24,10)]


Full_MCMC <- MCMCglmm(Mig_bi ~ Loc + Lat + PC1 + PC2 + mass + PC1*Loc + PC2*Loc + mass*Loc,
                      data=full_MCDs, family="ordinal",
                      random=~animal, ginverse=list(animal=inverseA(tree)$Ainv),
                      prior=list(R=list(V=1, fix=1),
                                 G=list(G1=list(V=1, nu=1000, alpha.mu=0, alpha.V=1))),
                      pl=TRUE, nitt=5000000,thin=2500,burnin=100000, verbose =TRUE)
```

```
# Phylogenetic variance
mean(Full_MCMC$VCV[, "animal"])
```

```
## [1] 16.82539
```

```
# Heritability
mean(Full_MCMC$VCV[, "animal"]/(Full_MCMC$VCV[, "animal"] + Full_MCMC$VCV[, "units"] + 1))
```

```
## [1] 0.8881193
```

```r
# Gelman and Rubin's convergence diagnostic
gelman.diag(mcmc.list(Full_MCMC$Sol, Full_MCMC1$Sol))$mpsrf
```

```
## [1] 1.008362
```

	post.mean	l.95..CI	u.95..CI	eff.samp	pMCMC
(Intercept)	-0.9066886	-6.1356606	4.3654561	2119.997	0.7142857
LocS	-10.9155011	-24.2916270	3.5709351	1960.000	0.1306122
LocW	-16.3768523	-22.6663112	-10.0876928	1194.710	0.0005102
Lat	0.0869379	0.0700902	0.1036880	1960.000	0.0005102
PC1	0.0434197	0.0118609	0.0753719	1325.134	0.0051020
PC2	0.0133743	-0.0318964	0.0631880	1723.208	0.5897959
mass	-0.3761150	-0.6217926	-0.1090423	1960.000	0.0030612
LocS:PC1	-0.0972614	-0.2106673	0.0162744	1960.000	0.1010204
LocW:PC1	0.0234757	-0.0506695	0.0980251	1471.350	0.5500000
LocS:PC2	-0.0046899	-0.2114059	0.2049778	1960.000	0.9612245
LocW:PC2	-0.0916412	-0.2012453	0.0054624	1809.957	0.0765306
LocS:mass	0.8815961	0.1560648	1.5570207	1960.000	0.0102041
LocW:mass	1.3231353	0.7477108	1.9013871	1283.874	0.0005102

Table S21: Posterior distributions of the parameter estimates of the binomial bayesian comparative analysis that includes all species.

Without non-significant interactions

```r
Full_MCMCr <- MCMCglmm(Mig_bi ~ Loc + Lat + PC1 + PC2 + mass + mass*Loc,
                      data=full_MCDs, family="ordinal",
                      random=~animal, ginverse=list(animal=inverseA(tree)$Ainv),
                      prior=list(R=list(V=1, fix=1),
                                 G=list(G1=list(V=1, nu=1000, alpha.mu=0, alpha.V=1))),
                      pl=TRUE, nitt=5000000,thin=2500,burnin=100000, verbose =TRUE)
```

```r
# Phylogenetic variance
mean(Full_MCMCr$VCV[, "animal"])
```

```
## [1] 15.81595
```

```r
# Heritability
mean(Full_MCMCr$VCV[, "animal"]/(Full_MCMCr$VCV[, "animal"] + Full_MCMCr$VCV[, "units"] + 1))
```

```
## [1] 0.8822247
```

```r
# Gelman and Rubin's convergence diagnostic
gelman.diag(mcmc.list(Full_MCMCr$Sol, Full_MCMCr1$Sol))$mpsrf
```

```
## [1] 1.001622
```

	post.mean	l.95..CI	u.95..CI	eff.samp	pMCMC
(Intercept)	-0.8983773	-5.9114914	4.0854656	2116.111	0.7112245
LocS	-3.1835895	-10.4641477	4.5683556	1960.000	0.3704082
LocW	-13.8417211	-19.0894507	-8.4174975	1783.503	0.0005102
Lat	0.0838216	0.0687948	0.0992757	1960.000	0.0005102
PC1	0.0385089	0.0088884	0.0640985	1960.000	0.0030612
PC2	0.0052328	-0.0306168	0.0484669	1960.000	0.7867347
mass	-0.3783089	-0.6204005	-0.1338038	1960.000	0.0040816
LocS:mass	0.5667761	-0.0289563	1.0667423	1960.000	0.0397959
LocW:mass	1.1899852	0.7526819	1.6905465	1960.000	0.0005102

Table S22: Posterior distributions of the parameter estimates of the binomial bayesian comparative analysis without non-significant interactions that includes all species.

S4.2 Flyers MCMC

```
library(MCMCglmm)

scores_F <- as.data.frame(PCA_F$S) # extracting scores from the PCA
scores_F$animal <- rownames(scores_F)
rownames(scores_F) <- c()
scores_F <- scores_F[,c(7,1,2)] # subseting species name, PC1 and PC2
F_MCD <- merge(dfF, scores_F) # creating dataframe for MCMC analysis
F_MCDs <- F_MCD[,c(1,6,14,15,23,24,10)]


F_MCMC <- MCMCglmm(Mig_bi ~ Lat + PC1 + PC2 + mass,
                    data=F_MCDs, family="ordinal",
                    random=~animal, ginverse=list(animal=inverseA(treeF)$Ainv),
                    prior=list(R=list(V=1, fix=1),
                               G=list(G1=list(V=1, nu=1000, alpha.mu=0, alpha.V=1))),
                    pl=TRUE, nitt=5000000,thin=2500,burnin=100000, verbose =TRUE)
```

```
# Phylogenetic variance
mean(F_MCMC$VCV[, "animal"])
```

```
## [1] 16.40681
```

```
# Heritability
mean(F_MCMC$VCV[, "animal"]/(F_MCMC$VCV[, "animal"] + F_MCMC$VCV[, "units"] + 1))
```

```
## [1] 0.8852792
```

```
# Gelman and Rubin's convergence diagnostic
gelman.diag(mcmc.list(F_MCMC$Sol, F_MCMC1$Sol))$mpsrf
```

```
## [1] 0.9998411
```

	post.mean	l.95..CI	u.95..CI	eff.samp	pMCMC
(Intercept)	-1.4383969	-6.7529762	3.7199640	1960.000	0.5806122
Lat	0.0978353	0.0787341	0.1178589	1828.443	0.0005102
PC1	0.0324594	0.0062335	0.0563482	2093.637	0.0081633
PC2	-0.0119609	-0.0576318	0.0319676	2176.990	0.5867347
mass	-0.4197620	-0.6694027	-0.1651425	1960.000	0.0005102

Table S23: Posterior distributions of the parameter estimates of the binomial bayesian comparative analysis that includes flyers.

S4.3 Swimmers MCMC

```
library(MCMCglmm)

scores_S <- as.data.frame(PCA_S$S) # extracting scores from the PCA
scores_S$animal <- rownames(scores_S)
rownames(scores_S) <- c()
scores_S <- scores_S[,c(7,1,2)] # subseting species name, PC1 and PC2
S_MCD <- merge(dfS, scores_S) # creating dataframe for MCMC analysis
S_MCDs <- S_MCD[,c(1,6,14,15,23,24,10)]


S_MCMC <- MCMCglmm(Mig_bi ~ Lat + PC1 + PC2 + mass,
                      data=S_MCDs, family="ordinal",
                      random=~animal, ginverse=list(animal=inverseA(treeS)$Ainv),
                      prior=list(R=list(V=1, fix=1),
                                 G=list(G1=list(V=1, nu=1000, alpha.mu=0, alpha.V=1))),
                      pl=TRUE, nitt=5000000,thin=2500,burnin=100000, verbose =TRUE)
```

```
# Phylogenetic variance
mean(S_MCMC$VCV[, "animal"])
```

```
## [1] 1.854563
```

```
# Heritability
mean(S_MCMC$VCV[, "animal"]/(S_MCMC$VCV[, "animal"] + S_MCMC$VCV[, "units"] + 1))
```

```
## [1] 0.3862757
```

```
# Gelman and Rubin's convergence diagnostic
gelman.diag(mcmc.list(S_MCMC$Sol, S_MCMC1$Sol))$mpsrf
```

```
## [1] 0.9999223
```

	post.mean	l.95..CI	u.95..CI	eff.samp	pMCMC
(Intercept)	-4.9228922	-10.6215769	0.9049466	1960.000	0.0877551
Lat	0.0203076	-0.0107220	0.0535083	1774.998	0.1959184
PC1	-0.0245271	-0.0774013	0.0365261	1960.000	0.3897959
PC2	-0.0051501	-0.1118711	0.0981837	1960.000	0.9224490
mass	0.3651118	-0.0526942	0.7810295	1960.000	0.0806122

Table S24: Posterior distributions of the parameter estimates of the binomial bayesian comparative analysis that includes swimmers.

S4.4 Walkers MCMC

```
library(MCMCglmm)

scores_W <- as.data.frame(PCA_W$S) # extracting scores from the PCA
scores_W$animal <- rownames(scores_W)
rownames(scores_W) <- c()
scores_W <- scores_W[,c(7,1,2)] # subseting species name, PC1 and PC2
W_MCD <- merge(dfW, scores_W) # creating dataframe for MCMC analysis
W_MCDs <- W_MCD[,c(1,6,14,15,23,24,10)]


W_MCMC <- MCMCglmm(Mig_bi ~ Lat + PC1 + PC2 + mass,
                      data=W_MCDs, family="ordinal",
                      random=~animal, ginverse=list(animal=inverseA(treeW)$Ainv),
                      prior=list(R=list(V=1, fix=1),
                                 G=list(G1=list(V=1, nu=1000, alpha.mu=0, alpha.V=1))),
                      pl=TRUE, nitt=5000000,thin=2500,burnin=100000, verbose =TRUE)
```

```
# Phylogenetic variance
mean(W_MCMC$VCV[, "animal"])
```

```
## [1] 2.383055
```

```
# Heritability
mean(W_MCMC$VCV[, "animal"]/(W_MCMC$VCV[, "animal"] + W_MCMC$VCV[, "units"] + 1))
```

```
## [1] 0.427257
```

```
# Gelman and Rubin's convergence diagnostic
gelman.diag(mcmc.list(W_MCMC$Sol, W_MCMC1$Sol))$mpsrf
```

```
## [1] 1.002358
```

	post.mean	l.95..CI	u.95..CI	eff.samp	pMCMC
(Intercept)	-13.0438186	-18.6751276	-8.6444957	1182.409	0.0005102
Lat	0.0722010	0.0411716	0.1040680	2637.580	0.0005102
PC1	0.0580132	-0.0038683	0.1205813	1235.395	0.0377551
PC2	0.0771234	0.0010504	0.1640567	1960.000	0.0622449
mass	0.7415425	0.3620523	1.1484300	1262.066	0.0005102

Table S25: Posterior distributions of the parameter estimates of the binomial bayesian comparative analysis that includes walkers.

S4.5 Birds MCMC

```
library(MCMCglmm)

scores_B <- as.data.frame(PCA_B$S) # extracting scores from the PCA
scores_B$animal <- rownames(scores_B)
rownames(scores_B) <- c()
scores_B <- scores_B[,c(7,1,2)] # subseting species name, PC1 and PC2
B_MCD <- merge(dfB, scores_B) # creating dataframe for MCMC analysis
B_MCDs <- B_MCD[,c(1,6,14,15,23,24,10)]

B_MCMC <- MCMCglmm(Mig_bi ~ Lat + PC1 + PC2 + mass,
                   data=B_MCDs, family="ordinal",
                   random=~animal, ginverse=list(animal=inverseA(treeB)$Ainv),
                   prior=list(R=list(V=1, fix=1),
                              G=list(G1=list(V=1, nu=1000, alpha.mu=0, alpha.V=1))),
                   pl=TRUE, nitt=5000000,thin=2500,burnin=100000, verbose =TRUE)
```

```
# Phylogenetic variance
mean(B_MCMC$VCV[, "animal"])
```

```
## [1] 8.535861
```

```
# Heritability
mean(B_MCMC$VCV[, "animal"]/(B_MCMC$VCV[, "animal"] + B_MCMC$VCV[, "units"] + 1))
```

```
## [1] 0.7987181
```

```
# Gelman and Rubin's convergence diagnostic
gelman.diag(mcmc.list(B_MCMC$Sol, B_MCMC1$Sol))$mpsrf
```

```
## [1] 1.000255
```

	post.mean	l.95..CI	u.95..CI	eff.samp	pMCMC
(Intercept)	-0.8656578	-3.4828330	1.9301962	2272.530	0.5387755
Lat	0.1119453	0.0873137	0.1375525	1895.398	0.0005102
PC1	0.0391955	0.0093132	0.0703241	1960.000	0.0071429
PC2	-0.0174089	-0.0719417	0.0352231	1960.000	0.5316327
mass	-0.5403514	-0.8630210	-0.2400613	1960.000	0.0005102

Table S26: Posterior distributions of the parameter estimates of the binomial bayesian comparative analysis that includes birds.

S4.6 Mammals MCMC

```
library(MCMCglmm)

scores_M <- as.data.frame(PCA_M$S) # extracting scores from the PCA
scores_M$animal <- rownames(scores_M)
rownames(scores_M) <- c()
scores_M <- scores_M[,c(7,1,2)] # subseting species name, PC1 and PC2
M_MCD <- merge(dfM, scores_M) # creating dataframe for MCMC analysis
M_MCDs <- M_MCD[,c(1,6,14,15,23,24,10)]

M_MCMC_loc <- MCMCglmm(Mig_bi ~ Loc + Lat + PC1 + PC2 + mass + mass*Loc,
                    data=M_MCDs, family="ordinal",
                    random=~animal, ginverse=list(animal=inverseA(treeM)$Ainv),
                    prior=list(R=list(V=1, fix=1),
                               G=list(G1=list(V=1, nu=1000, alpha.mu=0, alpha.V=1))),
                    pl=TRUE, nitt=5000000,thin=2500,burnin=100000, verbose =TRUE)
```

```
# Phylogenetic variance
mean(M_MCMC_loc$VCV[, "animal"])
```

```
## [1] 6.08782
```

```
# Heritability
mean(M_MCMC_loc$VCV[, "animal"]/(M_MCMC_loc$VCV[, "animal"] + M_MCMC_loc$VCV[, "units"] + 1))
```

```
## [1] 0.7159922
```

```
# Gelman and Rubin's convergence diagnostic
gelman.diag(mcmc.list(M_MCMC_loc$Sol, M_MCMC_loc1$Sol))$mpsrf
```

```
## [1] 1.002374
```

	post.mean	l.95..CI	u.95..CI	eff.samp	pMCMC
(Intercept)	-12.1389718	-17.3229304	-6.6065230	1315.973	0.0005102
LocF	8.4927535	0.5644184	15.7053693	1442.490	0.0132653
LocS	8.4402309	0.2024850	17.6587335	1530.659	0.0408163
Lat	0.0442575	0.0201459	0.0670070	1960.000	0.0005102
PC1	0.0410885	-0.0090754	0.0916703	1827.656	0.0857143
PC2	-0.0141328	-0.0774347	0.0509527	2296.343	0.6642857
mass	0.6960369	0.2774595	1.0676081	1469.071	0.0005102
LocF:mass	0.2972653	-1.3625300	2.0927675	1960.000	0.7673469
LocS:mass	-0.4603450	-1.1095055	0.1682564	1697.006	0.1551020

Table S27: Posterior distributions of the parameter estimates of the binomial bayesian comparative analysis that includes mammals.

S4.7 Flyers & walkers from temperate areas

```
library(MCMCglmm)

Temp_MCDs <- subset(full_MCDs, full_MCDs$Loc != "S" &
                    full_MCDs$Lat > 23.5 & full_MCDs$Lat < 66.5)

Temp_MCMC_WF <- MCMCglmm(Mig_bi ~ Loc + Lat + PC1 + PC2 + mass + mass*Loc,
                         data=Temp_MCDs, family="ordinal",
                         random=~animal, ginverse=list(animal=inverseA(tree)$Ainv),
                         prior=list(R=list(V=1, fix=1),
                                    G=list(G1=list(V=1, nu=1000, alpha.mu=0, alpha.V=1))),
                         pl=TRUE, nitt=5000000,thin=2500,burnin=100000, verbose =TRUE)
```

```
# Phylogenetic variance
mean(Temp_MCMC_WF$VCV[, "animal"])
```

```
## [1] 17.9316
```

```
# Heritability
mean(Temp_MCMC_WF$VCV[, "animal"]/(Temp_MCMC_WF$VCV[, "animal"] + Temp_MCMC_WF$VCV[, "units"] + 1))
```

```
## [1] 0.8940578
```

```
# Gelman and Rubin's convergence diagnostic
gelman.diag(mcmc.list(Temp_MCMC_WF$Sol, Temp_MCMC_WF1$Sol))$mpsrf
```

```
## [1] 1.00062
```

	post.mean	l.95..CI	u.95..CI	eff.samp	pMCMC
(Intercept)	-2.4714956	-7.7431043	3.0556653	1960.000	0.3775510
LocW	-16.5423839	-23.1957818	-9.9167165	1830.895	0.0005102
Lat	0.1243673	0.0946854	0.1548563	1960.000	0.0005102
PC1	0.0617999	0.0241096	0.0985271	1766.439	0.0005102
PC2	-0.0290863	-0.0804022	0.0222790	2263.825	0.2469388
mass	-0.3927929	-0.6750323	-0.1019728	1960.000	0.0051020
LocW:mass	1.4181524	0.8208067	2.0811183	1960.000	0.0005102

Table S28: Posterior distributions of the parameter estimates of the binomial bayesian comparative analysis that includes flyers and walkers from temperate areas.

S4.8 Flyers & walkers from the Northern Hemisphere

```
library(MCMCglmm)

PosLat_MCDs <- subset(full_MCDs, full_MCDs$Loc != "S" &
                      full_MCDs$Lat > 0)

PosLat_MCMC_WF <- MCMCglmm(Mig_bi ~ Loc + Lat + PC1 + PC2 + mass + mass*Loc,
                           data=PosLat_MCDs, family="ordinal",
```

```
                        random=~animal, ginverse=list(animal=inverseA(tree)$Ainv),
                        prior=list(R=list(V=1, fix=1),
                                   G=list(G1=list(V=1, nu=1000, alpha.mu=0, alpha.V=1))),
                        pl=TRUE, nitt=5000000,thin=2500,burnin=100000, verbose =TRUE)
```

```
# Phylogenetic variance
mean(PosLat_MCMC_noS$VCV[, "animal"])
```

```
## [1] 14.55107
```

```
# Heritability
mean(PosLat_MCMC_noS$VCV[, "animal"]/(PosLat_MCMC_noS$VCV[, "animal"] + PosLat_MCMC_noS$VCV[, "units"] +
```

```
## [1] 0.8719824
```

```
# Gelman and Rubin's convergence diagnostic
gelman.diag(mcmc.list(PosLat_MCMC_noS$Sol, PosLat_MCMC_noS1$Sol))$mpsrf
```

```
## [1] 1.002786
```

	post.mean	l.95..CI	u.95..CI	eff.samp	pMCMC
(Intercept)	-1.2990845	-6.3390886	3.3286959	1960	0.6102041
LocW	-13.3449396	-19.4817571	-8.2543925	1960	0.0005102
Lat	0.1007527	0.0807638	0.1219774	1960	0.0005102
PC1	0.0341588	0.0006919	0.0642410	1960	0.0295918
PC2	-0.0314731	-0.0812320	0.0176621	1960	0.1897959
mass	-0.4105561	-0.6724913	-0.1447858	1960	0.0010204
LocW:mass	1.2802032	0.8147695	1.8030108	1960	0.0005102

Table S29: Posterior distributions of the parameter estimates of the binomial bayesian comparative analysis that includes flyers and walkers from the Northern Hemisphere.

S4.9 Passeriformes

```
library(MCMCglmm)

full_MCD_pas <- subset(full_MCD, full_MCD$order == "Passeriformes")
full_MCDs_pas <- full_MCD_pas[,c(1,6,14:18,10)]

pas_MCMC <- MCMCglmm(Mig_bi ~ Lat + PC1 + PC2 + mass,
                        data=full_MCDs_pas, family="ordinal",
                        random=~animal, ginverse=list(animal=inverseA(tree)$Ainv),
                        prior=list(R=list(V=1, fix=1),
                                   G=list(G1=list(V=1, nu=1000, alpha.mu=0, alpha.V=1))),
                        pl=TRUE, nitt=5000000,thin=2500,burnin=100000, verbose =TRUE)
```

```
# Phylogenetic variance
mean(pas_MCMC$VCV[, "animal"])
```

```
## [1] 14.23208
```

```
# Heritability
mean(pas_MCMC$VCV[, "animal"]/(pas_MCMC$VCV[, "animal"] + pas_MCMC$VCV[, "units"] + 1))
```

```
## [1] 0.8672155
```

```r
# Gelman and Rubin's convergence diagnostic
gelman.diag(mcmc.list(pas_MCMC$Sol, pas_MCMC1$Sol))$mpsrf
```

```
## [1] 1.003555
```

	post.mean	l.95..CI	u.95..CI	eff.samp	pMCMC
(Intercept)	-4.1371672	-11.8287189	3.2037745	1960	0.2500000
Lat	0.1155141	0.0757775	0.1532923	1960	0.0005102
PC1	0.1722532	0.0423682	0.2943941	1960	0.0112245
PC2	0.0268760	-0.0544138	0.1111358	1960	0.5183673
mass	-0.6027636	-1.2280192	-0.1019401	1960	0.0295918

Table S30: Posterior distributions of the parameter estimates of the binomial bayesian comparative analysis that includes Passeriformes.

S4.10 Non-Passeriform flyers

```r
library(MCMCglmm)

full_MCD_Npas <- subset(full_MCD, full_MCD$order != "Passeriformes" & full_MCD$Loc=="F")
full_MCDs_Npas <- full_MCD_Npas[,c(1,6,14:18,10)]

Npas_F_MCMC <- MCMCglmm(Mig_bi ~ Lat + PC1 + PC2 + mass,
                        data=full_MCDs_Npas, family="ordinal",
                        random=~animal, ginverse=list(animal=inverseA(tree)$Ainv),
                        prior=list(R=list(V=1, fix=1),
                                   G=list(G1=list(V=1, nu=1000, alpha.mu=0, alpha.V=1))),
                        pl=TRUE, nitt=5000000,thin=2500,burnin=100000, verbose =TRUE)
```

```r
# Phylogenetic variance
mean(Npas_F_MCMC$VCV[, "animal"])
```

```
## [1] 10.02423
```

```r
# Heritability
mean(Npas_F_MCMC$VCV[, "animal"]/(Npas_F_MCMC$VCV[, "animal"] + Npas_F_MCMC$VCV[, "units"] + 1))
```

```
## [1] 0.821176
```

```r
# Gelman and Rubin's convergence diagnostic
gelman.diag(mcmc.list(Npas_F_MCMC$Sol, Npas_F_MCMC1$Sol))$mpsrf
```

```
## [1] 1.004066
```

	post.mean	l.95..CI	u.95..CI	eff.samp	pMCMC
(Intercept)	-0.6648207	-4.4730954	3.4619060	2237.529	0.7387755
Lat	0.0828392	0.0636996	0.1020813	1960.000	0.0005102
PC1	0.0319848	0.0010264	0.0620996	1960.000	0.0357143
PC2	0.0016883	-0.0571291	0.0574501	1960.000	0.9377551
mass	-0.3601233	-0.6143451	-0.1151954	2297.599	0.0030612

Table S31: Posterior distributions of the parameter estimates of the binomial bayesian comparative analysis that includes non-Passeriform flyers.

S4.11 Non-Passeriform birds

```
library(MCMCglmm)

full_MCD_NpasB <- subset(full_MCD, full_MCD$order != "Passeriformes" & full_MCD$Class=="Bird")
full_MCDs_NpasB <- full_MCD_NpasB[,c(1,6,14:18,10)]

Npas_B_MCMC <- MCMCglmm(Mig_bi ~ Lat + PC1 + PC2 + mass,
                        data=full_MCDs_NpasB, family="ordinal",
                        random=~animal, ginverse=list(animal=inverseA(tree)$Ainv),
                        prior=list(R=list(V=1, fix=1),
                                   G=list(G1=list(V=1, nu=1000, alpha.mu=0, alpha.V=1))),
                        pl=TRUE, nitt=5000000,thin=2500,burnin=100000, verbose =TRUE)
```

```
# Phylogenetic variance
mean(Npas_B_MCMC$VCV[, "animal"])
```

```
## [1] 9.947972
```

```
# Heritability
mean(Npas_B_MCMC$VCV[, "animal"]/(Npas_B_MCMC$VCV[, "animal"] + Npas_B_MCMC$VCV[, "units"] + 1))
```

```
## [1] 0.8196994
```

```
# Gelman and Rubin's convergence diagnostic
gelman.diag(mcmc.list(Npas_B_MCMC$Sol, Npas_B_MCMC1$Sol))$mpsrf
```

```
## [1] 1.004252
```

	post.mean	l.95..CI	u.95..CI	eff.samp	pMCMC
(Intercept)	-0.7716995	-6.4516073	4.6777288	1960.000	0.7642857
Lat	0.0841462	0.0648727	0.1021162	1960.000	0.0005102
PC1	0.0323259	0.0027502	0.0682500	1960.000	0.0438776
PC2	0.0023163	-0.0558031	0.0591758	2143.462	0.9612245
mass	-0.4089490	-0.6884351	-0.1691725	1960.000	0.0020408

Table S32: Posterior distributions of the parameter estimates of the binomial bayesian comparative analysis that includes non-Passeriform birds.

S4.12 Artiodactyla

```
library(MCMCglmm)

full_MCD_art <- subset(full_MCD, full_MCD$order == "Artiodactyla")
full_MCDs_art <- full_MCD_art[,c(1,6,14:18,10)]

art_MCMC <- MCMCglmm(Mig_bi ~ Lat + PC1 + PC2 + mass,
                     data=full_MCDs_art, family="ordinal",
                     random=~animal, ginverse=list(animal=inverseA(tree)$Ainv),
                     prior=list(R=list(V=1, fix=1),
                                G=list(G1=list(V=1, nu=1000, alpha.mu=0, alpha.V=1))),
                     pl=TRUE, nitt=5000000,thin=2500,burnin=100000, verbose =TRUE)
```

```
# Phylogenetic variance
mean(art_MCMC$VCV[, "animal"])
```

```
## [1] 1.870495
```

```
# Heritability
mean(art_MCMC$VCV[, "animal"]/(art_MCMC$VCV[, "animal"] + art_MCMC$VCV[, "units"] + 1))
```

```
## [1] 0.3408118
```

```
# Gelman and Rubin's convergence diagnostic
gelman.diag(mcmc.list(art_MCMC$Sol, art_MCMC1$Sol))$mpsrf
```

```
## [1] 1.003079
```

	post.mean	l.95..CI	u.95..CI	eff.samp	pMCMC
(Intercept)	-8.8688843	-16.7226496	-0.8095447	1798.535	0.0183673
Lat	0.0993128	0.0583731	0.1453260	1960.000	0.0005102
PC1	0.0659793	-0.0296338	0.1572767	1960.000	0.1571429
PC2	-0.0424655	-0.1904360	0.1162727	1960.000	0.6091837
mass	0.4688912	-0.1531634	1.0515301	1960.000	0.1316327

Table S33: Posterior distributions of the parameter estimates of the binomial bayesian comparative analysis that includes Artiodactyla.

S4.13 Accipitriformes

```
library(MCMCglmm)

full_MCD_acc <- subset(full_MCD, full_MCD$order == "Accipitriformes")
full_MCDs_acc <- full_MCD_acc[,c(1,6,14:18,10)]

acc_MCMC <- MCMCglmm(Mig_bi ~ Lat + PC1 + PC2 + mass,
                     data=full_MCDs_acc, family="ordinal",
                     random=~animal, ginverse=list(animal=inverseA(tree)$Ainv),
                     prior=list(R=list(V=1, fix=1),
```

```
                              G=list(G1=list(V=1, nu=1000, alpha.mu=0, alpha.V=1))),
                   pl=TRUE, nitt=5000000,thin=2500,burnin=100000, verbose =TRUE)
```

```
# Phylogenetic variance
mean(acc_MCMC$VCV[, "animal"])
```

```
## [1] 1.200975
```

```
# Heritability
mean(acc_MCMC$VCV[, "animal"]/(acc_MCMC$VCV[, "animal"] + acc_MCMC$VCV[, "units"] + 1))
```

```
## [1] 0.2675976
```

```
# Gelman and Rubin's convergence diagnostic
gelman.diag(mcmc.list(acc_MCMC$Sol, acc_MCMC1$Sol))$mpsrf
```

```
## [1] 1.000528
```

	post.mean	l.95..CI	u.95..CI	eff.samp	pMCMC
(Intercept)	19.3634526	4.4946699	34.9735555	1818.699	0.0030612
Lat	0.3168526	0.1651269	0.5099394	1819.382	0.0005102
PC1	-0.0083826	-0.1248325	0.1031239	1853.177	0.8734694
PC2	-0.5093046	-0.9280858	-0.1451621	1821.581	0.0020408
mass	-3.9007748	-6.8630535	-1.5242069	1960.000	0.0005102

Table S34: Posterior distributions of the parameter estimates of the binomial bayesian comparative analysis that includes Accipitriformes.

S4.14 Pelecaniformes

```
library(MCMCglmm)

full_MCD_pel <- subset(full_MCD, full_MCD$order == "Pelecaniformes")
full_MCDs_pel <- full_MCD_pel[,c(1,6,14:18,10)]

pel_MCMC <- MCMCglmm(Mig_bi ~ Lat + PC1 + PC2 + mass,
                     data=full_MCDs_pel, family="ordinal",
                     random=~animal, ginverse=list(animal=inverseA(tree)$Ainv),
                     prior=list(R=list(V=1, fix=1),
                                G=list(G1=list(V=1, nu=1000, alpha.mu=0, alpha.V=1))),
                     pl=TRUE, nitt=5000000,thin=2500,burnin=100000, verbose =TRUE)
```

```
# Phylogenetic variance
mean(pel_MCMC$VCV[, "animal"])
```

```
## [1] 0.9291986
```

```
# Heritability
mean(pel_MCMC$VCV[, "animal"]/(pel_MCMC$VCV[, "animal"] + pel_MCMC$VCV[, "units"] + 1))
```

```
## [1] 0.2306856
```

```r
# Gelman and Rubin's convergence diagnostic
gelman.diag(mcmc.list(pel_MCMC$Sol, pel_MCMC1$Sol))$mpsrf
```

```
## [1] 1.002576
```

	post.mean	l.95..CI	u.95..CI	eff.samp	pMCMC
(Intercept)	4.6074996	-10.5427925	22.0155897	1960	0.5755102
Lat	0.1296327	0.0503814	0.2156776	1960	0.0010204
PC1	-0.0918302	-0.3754745	0.2259086	1960	0.5551020
PC2	-0.0799152	-0.5582730	0.3665015	1960	0.7693878
mass	-0.9325980	-2.8862531	1.1054377	1960	0.3520408

Table S35: Posterior distributions of the parameter estimates of the binomial bayesian comparative analysis that includes Pelecaniformes.

S4.15 Chiroptera

```r
library(MCMCglmm)

full_MCD_chi <- subset(full_MCD, full_MCD$order == "Chiroptera")
full_MCDs_chi <- full_MCD_chi[,c(1,6,14:18,10)]

chi_MCMC <- MCMCglmm(Mig_bi ~ Lat + PC1 + PC2 + mass,
                      data=full_MCDs_chi, family="ordinal",
                      random=~animal, ginverse=list(animal=inverseA(tree)$Ainv),
                      prior=list(R=list(V=1, fix=1),
                                 G=list(G1=list(V=1, nu=1000, alpha.mu=0, alpha.V=1))),
                      pl=TRUE, nitt=5000000,thin=2500,burnin=100000, verbose =TRUE)
```

```r
# Phylogenetic variance
mean(chi_MCMC$VCV[, "animal"])
```

```
## [1] 1.618123
```

```r
# Heritability
mean(chi_MCMC$VCV[, "animal"]/(chi_MCMC$VCV[, "animal"] + chi_MCMC$VCV[, "units"] + 1))
```

```
## [1] 0.3196973
```

```r
# Gelman and Rubin's convergence diagnostic
gelman.diag(mcmc.list(chi_MCMC$Sol, chi_MCMC1$Sol))$mpsrf
```

```
## [1] 1.001997
```

	post.mean	l.95..CI	u.95..CI	eff.samp	pMCMC
(Intercept)	-3.0285011	-9.4292653	3.6022914	1960.000	0.3602041
Lat	0.0326973	-0.0472043	0.1117454	1721.301	0.4010204
PC1	0.0031944	-0.1367752	0.1402428	1960.000	0.9836735
PC2	0.0836916	-0.1770829	0.3200952	1960.000	0.5285714
mass	1.1061591	-0.3495459	2.6073050	1960.000	0.1000000

Table S36: Posterior distributions of the parameter estimates of the binomial bayesian comparative analysis that includes Chiroptera.

Appendix S5: Gaussian bayesian comparative analyses

S5.1 All species MCMC

```
library(MCMCglmm)

Full_MCMC_inv <- MCMCglmm(PC1 ~ Loc + Lat +  Mig_bi + mass + Mig_bi*Loc + mass*Loc,
                      data=full_MCDs, family="gaussian",
                      random=~animal, ginverse=list(animal=inverseA(tree)$Ainv),
                      prior=list(R=list(V=diag(1),nu=0.002),
                                 G=list(G1=list(V=diag(1), nu=0.002))),
                      pl=TRUE, nitt=5000000,thin=2500,burnin=100000, verbose =TRUE)
```

```
# Phylogenetic variance
mean(Full_MCMC_inv$VCV[, "animal"])
```

```
## [1] 631.6292
```

```
# Heritability
mean(Full_MCMC_inv$VCV[, "animal"]/(Full_MCMC_inv$VCV[, "animal"] + Full_MCMC_inv$VCV[, "units"]))
```

```
## [1] 0.9979771
```

```
# Gelman and Rubin's convergence diagnostic
gelman.diag(mcmc.list(Full_MCMC_inv$Sol, Full_MCMC_inv1$Sol))$mpsrf
```

```
## [1] 1.006618
```

	post.mean	l.95..CI	u.95..CI	eff.samp	pMCMC
(Intercept)	-15.0450968	-42.6965577	16.5000449	1960.000	0.3051020
LocS	-24.7206479	-52.4434022	2.9461136	2093.602	0.0867347
LocW	26.6585527	8.4648978	42.8180617	1960.000	0.0051020
Lat	0.0465054	0.0259121	0.0673237	1960.000	0.0005102
Mig_bi	0.8976410	0.1135056	1.7640153	1655.630	0.0336735
mass	0.0085889	-0.6237562	0.6978777	1960.000	0.9755102
LocS:Mig_bi	-0.9811981	-3.6430669	1.9385381	1960.000	0.5081633
LocW:Mig_bi	2.5660225	0.1094179	5.1476415	1960.000	0.0530612
LocS:mass	2.2512639	0.4649686	3.8995095	2237.421	0.0122449
LocW:mass	-0.0884592	-0.9246754	0.7398126	1960.000	0.8387755

Table S37: Posterior distributions of the parameter estimates of the gaussian bayesian comparative analysis that includes all species.

S5.2 Flyers MCMC

```
library(MCMCglmm)

F_MCMC_inv <- MCMCglmm(PC1 ~ Lat + Mig_bi + mass,
                       data=F_MCDs, family="gaussian",
                       random=~animal, ginverse=list(animal=inverseA(treeF)$Ainv),
                       prior=list(R=list(V=diag(1),nu=0.002),
                                  G=list(G1=list(V=diag(1), nu=0.002))),
                       pl=TRUE, nitt=5000000,thin=2500,burnin=100000, verbose =TRUE)
```

```
# Phylogenetic variance
mean(F_MCMC_inv$VCV[, "animal"])
```

```
## [1] 594.104
```

```
# Heritability
mean(F_MCMC_inv$VCV[, "animal"]/(F_MCMC_inv$VCV[, "animal"] + F_MCMC_inv$VCV[, "units"]))
```

```
## [1] 0.9950327
```

```
# Gelman and Rubin's convergence diagnostic
gelman.diag(mcmc.list(F_MCMC_inv$Sol, F_MCMC_inv1$Sol))$mpsrf
```

```
## [1] 1.001766
```

	post.mean	l.95..CI	u.95..CI	eff.samp	pMCMC
(Intercept)	-3.9659070	-34.3296420	25.0649072	1832.217	0.8091837
Lat	0.0813722	0.0526932	0.1120914	1960.000	0.0005102
Mig_bi	1.0418608	0.0265664	1.9964786	1960.000	0.0367347
mass	0.0158368	-0.6726515	0.7465839	1960.000	0.9948980

Table S38: Posterior distributions of the parameter estimates of the gaussian bayesian comparative analysis that includes flyers.

S5.3 Swimmers MCMC

```
library(MCMCglmm)

S_MCMC_inv <- MCMCglmm(PC1 ~ Lat + Mig_bi + mass,
                       data=S_MCDs, family="gaussian",
                       random=~animal, ginverse=list(animal=inverseA(treeS)$Ainv),
                       prior=list(R=list(V=diag(1),nu=0.002),
                                  G=list(G1=list(V=diag(1), nu=0.002))),
                       pl=TRUE, nitt=5000000,thin=2500,burnin=100000, verbose =TRUE)
```

```
# Phylogenetic variance
mean(S_MCMC_inv$VCV[, "animal"])
```

```
## [1] 364.6114
```

```r
# Heritability
mean(S_MCMC_inv$VCV[, "animal"]/(S_MCMC_inv$VCV[, "animal"] + S_MCMC_inv$VCV[, "units"]))
```

```
## [1] 0.9886465
```

```r
# Gelman and Rubin's convergence diagnostic
gelman.diag(mcmc.list(S_MCMC_inv$Sol, S_MCMC_inv1$Sol))$mpsrf
```

```
## [1] 1.002213
```

	post.mean	l.95..CI	u.95..CI	eff.samp	pMCMC
(Intercept)	4.4884476	-32.0883911	39.5531295	1820.276	0.7938776
Lat	0.0826658	-0.0141449	0.1796968	1960.000	0.1000000
Mig_bi	-1.4973533	-5.2472305	2.4810162	1960.000	0.4510204
mass	-0.4916718	-2.8543765	1.7051067	1960.000	0.6602041

Table S39: Posterior distributions of the parameter estimates of the gaussian bayesian comparative analysis that includes swimmers.

S5.4 Walkers MCMC

```r
library(MCMCglmm)

W_MCMC_inv <- MCMCglmm(PC1 ~ Lat + Mig_bi + mass,
                       data=W_MCDs, family="gaussian",
                       random=~animal, ginverse=list(animal=inverseA(treeW)$Ainv),
                       prior=list(R=list(V=diag(1),nu=0.002),
                                  G=list(G1=list(V=diag(1), nu=0.002))),
                       pl=TRUE, nitt=5000000,thin=2500,burnin=100000, verbose =TRUE)
```

```r
# Phylogenetic variance
mean(W_MCMC_inv$VCV[, "animal"])
```

```
## [1] 414.2489
```

```r
# Heritability
mean(W_MCMC_inv$VCV[, "animal"]/(W_MCMC_inv$VCV[, "animal"] + W_MCMC_inv$VCV[, "units"]))
```

```
## [1] 0.9994667
```

```r
# Gelman and Rubin's convergence diagnostic
gelman.diag(mcmc.list(W_MCMC_inv$Sol, W_MCMC_inv1$Sol))$mpsrf
```

```
## [1] 1.005663
```

	post.mean	l.95..CI	u.95..CI	eff.samp	pMCMC
(Intercept)	-2.1289826	-23.8209009	15.7944629	1807.164	0.8357143
Lat	0.0129916	-0.0291734	0.0514986	2122.358	0.5459184
Mig_bi	3.3710156	0.6031443	5.8205425	1960.000	0.0081633
mass	0.1851607	-0.4416597	0.7768771	1960.000	0.5500000

Table S40: Posterior distributions of the parameter estimates of the gaussian bayesian comparative analysis that includes walkers.

Appendix S6: Multinomial bayesian comparative analyses

```r
library(MCMCglmm)

F_MCDsM <- F_MCD[,c(1,6,14,16,23,24,10)]
Multi_MCMC <- MCMCglmm(Mig_multi ~ Lat + PC1 + PC2 + mass,
                       data=F_MCDsM, family="ordinal",
                       random=~animal, ginverse=list(animal=inverseA(tree)$Ainv),
                       prior=list(R=list(V=1, fix=1),
                                  G=list(G1=list(V=1, nu=1000, alpha.mu=0, alpha.V=1))),
                       pl=TRUE, nitt=5000000,thin=2500,burnin=100000, verbose =TRUE)
```

```r
# Phylogenetic variance
mean(Multi_MCMC$VCV[, "animal"])
```

```
## [1] 16.72316
```

```r
# Heritability
mean(Multi_MCMC$VCV[, "animal"]/(Multi_MCMC$VCV[, "animal"] + Multi_MCMC$VCV[, "units"] + 1))
```

```
## [1] 0.8894602
```

```r
# Gelman and Rubin's convergence diagnostic
gelman.diag(mcmc.list(Multi_MCMC$Sol, Multi_MCMC1$Sol))$mpsrf
```

```
## [1] 1.001057
```

	post.mean	l.95..CI	u.95..CI	eff.samp	pMCMC
(Intercept)	-0.6530587	-6.0537667	4.0979565	1960.000	0.8091837
Lat	0.0880901	0.0729894	0.1031288	1960.000	0.0005102
PC1	0.0251586	0.0021071	0.0464791	2110.727	0.0204082
PC2	-0.0265516	-0.0646662	0.0088995	1960.000	0.1387755
mass	-0.5110345	-0.7396367	-0.3129918	1800.783	0.0005102

Table S41: Posterior distributions of the parameter estimates of the multinomial bayesian comparative analysis that includes flyers.
