# Supplementary material for: Migrant birds and mammals live faster than residents
Source: Nat Commun. 2020 Nov 17;11:5719. doi: 10.1038/s41467-020-19256-0 (PMC7673136; doi:10.1038/s41467-020-19256-0)
Supplement: Supplementary file 3 — Reporting Summary [file 41467_2020_19256_MOESM3_ESM.pdf]

nature research

Corresponding author(s): Stuart Bearhop, David Hodgson

Last updated by author(s): Oct 6, 2020

Reporting Summary

Nature Research wishes to improve the reproducibility of the work that we publish. This form provides structure for consistency and transparency in reporting. For further information on Nature Research policies, see Authors & Referees and the Editorial Policy Checklist .

Statistics

For all statistical analyses, confirm that the following items are present in the figure legend, table legend, main text, or Methods section.

n/a	Confirmed	
☐	☒	The exact sample size (*n*) for each experimental group/condition, given as a discrete number and unit of measurement
☒	☐	A statement on whether measurements were taken from distinct samples or whether the same sample was measured repeatedly
☐	☒	The statistical test(s) used AND whether they are one- or two-sided<br>*Only common tests should be described solely by name; describe more complex techniques in the Methods section.*
☐	☒	A description of all covariates tested
☐	☒	A description of any assumptions or corrections, such as tests of normality and adjustment for multiple comparisons
☐	☒	A full description of the statistical parameters including central tendency (e.g. means) or other basic estimates (e.g. regression coefficient) AND variation (e.g. standard deviation) or associated estimates of uncertainty (e.g. confidence intervals)
☐	☒	For null hypothesis testing, the test statistic (e.g. *F*, *t*, *r*) with confidence intervals, effect sizes, degrees of freedom and *P* value noted<br>*Give P values as exact values whenever suitable.*
☐	☒	For Bayesian analysis, information on the choice of priors and Markov chain Monte Carlo settings
☐	☒	For hierarchical and complex designs, identification of the appropriate level for tests and full reporting of outcomes
☐	☒	Estimates of effect sizes (e.g. Cohen's *d*, Pearson's *r*), indicating how they were calculated

Our web collection on statistics for biologists contains articles on many of the points above.

Software and code

Policy information about availability of computer code

Data collection	No software was used to collect the data
Data analysis	Basic analyses were carried out using program R. Details of the phylogenetic analyses and associated code are provided with the supporting information

For manuscripts utilizing custom algorithms or software that are central to the research but not yet described in published literature, software must be made available to editors/reviewers. We strongly encourage code deposition in a community repository (e.g. GitHub). See the Nature Research guidelines for submitting code & software for further information.

Data

Policy information about availability of data

All manuscripts must include a data availability statement. This statement should provide the following information, where applicable:

- Accession codes, unique identifiers, or web links for publicly available datasets
- A list of figures that have associated raw data
- A description of any restrictions on data availability

Data were harvested from online resources and multiple published papers (these are detailed in the supporting information document). Data supporting the analyses will be housed in Dryad (stated in the supporting information document) prior to any publication and full accession details will be provided.

Field-specific reporting

Please select the one below that is the best fit for your research. If you are not sure, read the appropriate sections before making your selection.

☐ Life sciences ☐ Behavioural & social sciences ☒ Ecological, evolutionary & environmental sciences

Ecological, evolutionary & environmental sciences study design

All studies must disclose on these points even when the disclosure is negative.

Study description	All qualitative measures (e.g. migrant, non-migrant, partial migrant) were based on categorization by other experts in the field (i.e. the animals were already described as thus in the sources the data were harvested from, or in alternative sources covering the species in question). More detail is given in the supporting information document
Research sample	Overall sample sizes were dictated by: 1) presence of the species on the amniotes database and 2) whether there were associated data on the life history traits of interest.
Sampling strategy	Sampling size was determined as outlined above (species with the appropriate data that appear on the amniotes database).
Data collection	Data were harvested by A. Soriano Redondo and Jorge S. Gutiérrez.
Timing and spatial scale	NA
Data exclusions	Data on reptiles were excluded as they were only represented by a single taxon, all of which are migratory (marine turtles). This is outlined in the MS.
Reproducibility	Not experimental, but the the same analyses as used for the entire dataset were conducted for the individual groups and the overall patterns remained the same (detailed in the MS).
Randomization	This is a species-level analysis and thus does not feature data from individual animals. It is not experimental and so allocation to groups was based on life histories and other species-level traits.
Blinding	As above this was not an experimental piece of work and blinding was not necessary. All species were allocated to their respective groups a priori, based on published information about their ecologies and life histories

Did the study involve field work? ☐ Yes ☒ No

Reporting for specific materials, systems and methods

We require information from authors about some types of materials, experimental systems and methods used in many studies. Here, indicate whether each material, system or method listed is relevant to your study. If you are not sure if a list item applies to your research, read the appropriate section before selecting a response.

Materials & experimental systems

n/a	Involved in the study	
☒	☐	Antibodies
☒	☐	Eukaryotic cell lines
☒	☐	Palaeontology
☐	☒	Animals and other organisms
☒	☐	Human research participants
☒	☐	Clinical data

Methods

n/a	Involved in the study	
☒	☐	ChIP-seq
☒	☐	Flow cytometry
☒	☐	MRI-based neuroimaging

Animals and other organisms

Policy information about studies involving animals; ARRIVE guidelines recommended for reporting animal research

Laboratory animals	The study did not involve laboratory animals
Wild animals	This is a meta-analyses of data already collected by others. It involves over 1200 species of wild mammal and bird and thus there is no real way of detailing how each part of the data set was collected
Field-collected samples	See our comments above
Ethics oversight	No ethical approval was needed as the study was a meta-analysis based on data that had already been published

Note that full information on the approval of the study protocol must also be provided in the manuscript.
