# Supplementary material for: Migrant birds and mammals live faster than residents
Source: Nat Commun. 2020 Nov 17;11:5719. doi: 10.1038/s41467-020-19256-0 (PMC7673136; doi:10.1038/s41467-020-19256-0)
Supplement: Supplementary file 2 — Peer Review File [file 41467_2020_19256_MOESM2_ESM.pdf]

Reviewer #1 (Remarks to the Author):

The Introduction could perhaps be shortened up to line 69, especially since some of the generic but ‘equivocal’ contrasts (e.g. higher survival, line 58; telomere relationship, line 59) appear to be based on single, empirical studies [27,28] which don’t really constitute generalizable life-history patterns. The section starting line 70 is much better written, generating predictions from first principles rather than through review of specific studies.
Line 97. I agree that predictions “are difficult” (although the costs of transport ideas are cool and pretty explicit) but I think they are not essential here: using a large, powerful data base for this type of analysis, even if “exploratory” is, I think, inherently valuable. However, I wonder about the effect (circularity) or starting with the assumption that there ARE “costs” of migration (line 98). Aren’t some studies at least consistent with the idea that migration might be cost-free, e.g. no difference in survival; costs coming from ‘non-migratory’ parts of migration such as stop-over activities. What would this (radical) line of thought do to your predictions, and interpretation of results?
It seems that your six LH traits have a “pace of life” focus, but this idea doesn’t emerge in the Introduction. Should it? Is this a useful framework for your predictions? (even though this is addressed on line 120). Data on longevity can be problematic (if this is maximum lifespan, often obtained in captivity). Could you have used estimates of annual (adult) survival?
A general comment I always have with this type of phylogenetic comparative analysis: this study does a reasonable job of identifying broad “predictors” of migratory propensity (body size, POLS) but how much of the variation is explained by these traits? Even for LH traits, the first PC explains ‘only’ 46% and 29% of the overall variation present in the data set. I often feel that the more interesting story is the biological variation around any line for a main predictor. Are there particular groups of species or sets of LH traits that buck the main trend(s)? There is nothing in the Discussion addressing this issue.

Line 189. Why “might”? Either your results did, or did not, support this.

Line 196. See my comments above about “costs”. Is there good evidence that migrants actually have a “hard working life-style” compared with residents, when prorated over the compete annual cycle?

Line 207-214. I understand the rationale for results for walkers/swimmers here. But the explanation for the different result in flyers was not so clear. You simply state that MR, speed and size are related (based on other studies). Does body size affect a species’ ability to store fuel (fat) for migratory flight? Is this consistent with the pattern you describe? I’m wondering if I couldn’t come up with a post-hoc explanation for the opposite effect?

Discussion, longer and more speculative than it needs to be, especially given empirical results. I appreciate that reference to climate change is timely/sexy but its not clear to me why (or how) large species of fast POLS species would be more “threatened”. For example, higher reproductive rates in fast POLS species could allow a more rapid evolutionary response to climate change.

Line 285. As I suggested above, we have encountered some problems with using longevity (max lifespan) from these same databases. Survival estimates might be better in terms of mortality?

In Fig 2 the legend does not explain the color coding on bar plots (this is consistent throughout but I couldn’t see an explanation anywhere).

Reviewer #2 (Remarks to the Author):

The authors address an interesting question that is not well resolved, as they point out. They are to be applauded for attempting to conduct a broad analysis across many species.

At the same time, the breadth of the taxa made it difficult to fully assess results and patterns. The use of phylogenetic analyses is appropriate and important, but it still does not fully account for phylogenetic effects. The lambdas near 1 demonstrate that traits are ¬_very_ highly confounded with phylogeny and suggest some separate analyses are needed. Mammals show a particularly large phylogenetic confounding with respect to migration (Fig. 1). How many birds are 'walkers'? Is this largely just mammals? As such, I would like to see separate analyses of a few orders or taxa groups that are well represented with respect to migration and non-migration. For example, maybe pull out Passeriformes, Chiroptera, ungulates, oceanic non-passerines. I suggest that you analyze mammals and birds separately overall. You can show that they show the same patterns if true, just as you did with locomotor types.

In addition, we are given no sense of taxonomic representation among locomotor classes and with respect to migration. I would like to see the list of species that are included and their migratory status. Graphs should differentiate migrant vs non-migrant (e.g., open vs closed symbols) and mammal vs birds (different symbols – circles vs squares for example).

Because there is so much confounding, it is difficult to assess the validity of results and there are issues that concern me. Figure 3E show that migration is strongly confounded with latitude. We know that life history strategies are slower at the tropics than temperate, so I worry that this is confusing results (slower life history of resident tropical spp cause non-migration to seem to have slow life histories) even though latitude is included in the model. I would like to see LH of migrants vs nonmigrants in the tropics to demonstrate the purported life history differences. When I think about passerines, the group I know best, I think about the fastest life histories are in northern resident species, like tits, goldcrests rather than migrants. This is opposite the pattern the authors argue. Some groups of oceanic non-passerines show higher fecundity in the tropics than far north, but migration in the north. The lack of specific taxonomic comparisons of migrant vs non-migrant inhibits intuitive understanding of patterns.

I am concerned about the PCA. The authors state that they are using axes with eigenvalues >1. This is a 'rule of thumb' but does not necessarily represent statistical justification. The first PC axis only accounts for 28% of the variation. With only 7 traits, this suggests that traits do not strongly covary. PC2 accounts for 17% of the raw variation, which is 24.5% of the residual variation, almost the same as the 28% of PC1. One of the concerns with PCA is that axes effectively describe a sphere such that they are not unique or appropriate solutions. Bartlett's test of sphericity should be applied. But the low variation explained by PC1 argues against it being taken to simply represent the slow-fast life history gradient as the authors argue. PC2 doesn't seem to come out as important in analyses with beta confidence intervals including 0 (Tables 1, 2). In addition, the two traits the authors argue this axis represents (reproductive episodes and number of young) are also argued as important for PC1, plus th other 5 traits are still included in PC2 making it much less clear than the authors suggest. With only 7 traits and large sample sizes, I suggest that models should include direct analyses of the traits rather than PC axes.

The authors treat latitude as an absolute value, where northern and southern latitudes are treated equally. Various studies of birds have long shown that life histories do not vary the same with latitude in the north vs south. Moreau (1944) pointed out long ago that many south temperate species have the same clutch sizes as tropical relatives, whereas north temperate are much larger (also Yom-Tov et al. 1994 Condor and others). Several studies have also shown that adult survival in south temperate locations do not necessarily differ from tropical, whereas north temperate are lower (e.g., Yom-Tov et al 1992 Ibis, Lloyd et al 2014 J Av Biol). Migration also tends to be less common in the south temperate than north temperate. Latitude should be treated as true latitude, not absolute values.

Number of reproductive episodes and offspring number are largely count data. As such, log-transformation is not a good approach. You should use should use square root, quasi-poisson or negative-binomial model approaches.

Some of the size relationships look odd. Prenatal development has a very large group at the large end that are all high outliers – this presumably reflects overdominance of smaller species driving the relationship, but suggests that the size correction is not appropriate and yields inappropriate residuals at the large end. The same is true for offspring number – it looks like no relationship below 8 and the forcing of a slope by larger species again causes inappropriate residuals among small species.

Lines 45-47: It is difficult to be comprehensive given limited space, but some acknowledgement of all the work that Levey has done on the evolution of migration and resources should be included (see Am Nat 1992 and subsequent) as well the more recent papers with Jahn.

Line 58 – this reminds me of an old paper by Martin (1993 BioScience) where he showed that nest type was a major confound of migration and survival in north temperate, whereby migrants had higher survival than residents, but residents were largely hole-nesters. When compared within a nest type, then survival did not differ among migration categories. Naturally this was a small sample size, but Jetz et al. (ref 29) also found a strong nest type effect on clutch sizes in a massive test across the world. It seems nest type should be included for birds. At the same time, this kind of confounding factor is what worries me about this massive cross-taxa set.

Line 66 – The authors say that tropical species have faster life histories, an error.

Lines 97-99 – this is very confusing. Migration has costs – do you mean survival costs? Or energetic costs? But we know that species that overwinter in the north also incur both survival and energetic costs. Which is more costs – northern winters or migration? Second, the authors argue that migrants might compensate for these costs by being "highly productive with long reproductive lifespans". This sounds like you are arguing they are approaching Darwinian demons. This does not fit with known patterns or life history theory and needs clarification.

Line 103: the authors state: "we hypothesise that certain traits should differ between partial and obligate migrants". This is extremely vague. Which traits and differ how?

In summary, the goal of this study is admirable and important. The approach to test across both mammals and birds is also admirable and impressive. But… the potential for additional confounding is huge. Some added analyses of some specific taxa to demonstrate the patterns occur repeatedly within latitudinal regions are needed to allow intuitive understanding of the biology of patterns.

Reviewer #3 (Remarks to the Author):

In this paper, the authors investigate the existence of a relationship between migratory behaviour and life history traits in birds and mammals. This is a well-written paper on an interesting topic. The authors convincingly show that the literature is unclear on the subject and that this study is a valuable contribution. I generally like the study and would recommend it for publication in Nature Communications. I nevertheless have a few comments, detailed below, which I think should be addressed.

The analysis reveals a relationship between migratory propensity and the pace of life, but I cannot quite work out whether it can definitely rule out the possibility that this relationship could just be a by-product of body mass. As in, for flying species for example, the authors found a positive relationship between migratory propensity and the pace of life (e.g. Fig 4d), a negative relationship between migratory propensity and body mass (e.g. Fig 4a) and a negative relationship between body mass and the pace of life (Fig S2). Body mass could therefore directly affect migratory propensity because of known biomechanical and energetic constraints (e.g. larger flying species have more energetic

problems for migrating than smaller species) and directly affect the pace of life (i.e. this is one of the main results of the metabolic theory of ecology), and therefore as an indirect by-product there is a relationship between migratory propensity and the pace of life. I might be missing it, but can the analysis rule out this possibility? And if so, I think it should be clearer in the text.
For walking and swimming species, the authors found a positive relationship between body mass and migratory propensity, which contrasts with flying species, but does Figure 4d holds for each separate locomotion mode?

Is it possible that the positive relationship between body mass and migratory propensity for walking and swimming species is an artefact of data availability? Movement data are more easily recorded for larger species, particularly in mammals, which are harder to observe than birds (particularly small mammals). Therefore, is it possible that the low migratory propensity for smaller walking and swimming species reflects the fact that we simply don't know much about the movement of these species? It would be good to have a discussion of this potential limitation. Or if it is not a problem in this dataset, explaining why.

Fig 1 appears to indicate that more than half of the bird species included in the analysis are migratory. However, in the dataset used by the authors for defining migratory behaviour (Eyres et al. 2017), only 15% are migratory (directional migration). I therefore wonder whether the data is truly representative of birds in general or whether there is a bias towards a highly migratory subset of the avifauna, which could potentially bias the analysis of migratory propensity. It would be good to clarify that.

L99–102: Why? I would be good to describe here the rational for this prediction.

L161: mass is repeated twice.

Reviewer #4 (Remarks to the Author):

The paper "Migrant birds and mammals live faster than residents", is a large scale comparative analysis of the relationship between the presence of migration in vertebrates and their life history strategies. I really enjoyed reading the paper and I think the question and analysis are interesting and it will be an important contribution to the field. Overall, I think this is an excellent manuscript that requires relatively minor changes. Mainly I think the approach to the analysis is appropriate overall, however, I do a some comments regarding some aspects of the analysis.

Comments.

Line 285: While I assume that the six life history traits were chosen based on their availability in the amniotes database and to cover the various axis of life history strategies. However, this reasons behind these choices should be made clearer. In particular, it would be useful to mentioned which traits are expected to represent the fast-slow continuum and which traits are expected to represent other axis of variation in life history strategies (and also what these axis of variation may be in general). I think this is important in terms of interpreting the PCA analysis.

Line 295: Why was the centroid of the breeding site used for migratory birds but not for mammals?

Line 304: I assume only one random tree was used and not the entire distribution based on the available code. Either this should be made clearer or the full distribution of trees should be used.

Line 314: I assume body mass was also log transformed. If so it should be made clearer here.

Were the MCMCglmm models tested for convergence? It is not mentioned in the methods section or

present in the code. I would expect at least 2-3 chains run for each model and checked for convergence using something like the Gelman-Rubin metric. The priors for the random terms also need to be given.

Why was MCMCglmm not used for the initial mass correction models. This is only a very minor point as it won't make a difference to the results but it is unusual to mix and match the two approaches without any clear reason.

The effect size for PC1 on whether a species is migratory or not looks small (0.04, Table 1), however I note that the span for PC1 is approximately 100 units. I think it would be really helpful to frame this effect size in some more accessible terms. For example, how far across the full PC1 axis would you need to move to increase the probability of a species being migratory by 0.5, and how does that effect size compare to the importance of body size and locomotion type?

The tables using MCMCglmm models should include the residual variance terms. Giving this info can be important, for example the ratio between the phylogenetic and other variance terms can be used to calculate the equivalent of Pagels lambda (See Hadfield, J. & Nakagawa, S. (2010). General quantitative genetic methods for comparative biology: phylogenies, taxonomies and multi-trait models for continuous and categorical characters. J. Evol. Biol., 23, 494–508.)

REVIEWER COMMENTS

Reviewer #1 (Remarks to the Author):

"The Introduction could perhaps be shortened up to line 69, especially since some of the generic but 'equivocal' contrasts (e.g. higher survival, line 58; telomere relationship, line 59) appear to be based on single, empirical studies [27,28] which don't really constitute generalizable life-history patterns. The section starting line 70 is much better written, generating predictions from first principles rather than through review of specific studies"

Response: This is a fair point and we have shortened the introduction accordingly. In summary, we have deleted statements where we: (i) mentioned the flexibility of migratory behaviour as evidence largely comes from within-species studies; and (ii) we argued the potential utility of such a global and integrative understanding of the drivers of migration as this is discussed later on (Discussion section).

"Line 97. I agree that predictions "are difficult" (although the costs of transport ideas are cool and pretty explicit) but I think they are not essential here: using a large, powerful data base for this type of analysis, even if "exploratory" is, I think, inherently valuable. However, I wonder about the effect (circularity) or starting with the assumption that there ARE "costs" of migration (line 98). Aren't some studies at least consistent with the idea that migration might be cost-free, e.g. no difference in survival; costs coming from 'non-migratory' parts of migration such as stop-over activities. What would this (radical) line of thought do to your predictions, and interpretation of results?"

Response: We have rephrased this and it now reads: "Predictions are difficult to generate given the contradictory evidence base, but we propose that migration has costs in terms of survival and that migrants should have life history responses that allow them to compensate by having higher reproductive output" (Lines **96-99**). The cost-free idea is an interesting one and we agree that the "migration as a costly life style" idea is much minsinterpreted. As we outline in the MS in the context of life history trade-offs, long distance migration uses energy that cannot subsequently be allocated to self-maintenance or reproduction and this is where we would view much of the 'cost' coming from. We have rephrased the narrative around this and hope that it is now clearer.

"It seems that your six LH traits have a "pace of life" focus, but this idea doesn't emerge in the Introduction. Should it? Is this a useful framework for your predictions? (even though this is addressed on line 120). Data on longevity can be problematic (if this is maximum lifespan, often obtained in captivity). Could you have used estimates of annual (adult) survival?"

Response: This is a helpful point. We originally hoped that it was implicit from the title and the in language used. We have now made the pace of life syndrome perspective a feature from the outset. This has strengthened the narrative and it now sits much more comfortably within the predictive framework (Lines **63-68**).

With respect to the potential issues linked to longevity data, it is important to note that we used "longevity" (i.e. the median of the reported longevity data for a particular species), and not "maximum longevity" (i.e. maximum recorded longevity for species). This is reported in the original paper, but we have now made this much clearer (Lines **308-309**). We chose to use the former because, as the referee points out, the maximum values represent extremes (particularly when this comes from captive individuals) and are sample size dependant. We would have been very keen to use adult survival rather than longevity, but this would have severely constrained the data available to us as there are far less species that have reliable estimates of this parameter.

"A general comment I always have with this type of phylogenetic comparative analysis: this study does a reasonable job of identifying broad "predictors" of migratory propensity (body size, POLS) but how much of the variation is explained by these traits? Even for LH traits, the first PC explains 'only' 46% and 29% of the overall variation present in the data set. I often feel that the more interesting story is the biological variation around any line for a main predictor. Are there particular groups of species or sets of LH traits that buck the main trend(s)? There is nothing in the Discussion addressing this issue."

Response: We now perform separate models for orders that have a larger sample size (>20 species) and have a balanced number of migratory and non-migratory species (> 25% in the least represented strategy). Those criteria are met in Passeriformes, Artiodactyla, Accipitriformes, Pelecaniformes and Chiroptera. Given the strong results for Passeriformes only, we also re-run flyers and birds without this class and find the relationships to persist. We provide the results from the models in the Results section and Supporting Information and discuss the findings in the Discussion.

"Line 189. Why "might"? Either your results did, or did not, support this."

Response: We deleted "might".

"Line 196. See my comments above about "costs". Is there good evidence that migrants actually have a "hard working life-style" compared with residents, when prorated over the compete annual cycle?"

Response: We now cover this point in the discussion (see also comments above on costs): "However, the idea that increased energetic output reduces lifespan remains controversial. For example, high output in one part of the annual cycle can be compensated by lowering output in others, which points

to a need for comparative studies to account for the energetic costs throughout annual cycle" (Lines **211-214**).

"Line 207-214. I understand the rationale for results for walkers/swimmers here. But the explanation for the different result in flyers was not so clear. You simply state that MR, speed and size are related (based on other studies). Does body size affect a species' ability to store fuel (fat) for migratory flight? Is this consistent with the pattern you describe? I'm wondering if I couldn't come up with a post-hoc explanation for the opposite effect?"

Response: This is an interesting point. Indeed, fuel deposition rates do vary among and within-species, and body mass determines the rate of fuelling at least in part (Linström 2003: Fuel Deposition Rates in Migrating Birds: Causes, Constraints and Consequences, in *Avian Migration*). We now cover this as follows: "Among flyers, fuel deposition rate is negatively correlated with body mass. Combined with the negative relationship between speed and body size this will limit the ability to store fuel stores in large species. As such, large migratory birds face larger energetic problems than smaller birds when forced to use flapping flight" (Lines **230-233**).

"Discussion, longer and more speculative than it needs to be, especially given empirical results. I appreciate that reference to climate change is timely/sexy but its not clear to me why (or how) large species of fast POLS species would be more "threatened". For example, higher reproductive rates in fast POLS species could allow a more rapid evolutionary response to climate change."

Response: We have trimmed the discussion and some of the speculation. We acknowledge the referees concerns about jumping on bandwagons by raising the spectre of climate change. However, the relationship between body size and extinction risk is well established. Given that large body size fast POL and migratory behaviour are linked in walkers and swimmers, the previous suggestion seems reasonable. Further, there is a huge body of evidence from birds that migratory behaviour is related to population decline and extinction risk. However, have altered the text around climate change and its potential to interact with POL.

"Line 285. As I suggested above, we have encountered some problems with using longevity (max lifespan) from these same databases. Survival estimates might be better in terms of mortality?"

Response: As noted above, we used median longevity instead of maximum longevity to avoid extreme values. Ideally, we would have used estimates of adult survival, but as explained above, these are not reported as often as longevity estimates and using them would have compromised the comprehensive nature of our analyses.

"In Fig 2 the legend does not explain the color coding on bar plots (this is consistent throughout but I couldn't see an explanation anywhere)."

Response: The colour code was included in the legend inside the scatterplot, however we acknowledge that this was not clear enough and have added text and moved the legend to the bottom of the plot.

Reviewer #2 (Remarks to the Author):

"The authors address an interesting question that is not well resolved, as they point out. They are to be applauded for attempting to conduct a broad analysis across many species.

At the same time, the breadth of the taxa made it difficult to fully assess results and patterns. The use of phylogenetic analyses is appropriate and important, but it still does not fully account for phylogenetic effects. The lambdas near 1 demonstrate that traits are very highly confounded with phylogeny and suggest some separate analyses are needed. Mammals show a particularly large phylogenetic confounding with respect to migration (Fig. 1). How many birds are 'walkers'? Is this largely just mammals? As such, I would like to see separate analyses of a few orders or taxa groups that are well represented with respect to migration and non-migration. For example, maybe pull out Passeriformes, Chiroptera, ungulates, oceanic non-passerines. I suggest that you analyze mammals and birds separately overall. You can show that they show the same patterns if true, just as you did with locomotor types."

Response: We appreciate these positive and constructive comments. We now clarify that walkers are mammals (Lines **324-326**). As suggested, we have now added separate analyses for mammals and birds, as well as for orders with >20 species and a balanced number of migratory and non-migratory species (> 25% in the least represented strategy), in addition to the analyses that include all taxa. Those groups are Passeriformes, non-Passeriform flyers, non-Passeriform birds, Artiodactyla, Accipitriformes, Pelecaniformes and Chiroptera. The birds appear to be most robust in this respect with relationships persisting in Passeriformes, non-Passeriform flyers, non-Passeriform birds. However, it is also clear from this that no one group of mammals is driving the relationships for walkers and swimmers. We provide the models and results from the model in the Results section and Supporting Information and discuss the findings in the Discussion.

"In addition, we are given no sense of taxonomic representation among locomotor classes and with respect to migration. I would like to see the list of species that are included and their migratory status. Graphs should differentiate migrant vs non-migrant (e.g., open vs closed symbols) and mammal vs birds (different symbols – circles vs squares for example)."

Response: We now include Table S1 in Supporting Information that summarises the number of non-migratory and migratory species in each order. Following the suggestion of the reviewer the graphs now differentiate between non-migratory (squares) and migratory species (circles).

"Because there is so much confounding, it is difficult to assess the validity of results and there are issues that concern me. Figure 3E show that migration is strongly confounded with latitude. We know that life history strategies are slower at the tropics than temperate, so I worry that this is confusing results (slower life history of resident tropical spp cause non-migration to seem to have slow life histories) even though latitude is included in the model. I would like to see LH of migrants vs nonmigrants in the tropics to demonstrate the purported life history differences. When I think about passerines, the group I know best, I think about the fastest life histories are in northern resident species, like tits, goldcrests rather than migrants. This is opposite the pattern the authors argue. Some groups of oceanic non-passerines show higher fecundity in the tropics than far north, but migration in the north. The lack of specific taxonomic comparisons of migrant vs non-migrant inhibits intuitive understanding of patterns."

Response: Unfortunately, our sample size of flyers and walkers in the tropics is fairly small, thus we have performed the suggested analysis for animals in temperate regions (n = 864) which includes species from 23.5ºN to 66.5 ºN and from 23.5ºS to 66.5 ºS. The patterns are equivalent to the full model. This result combined with the low numbers of topical flyers and walkers in our database indicates that the relationship between migratory strategy and pace of life is unlikely to be confounded by latitude. In addition, in the set of models where PC1 is the response variable the relationship between migration strategy and pace of life persists, which we believe reinforces the strength of our findings. Thus, the association between migratory tendency and PC1 is inferred to be over and above any influence of latitude on migratory tendency and influence of migration on PC1 is inferred to be over and above any influence of latitude on PC1. As pointed above, we now include taxon-specific analyses for Passeriformes, non-Passeriform flyers, non-Passeriform birds, Artiodactyla, Accipitriformes, Pelecaniformes and Chiroptera.

"I am concerned about the PCA. The authors state that they are using axes with eigenvalues >1. This is a 'rule of thumb' but does not necessarily represent statistical justification. The first PC axis only accounts for 28% of the variation. With only 7 traits, this suggests that traits do not strongly covary. PC2 accounts for 17% of the raw variation, which is 24.5% of the residual variation, almost the same as the 28% of PC1. One of the concerns with PCA is that axes effectively describe a sphere such that they are not unique or appropriate solutions. Bartlett's test of sphericity should be applied. But the low variation explained by PC1 argues against it being taken to simply represent the slow-fast life history gradient as the authors argue. PC2 doesn't seem to come out as important in analyses with beta

confidence intervals including 0 (Tables 1, 2). In addition, the two traits the authors argue this axis represents (reproductive episodes and number of young) are also argued as important for PC1, plus the other 5 traits are still included in PC2 making it much less clear than the authors suggest. With only 7 traits and large sample sizes, I suggest that models should include direct analyses of the traits rather than PC axes."

Response: We thank the reviewer for pointing this out. We have added detail from Bartlett's test of sphericity applied to assess the appropriateness of our use of PCA to reduce the dimensionality of the size-corrected residuals from the six life-history traits. The test indicates collinearity among the size-corrected residuals from the six life-history traits (χ^2= 2733.95; p < 0.001). This allows us to be confident that the PCA is an appropriate way to reduce the dimensions of the life history traits. We chose to keep PC1 and PC2 for subsequent models because both axes had eigenvalues > 1 and when the PCA was repeated for mammals and birds these two axes were equivalent for both taxonomic groups, while PC3 represented different life history components for birds and mammals. Moreover, Healy et al. 2019 Nat. Ecol. Evol. found a similar PCA structure for PC1 and PC2 highlighting the the robustness of these two axes. We did not find any significant relationship between migration strategy and PC2, we chose to keep this axis on the models because we wanted to test if there was a relationship between migration and reproduction strategy. Due to the collinearity among the size-corrected residuals from the six life-history traits running a model including all life history traits would not be appropriate.

"The authors treat latitude as an absolute value, where northern and southern latitudes are treated equally. Various studies of birds have long shown that life histories do not vary the same with latitude in the north vs south. Moreau (1944) pointed out long ago that many south temperate species have the same clutch sizes as tropical relatives, whereas north temperate are much larger (also Yom-Tov et al. 1994 Condor and others). Several studies have also shown that adult survival in south temperate locations do not necessarily differ from tropical, whereas north temperate are lower (e.g., Yom-Tov et al 1992 Ibis, Lloyd et al 2014 J Av Biol). Migration also tends to be less common in the south temperate than north temperate. Latitude should be treated as true latitude, not absolute values."

Response: Our sample size includes a relatively small number of southern temperate and austral species (< 25%), thus we think that using the absolute latitude would be more appropriate to correct for the latitudinal effect on migration. To assess the potential effect of transforming the latitudes to absolute values, we now include another model for walking and flying species occurring in the Northern Hemisphere (only positive latitudes) and the results are equivalent. Moreover, a pattern where high latitude species share life history traits with tropical species would be unlikely to confound our results and would more likely make the patterns we find more difficult to detect.

"Number of reproductive episodes and offspring number are largely count data. As such, log-transformation is not a good approach. You should use should use square root, quasi-poisson or negative-binomial model approaches."

Response: For the majority of cases in the database, the number of reproductive episodes and offspring number are decimal numbers. We tried several transformations (including those outlined above) but found that log-transformation was the most appropriate in terms of model-checking (homogeneity of variances and normality of residuals). Therefore, we have chosen to keep these variables log-transformed.

"Some of the size relationships look odd. Prenatal development has a very large group at the large end that are all high outliers – this presumably reflects overdominance of smaller species driving the relationship, but suggests that the size correction is not appropriate and yields inappropriate residuals at the large end. The same is true for offspring number – it looks like no relationship below 8 and the forcing of a slope by larger species again causes inappropriate residuals among small species."

Response: These slopes are forced by the phylogenetic component of the model. Phylogenetic models tend to "flatten" the relationships among variables, thus yielding larger residuals at the extremes of the range. When we perform the analyses using GLMs the residuals do not depart significantly from normality.

"Lines 45-47: It is difficult to be comprehensive given limited space, but some acknowledgement of all the work that Levey has done on the evolution of migration and resources should be included (see Am Nat 1992 and subsequent) as well the more recent papers with Jahn."

Response: We have now included these: (Levey and Stiles 1992 Am Nat) in order to reinforce the importance of seasonality of habitat and diet in driving seasonal movements of birds across geographical scales (Line **48**), and Faaborg… Jahn, et al. 2010 Ecol. Monogr. (Line **33**).

"Line 58 – this reminds me of an old paper by Martin (1993 BioScience) where he showed that nest type was a major confound of migration and survival in north temperate, whereby migrants had higher survival than residents, but residents were largely hole-nesters. When compared within a nest type, then survival did not differ among migration categories. Naturally this was a small sample size, but Jetz et al. (ref 29) also found a strong nest type effect on clutch sizes in a massive test across the world. It seems nest type should be included for birds. At the same time, this kind of confounding factor is what worries me about this massive cross-taxa set."

Response: This is an interesting point. Nest type is indeed another intrinsic determinant of clutch size and probably predation/survival. Thus, it might also affect life history traits. As showed in Jetz et al.

(2008), nest type is correlated with clutch size (cavity nesters tend to have later clutch sizes than open nesters), which is in turn is correlated with development mode. Thus, we believe that the life history traits we used (namely durations of pre- and post-natal development, number of annual reproductive events and number of offspring in each reproductive event) should capture the types interspecific variation in reproductive strategy outlined above (PC2). This also makes it much more straightforward to construct the global model as there is no equivalent trait for mammals.

"Line 66 – The authors say that tropical species have faster life histories, an error."

Response: Thanks for picking up this error. We replaced "faster" with "slower" (Line **62**).

"Lines 97-99 – this is very confusing. Migration has costs – do you mean survival costs? Or energetic costs? But we know that species that overwinter in the north also incur both survival and energetic costs. Which is more costs – northern winters or migration? Second, the authors argue that migrants might compensate for these costs by being "highly productive with long reproductive lifespans". This sounds like you are arguing they are approaching Darwinian demons. This does not fit with known patterns or life history theory and needs clarification."

Response: Thank you for pointing out this inconsistency, we have now rephrased it: "Predictions are difficult to generate given the contradictory evidence base, but we propose that migration has costs in terms of survival and that migrants should have life histories that allow them to compensate (e.g. by having higher reproductive output)" (Lines **96-99**).

"Line 103: the authors state: "we hypothesise that certain traits should differ between partial and obligate migrants". This is extremely vague. Which traits and differ how?"

Response: We have now rephrased it: "Finally, although all migrant flyers should share broad life history traits, we hypothesise that partial migrants should have life histories intermediate to residents and full migrants because of the more constrained lifestyle of the latter and the relative contribution of resident and migratory individuals into the life history estimates of partially migratory species." (Lines **102-106**).

"In summary, the goal of this study is admirable and important. The approach to test across both mammals and birds is also admirable and impressive. But… the potential for additional confounding is huge. Some added analyses of some specific taxa to demonstrate the patterns occur repeatedly within latitudinal regions are needed to allow intuitive understanding of the biology of patterns."

Response: We thank the reviewer for the honest assessment. We have now performed taxon-specific analyses and where sample sizes are larger enough the patterns from the global models persist. We acknowledge that comparative analysis might "miss" the functional predictors that actually drive

variation in the system, but this does not detract from the core message, that there is an association between pace of life and migration. We would welcome, and intend to perform, further research to infer the mechanisms that underly the association.

Reviewer #3 (Remarks to the Author):

"In this paper, the authors investigate the existence of a relationship between migratory behaviour and life history traits in birds and mammals. This is a well-written paper on an interesting topic. The authors convincingly show that the literature is unclear on the subject and that this study is a valuable contribution. I generally like the study and would recommend it for publication in Nature Communications. I nevertheless have a few comments, detailed below, which I think should be addressed."

Response: We thank the reviewer for the positive comments and thoughtful suggestions on how to improve the manuscript. Further details on the changes implemented to incorporate the feedback are provided below.

"The analysis reveals a relationship between migratory propensity and the pace of life, but I cannot quite work out whether it can definitely rule out the possibility that this relationship could just be a by-product of body mass. As in, for flying species for example, the authors found a positive relationship between migratory propensity and the pace of life (e.g. Fig 4d), a negative relationship between migratory propensity and body mass (e.g. Fig 4a) and a negative relationship between body mass and the pace of life (Fig S2). Body mass could therefore directly affect migratory propensity because of known biomechanical and energetic constraints (e.g. larger flying species have more energetic problems for migrating than smaller species) and directly affect the pace of life (i.e. this is one of the main results of the metabolic theory of ecology), and therefore as an indirect by-product there is a relationship between migratory propensity and the pace of life. I might be missing it, but can the analysis rule out this possibility? And if so, I think it should be clearer in the text."

Response: This is an important issue and we deal with it in the following manner. First, each of the six life history traits was regressed against mass. We then use the residuals of these in the PCA (e.g. mass-corrected longevity, etc). Therefore, the pace of life values that we use in the analyses are "mass-corrected pace of life" values. In fact, if we perform a PGLS model with PC1 as a response variable and mass as an explanatory variable we find that there is not a significant relationship between the two variables (t-value = 0.593 and p-value = 0.5530). We have tried to make this clearer by including additional text (Lines **334-341**).

"For walking and swimming species, the authors found a positive relationship between body mass and migratory propensity, which contrasts with flying species, but does Figure 4d holds for each separate locomotion mode?"

Response: Yes, in the three locomotion-specific analyses we find that mass has a negative effect on migration propensity for flyers (Estimate = -0.42; CI = -0.67/ -0.17; Table S22 of Supplementary Information) and a positive effect for walkers (Estimate = 0.74; CI = 0.36/ 1.15; Supporting Information). For swimmers the CI slightly overlap 0, however the trend is positive (Estimate = 0.37; CI = -0.05/ 0.78; Supporting information).

"Is it possible that the positive relationship between body mass and migratory propensity for walking and swimming species is an artefact of data availability? Movement data are more easily recorded for larger species, particularly in mammals, which are harder to observe than birds (particularly small mammals). Therefore, is it possible that the low migratory propensity for smaller walking and swimming species reflects the fact that we simply don't know much about the movement of these species? It would be good to have a discussion of this potential limitation. Or if it is not a problem in this dataset, explaining why."

Response: We used the most detailed and up to date source of information for mammals, the "Handbook of the mammals of the world", which includes all known information about species movements. In addition, we only included in the analyses species that had been extensively studied, since we needed complete information of their adult body mass, longevity, age of female sexual maturity, duration of prenatal development, duration of postnatal development, number of annual reproductive events and number of offspring in each reproductive event. Thus, while this is a possibility that can't be completely ruled out (especially for swimming species such as cetaceans) we think that this is unlikely to be a limitation for our study. We now discuss it in lines **260-262**.

"Fig 1 appears to indicate that more than half of the bird species included in the analysis are migratory. However, in the dataset used by the authors for defining migratory behaviour (Eyres et al. 2017), only 15% are migratory (directional migration). I therefore wonder whether the data is truly representative of birds in general or whether there is a bias towards a highly migratory subset of the avifauna, which could potentially bias the analysis of migratory propensity. It would be good to clarify that."

Response: The limiting factor when compiling this dataset was the availability of estimates of the life history traits we required. Therefore, it is very likely that not all groups are equally represented. We now include this potential limitation in the discussion section (Lines **260-262**). However, it is also clear fromt the additional analyses we have carried out in response to concerns rasied by other referees, that there are no particularly speciose groups that appear to be driving the patterns we observe.

"L99–102: Why? I would be good to describe here the rational for this prediction."

Response: In this prediction we were not only referring to pace of life, but to body mass as well. The biomechanical models mentioned in the introduction show that body mass is likely to play a different role on migration propensity depending on the locomotion mode. We have now rephrased the sentence: "We also predict that life-history correlates of migration will depend on locomotory modes, such that walking, swimming and flying migrant species will have different life histories compared to their non-migrant relatives as a consequence of biomechanical constraints" (Lines **99-102**).

"L161: mass is repeated twice."

Response: Deleted, thank you for picking this up.

Reviewer #4 (Remarks to the Author):

"The paper "Migrant birds and mammals live faster than residents", is a large scale comparative analysis of the relationship between the presence of migration in vertebrates and their life history strategies. I really enjoyed reading the paper and I think the question and analysis are interesting and it will be an important contribution to the field. Overall, I think this is an excellent manuscript that requires relatively minor changes. Mainly I think the approach to the analysis is appropriate overall, however, I do a some comments regarding some aspects of the analysis."

Response: We are very grateful to the reviewer for the positive and supportive assessment of our manuscript. We have addressed all of the reviewer's comments, for which we provide detailed responses below.

Comments.

"Line 285: While I assume that the six life history traits were chosen based on their availability in the amniotes database and to cover the various axis of life history strategies. However, this reasons behind these choices should be made clearer. In particular, it would be useful to mentioned which traits are expected to represent the fast-slow continuum and which traits are expected to represent other axis of variation in life history strategies (and also what these axis of variation may be in general). I think this is important in terms of interpreting the PCA analysis."

Response: The reviewer is correct, the "Amniotes database" does provide estimates of many life history traits, but unfortunately, these data are incomplete with numerous NAs. We now clarify this in the methods section: "We selected these traits to reflect the life history components of interest, while at the same time maximising the number of species with a complete set. We expected a clear trade-off between long development times (age of female sexual maturity, duration of prenatal development, duration of postnatal development) and number of offspring per year" (Lines **307-311**).

"Line 295: Why was the centroid of the breeding site used for migratory birds but not for mammals?"

Response: We extracted the centroid of the breeding site from the distribution maps of the IUCN and Birdlife International. For birds those maps include separate breeding and non-breeding areas, however, for mammals it only includes the overall range. Therefore, it was not possible to extract the centroids of the breeding range for mammals. We now clarify this in the MS: "The geographic distribution of each species was extracted from the IUCN Red List of Threatened Species. For birds, latitude was calculated as the centroid of the species range for non-migratory species or the centroid of the breeding range for migratory species. For mammals, only the overall species range was available, thus, latitude was calculated as the centroid of the overall range". (Lines **319-323**).

"Line 304: I assume only one random tree was used and not the entire distribution based on the available code. Either this should be made clearer or the full distribution of trees should be used."

Response: We used a single consensus tree. We now make it clear in the text: "We constructed a single species-level phylogenetic consensus tree…" and " As we needed a phylogenetic tree containing" (Lines **327-331**).

"Line 314: I assume body mass was also log transformed. If so it should be made clearer here."

Response: Yes, body mass was also log transformed. We make it clear now: "Life-history traits were log-transformed (including mass) prior to…" (Lines **340-341**).

"Were the MCMCglmm models tested for convergence? It is not mentioned in the methods section or present in the code. I would expect at least 2-3 chains run for each model and checked for convergence using something like the Gelman-Rubin metric. The priors for the random terms also need to be given."

Response: We thank the reviewer for raising this issue and we have corrected this oversight. We ran two chains for each model. We now include the Gelman and Rubin's convergence diagnostic in for each model in Supplementary Information. The models converged in all cases (< 1.1). The priors of the random terms are specified for each model in the Supporting Information.

"Why was MCMCglmm not used for the initial mass correction models. This is only a very minor point as it won't make a difference to the results but it is unusual to mix and match the two approaches without any clear reason."

Response: We concur with the reviewer that there is no justification for running PGLS instead of MCMCglmm and that it is indeed a bit unorthodox. We originally used PGLS for the mass correction to speed up the modelling process since PGLS take a few second to run while similar MCMCglmm can take a few hours. However, we now use MCMCglmm for the mass correction (all the code and plots

are in Supplementary Information). As pointed out by the reviewer, the results are equivalent and it does not significantly change the PCAs.

"The effect size for PC1 on whether a species is migratory or not looks small (0.04, Table 1), however I note that the span for PC1 is approximately 100 units. I think it would be really helpful to frame this effect size in some more accessible terms. For example, how far across the full PC1 axis would you need to move to increase the probability of a species being migratory by 0.5, and how does that effect size compare to the importance of body size and locomotion type?"

Response: Since we are using a probit model the propensity to migrate does not increase linearly with pace of life; species with a very slow pace of life would need to change pace substantially before the propensity to migrate increases, while species in the middle range, will only need to change pace a bit to increase substantially migratory propensity. As such, we believe that Figure 3d provides an accurate picture of the how migration propensity changes with pace of life according to our models.

"The tables using MCMCglmm models should include the residual variance terms. Giving this info can be important, for example the ratio between the phylogenetic and other variance terms can be used to calculate the equivalent of Pagels lambda (See Hadfield, J. & Nakagawa, S. (2010). General quantitative genetic methods for comparative biology: phylogenies, taxonomies and multi-trait models for continuous and categorical characters. J. Evol. Biol., 23, 494–508.)"

Response: We now calculate and provide in Supplementary Information the phylogenetic variance and heritability for each model using the following functions: heritability = phylogenetic variance / (phylogenetic variance + residual variance) for gaussian distributions and heritability = phylogenetic variance / (phylogenetic variance + residual variance + 1) for probit distributions.

Reviewer #1 (Remarks to the Author):

The authors have addressed my comments adequately. I still find this an interesting and intriguing paper. Still struggling a bit with the logic of the trade-off predictions on line 97-99, but they are a reasonable staring point for the analysis.

Reviewer #2 (Remarks to the Author):

I appreciate the work of the authors on revisions and attention to comments.

I particularly appreciate the separate analyses of large phylogenetic groups and providing sample sizes. However, the results suggest less cohesiveness than the authors argue. Chiroptera, Pelecaniformes, Accipitriformes and Artiodactyla all show no significant PC. The authors cast this off as small sample size, which might be true, but also raises concern about patterns. Mammals writ large are not significant at P=0.09 and given the large sample size for mammals overall, then this is a concern. Non-passeriform birds are barely significant at p=0.044 when considering the large sample size (n=473) and suggesting that little variation is being explained. (and why is sample size for non-passeriform flyers larger than for non-passeriforms in general? There is a mistake here because when added to passeriforms it exceeds the sample size for all birds) I am surprised by the relatively small sample size of Passeriformes (n=283). This seems substantially under-sampled and under-represented given how well studied Passeriformes are and the fact that they represent more than half of the species of birds of the world. All of these issues, plus see comment below about Winger and Pegan, raise concerns about generalities argued by the authors and I think more caution is needed in conclusions.

I appreciate the authors using circles vs squares, but they are still difficult to distinguish. I still recommend also using open circles vs enclosed squares to further aid distinction.

Thank you for adding the Bartlett test.

I still disagree with your lack of consideration of nest type in birds. You argued “nest type is correlated with clutch size (cavity nesters tend to have later clutch sizes than open nesters), which is in turn is correlated with development mode”. Clutch size varies with nest type, but developmental mode does not when considered across latitudes.

Your argument that migration has survival costs: I note that a recent paper by Winger and Pegan: bioRxiv preprint doi: https://doi.org/10.1101/2020.06.27.175539 shows a counter-result among passerines. They show that shorter-distance migrants have the fastest life histories (highest reproduction and lowest survival) and that resident and long-distance migrants have similar but slower life histories. Not only is the long-distance migrant vs resident result opposite to your result, but the short- vs long-distance migrant result argues against the survival cost of migration. The authors need to address these counter-results.

Thank you for clarifications.

I appreciate the authors stressing their use of median longevity as I had similar concerns as the other reviewers about longevity vs survival. Use of median longevity is better than maximum as the authors argue. Survival would be best, but I understand the limit on sample sizes.

Reviewer #3 (Remarks to the Author):

The authors satisfactorily answered my comments and edited the text appropriately.

Reviewer #4 (Remarks to the Author):

I have reviewed the responses from the authors and I am happy that they have addressed my concerns and I have no additional comments to add. I am hence happy to recommend this paper for publication.

REVIEWER COMMENTS

Reviewer #1 (Remarks to the Author):

- The authors have addressed my comments adequately. I still find this an interesting and intriguing paper. Still struggling a bit with the logic of the trade-off predictions on line 97-99, but they are a reasonable starting point for the analysis.

RESPONSE: We thank the reviewer for the positive assessment and kind words. We have rephrased our predictions to make them more clear: 'Predictions are difficult to generate given the contradictory evidence base, but we propose that when migration has costs in terms of survival, migrants will be selected to ensure numerical compensation by reaching sexual maturity earlier or having higher annual reproductive outputs.' (lines 111-114).

Reviewer #2 (Remarks to the Author):

- I appreciate the work of the authors on revisions and attention to comments.
- I particularly appreciate the separate analyses of large phylogenetic groups and providing sample sizes. However, the results suggest less cohesiveness than the authors argue. Chiroptera, Pelecaniformes, Accipitriformes and Artiodactyla all show no significant PC. The authors cast this off as small sample size, which might be true, but also raises concern about patterns. Mammals writ large are not significant at P=0.09 and given the large sample size for mammals overall, then this is a concern. Non-passeriform birds are barely significant at p=0.044 when considering the large sample size (n=473) and suggesting that little variation is being explained. (and why is sample size for non-passeriform flyers larger than for non-passeriforms in general? There is a mistake here because when added to passeriforms it exceeds the sample size for all birds) I am surprised by the relatively small sample size of Passeriformes (n=283). This seems substantially under-sampled and under-represented given how well studied Passeriformes are and the fact that they represent more than half of the species of birds of the world. All of these issues, plus see comment below about Winger and Pegan, raise concerns about generalities argued by the authors and I think more caution is needed in conclusions.

RESPONSE: We are concerned about salami-slicing our broad-scale analysis into taxonomic components, for three reasons: (1) it loses clarity of message; (2) it risks reduction of power to detect small effects due to decreased sample sizes; (3) it confuses taxonomy with locomotory modes, which (alongside migration) is the core predictor of our analysis. Inevitably, perhaps, some taxonomic groups will provide only weak evidence for principal components of life history, or will provide principal axes that do not align with the broad-scale axes. We hope our split-analyses will promote further analyses in these groups, but our paper remains about the broad-scale phenomenon of migration across birds and mammals. We prefer for these sliced analyses to remain in supplementary material, hopefully to provoke future analyses of those groups.

Our "Mammals" analysis includes both walkers and swimmers. While walkers present a significant relationship between migration and pace of life, swimmers do not, hence non-significance for "mammals". This is part of the problem with splitting analyses by taxonomy. We now discuss in more detail the potential reasons why the relationship between pace of life and migratory strategy is not significant for swimmers (lines 258-265).

The "relatively small" sample size of Passeriformes was limited by the data availability in the Amniotes database. This dataset contains data for 5775 Passeriformes, however only 329 species have complete data for the seven life history traits that we analyse. The reduction to 283 species is due to taxonomic issues when linked with Eyres et al. 2017, BirdLife (that we use to determine the latitude), or the BirdTree project (where we obtained the phylogenetic tree). These databases often name Amniotes species as subspecies,thus several had to be removed from the dataset to

allow inclusion of biogeography and phylogeny. The Winger and Pegan source is an unrefereed pre-print that has a very narrow taxonomic focus (more detailed response below)

- I appreciate the authors using circles vs squares, but they are still difficult to distinguish. I still recommend also using open circles vs enclosed squares to further aid distinction.

RESPONSE: We agree with the reviewer and have modified the figure and legend accordingly, now we use open circles for migratory species and solid squares for non-migratory ones.

- Thank you for adding the Bartlett test.

RESPONSE: We thank the reviewer for suggesting it in the previous round of revisions, it has substantially strengthened our results.

- I still disagree with your lack of consideration of nest type in birds. You argued "nest type is correlated with clutch size (cavity nesters tend to have later clutch sizes than open nesters), which is in turn is correlated with development mode". Clutch size varies with nest type, but developmental mode does not when considered across latitudes.

RESPONSE: We agree that nest type may help to predict migratory tendency and/or life history. However, our paper is about migration, life history and locomotion across birds and mammals. Nest types are only relevant for birds, and we believe that their influence on migration is covered here (at least implicitly) via the association between nest type and life history. We agree that a comparative analysis of nest type and migration in birds would be of some interest, but that is not the topic of our study.

- Your argument that migration has survival costs: I note that a recent paper by Winger and Pegan: bioRxiv preprint doi: https://doi.org/10.1101/2020.06.27.175539 shows a counter-result among passerines. They show that shorter-distance migrants have the fastest life histories (highest reproduction and lowest survival) and that resident and long-distance migrants have similar but slower life histories. Not only is the long-distance migrant vs resident result opposite to your result, but the short- vs long-distance migrant result argues against the survival cost of migration. The authors need to address these counter-results.

RESPONSE: We agree that the results of this preprint are interesting and intriguing, however we must exert caution since the study is yet to be peer-reviewed, is has a very narrow taxonomic focus, and includes some very small sample sizes (only 6 non-migratory species, all of which are resident in harsh winter conditions, hence with perhaps inevitably poor survival).

Nevertheless, we agree that we haven't adequately discussed the possibility that the relationship between migratory strategy and pace of life differs in some groups of vertebrates, we do so in lines 274-277.

- I appreciate the authors stressing their use of median longevity as I had similar concerns as the other reviewers about longevity vs survival. Use of median longevity is better than maximum as the authors argue. Survival would be best, but I understand the limit on sample sizes.

RESPONSE: Undoubtedly including survival estimates would provide extremely valuable insights, but as the reviewer points out the sample size for this trait is quite limited, we are hopeful that in the next few years it will increase to allow its inclusion in large comparative studies.

Reviewer #3 (Remarks to the Author):

- The authors satisfactorily answered my comments and edited the text appropriately.

RESPONSE: We are very grateful to the reviewer for the positive and supportive assessment of our manuscript.

Reviewer #4 (Remarks to the Author):

- I have reviewed the responses from the authors and I am happy that they have addressed my concerns and I have no additional comments to add. I am hence happy to recommend this paper for publication.

RESPONSE: We thank the reviewer for such a positive assessment of the study.

Reviewer #2 (Remarks to the Author):

The authors largely just wave off my concerns about significance. They argue that ‘slicing’ the sample is problematic in one breath and then in the other breath explain that slicing mammals explains why they are not significant! They do not address the non-passerine low significance and that little variation is being explained.

The authors also wave off the nest type issue. It took me a while to find, but I remembered an old paper by TE Martin (1983 BioScience) where he addressed arguments that residents have lower adult survival and showed that adult survival was lower when nest type was not considered, but survival did not differ among migratory classes when nest type was considered. This is why nest type is important to include.

In the end, I am unsure the results and conclusion of this paper are correct, but it should engender follow-up tests and thereby provide a useful contribution.

Response to Reviewer #2

The authors largely just wave off my concerns about significance. They argue that 'slicing' the sample is problematic in one breath and then in the other breath explain that slicing mammals explains why they are not significant! They do not address the non-passerine low significance and that little variation is being explained.
We have added the following text "although in the latter two these effects were relatively weak." To the end of the sentence that begins on line 257 in the NoTC version of the MS.

The authors also wave off the nest type issue. It took me a while to find, but I remembered an old paper by TE Martin (1983 BioScience) where he addressed arguments that residents have lower adult survival and showed that adult survival was lower when nest type was not considered, but survival did not differ among migratory classes when nest type was considered. This is why nest type is important to include.
We have added the following text "There are other factors that can covary with migratory behaviour that could also influence one of the axes of pace of life, for example precociality of young, antipredator defence, diet and nest type (in birds). For example, some authors have shown that birds breeding in open nests have higher survival than those breeding in cavities54. Thus, it is possible such traits could potentially influence the relationship between migratory strategy and pace of life and this warrants further investigation." Lines 273-279

In the end, I am unsure the results and conclusion of this paper are correct, but it should engender follow-up tests and thereby provide a useful contribution.
We thank the referee for these comments
